# Analysis of Time to Diagnosis and Outcomes Among Adults With Primary Hyperparathyroidism

**DOI:** 10.1001/jamanetworkopen.2022.48332

**Published:** 2022-12-27

**Authors:** F. Jeffrey Lorenz, Francis Beauchamp-Perez, Andrea Manni, Thomas Chung, David Goldenberg, Neerav Goyal

**Affiliations:** 1 College of Medicine, The Pennsylvania State University, Hershey; 2 Department of Otolaryngology–Head and Neck Surgery, Penn State Hershey Medical Center, Hershey, Pennsylvania; 3 Division of Endocrinology and Diabetes, Department of Medicine, Penn State Hershey Medical Center, Hershey, Pennsylvania

## Abstract

**Importance:**

Despite access to routine laboratory evaluation, primary hyperparathyroidism (PHP) remains underdiagnosed and undertreated.

**Objective:**

To determine the consequences associated with missed diagnoses and prolonged time to diagnosis and treatment of PHP.

**Design, Setting, and Participants:**

This is a retrospective cohort study of patients older than 40 years with 2 instances of hypercalcemia during 2010 to 2020 and 3 years of follow-up. Patients were recruited from 63 health care organizations in the TriNetX Research Network. Data analysis was performed from January 2010 to September 2020.

**Exposures:**

Elevated serum calcium.

**Main Outcomes and Measures:**

Existing symptoms and diagnoses associated with PHP (osteoporosis, fractures, urolithiasis, major depressive disorder, anxiety, hypertension, gastroesophageal reflux disease, malaise or fatigue, joint pain or myalgias, constipation, insomnia, polyuria, weakness, abdominal pain, headache, nausea, amnesia, and gallstones) compared in patients deemed high-risk and without a diagnosis and matched controls, and those who experienced times from documented hypercalcemia to diagnosis and diagnosis to treatment within or beyond 1 year.

**Results:**

There were 135 034 patients analyzed (96 554 women [72%]; 28 892 Black patients [21%] and 88 010 White patients [65%]; 3608 Hispanic patients [3%] and 98 279 non-Hispanic patients [73%]; mean [SD] age, 63 [10] years). Two groups without a documented diagnosis of PHP were identified as high risk: 20 176 patients (14.9%) with parathyroid hormone greater than or equal to 50 pg/mL and 24 905 patients (18.4%) with no parathyroid hormone level obtained or recorded explanation for hypercalcemia. High-risk patients experienced significantly increased rates of all associated symptoms and diagnoses compared with matched controls. Just 9.7% of those with hypercalcemia (13 136 patients) had a diagnosis of PHP. Compared with individuals who received a diagnosis within 1 year of hypercalcemia, those whose workup exceeded 1 year had significantly increased rates of major depressive disorder, anxiety, hypertension, gastroesophageal reflux disease, malaise or fatigue, joint pain or myalgias, polyuria, weakness, abdominal pain, and headache at 3 years. The rate of osteoporosis increased from 17.1% (628 patients) to 25.4% (935 patients) over the study period in the group with delayed diagnosis. Among those with a diagnosis, 5280 patients (40.2%) underwent parathyroidectomy. Surgery beyond 1 year of diagnosis was associated with significantly increased rates of osteoporosis and hypertension at 3 years after diagnosis compared with those treated within 1 year.

**Conclusions and Relevance:**

Many patients were at high risk for PHP without a documented diagnosis. Complications in these patients, as well as those who received a diagnosis after prolonged workup or time to treatment, resulted in patient harm. System-level interventions are necessary to ensure proper diagnosis and prompt treatment of PHP.

## Key Points

**Question:**

What outcomes are associated with delays in the diagnosis and treatment of primary hyperparathyroidism (PHP)?

**Findings:**

In this large database cohort study of 135 034 patients with hypercalcemia, those who were identified as high-risk and without a diagnosis and whose duration of workup or time from diagnosis to treatment exceeded 1 year experienced increased disease sequelae.

**Meaning:**

These findings suggest that missed diagnoses and prolonged time to diagnosis and treatment of PHP are associated with increased disease burden.

## Teaser

This cohort study examines the consequences of missed diagnoses and prolonged time to diagnosis and treatment of primary hyperparathyroidism.

## Introduction

Primary hyperparathyroidism (PHP) is caused by a benign overgrowth of single or multiple parathyroid glands. It is the most common cause of hypercalcemia,^1,2^ with a prevalence of 0.1% to 1.0% and incidence of 28 cases per 100 000.^3,4,5^ A biochemical diagnosis is made in the setting of elevated calcium and nonsuppressed parathyroid hormone (PTH).^1^ Classic symptomatic PHP is associated with osteoporosis, fracture, and urolithiasis. However, the diagnosis can be missed because patients may present with nonspecific symptoms. These include memory loss, severe fatigue, depression, sleep disturbances, bone and joint pain, gastroesophageal reflux disease (GERD),^6^ and gallstones,^7,8^ which deteriorate quality of life.^9^ Although it is benign, PHP is associated with hypertension (HTN) and increased risk of cardiovascular mortality.^10^

Treatment for PHP is predominantly surgical via excision of the abnormal glands. According to the American Association of Endocrine Surgeons guidelines,^11^ surgery is indicated for any of the following criteria: (1) presence of symptoms; (2) calcium elevation greater than 1 mg/dL above the upper limit of normal; (3) osteoporosis or history of fractures; (4) nephrolithiasis, nephrocalcinosis, or 24-hour urine calcium greater than 400 mg/dL; (5) estimated glomerular filtration rate less than 60 mL/min; and (6) diagnosis at age 50 years or younger. Parathyroidectomy improves bone density, lowers fracture risk, reduces nephrolithiasis, and improves neuropsychological symptoms.^12^

Because many patients are asymptomatic and PHP is incidentally discovered, the diagnosis is frequently not actively pursued to identify candidates for surgery.^13,14,15,16,17,18,19^ In this cohort study, we sought to use the largest sample size to date from health care organizations (HCOs) across the US to determine the outcomes associated with missed diagnoses, increased time to diagnosis, and treatment delay on patients. We hypothesized that all would be associated with increased symptoms and diagnoses associated with PHP.

## Methods

Data were collected from the TriNetX Research Network (Cambridge, Massachusetts), which provided access to electronic medical records from approximately 58 million individuals aged 40 years and older from 63 HCOs across the US.^20^ TriNetX is compliant with the Health Insurance Portability and Accountability Act, only contains deidentified data, and was exempted by the Penn State institutional review board from the need for informed consent, in accordance with 45 CFR §46. This study follows the Strengthening the Reporting of Observational Studies in Epidemiology (STROBE) reporting guidelines for cohort studies. Race and ethnicity were determined from the database and were analyzed in this study to control for demographic differences between cohorts.

The database was queried using diagnosis and procedure codes to identify patients with at least 2 instances of documented hypercalcemia (serum calcium ≥10.5 mg/dL; to convert to millimoles per liter, multiply by 0.25) during 2010 to 2020. Individuals younger than 40 years were excluded to avoid differences in the definition of hypercalcemia based on age.^21^ Serum calcium of greater than or equal to 10.5 mg/dL was a conservative threshold for hypercalcemia to account for variations among laboratories.^22^ To ensure that patients were not lost to follow-up, records were included only if they had continuous enrollment in the database 3 years after initial documentation of hypercalcemia. In addition, those with history of severe kidney insufficiency (glomerular filtration rate <30 mL/min) or kidney transplant were excluded to eliminate potential confounding effects of secondary or tertiary hyperparathyroidism. Patients with hyperparathyroidism in the context of multiple endocrine neoplasia type 1 or 2A, parathyroid carcinoma, or a documented diagnosis of PHP before their first calcium level or without record of PTH levels were also excluded (Figure).

**Figure.**
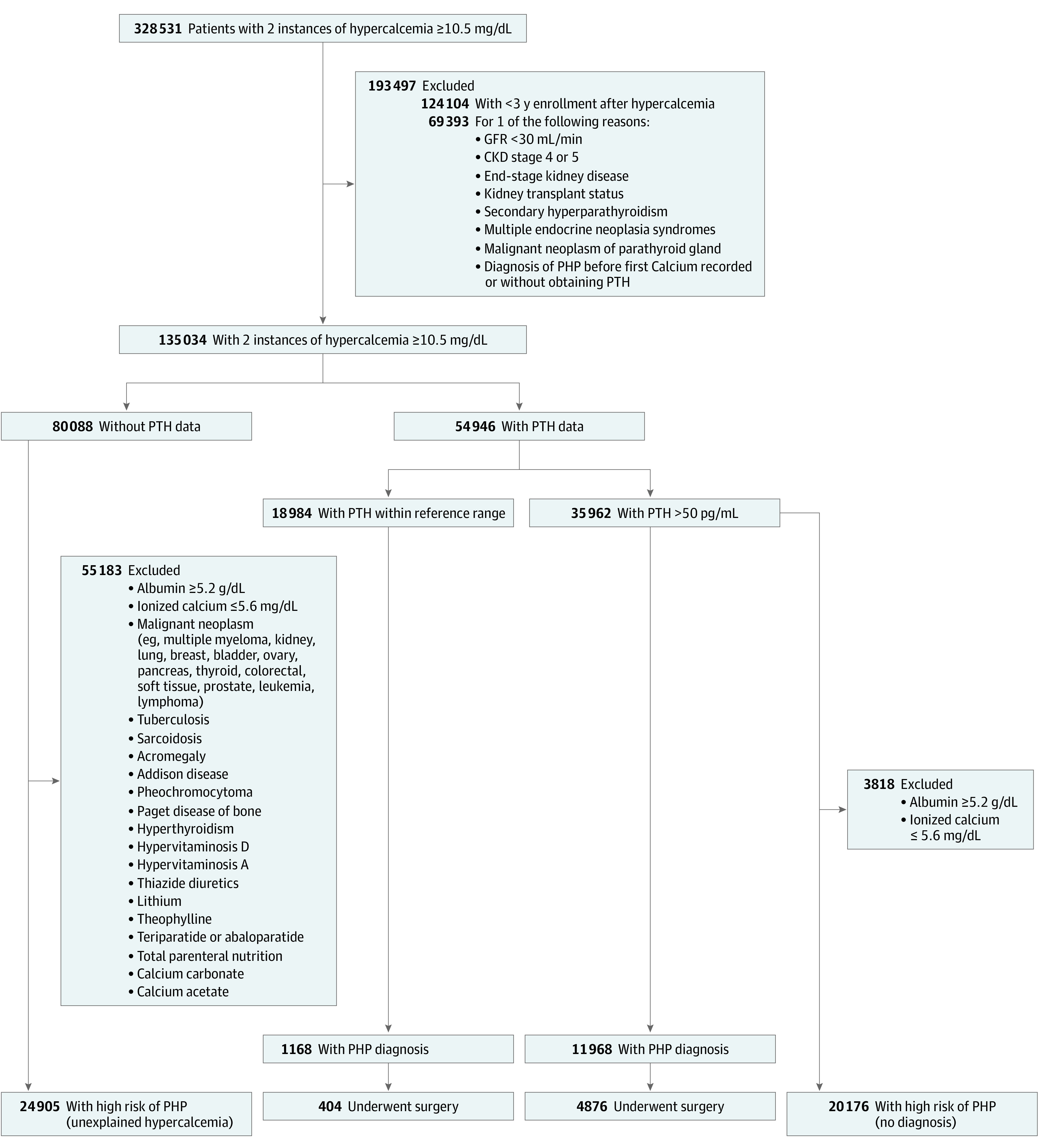
Flowchart of Cohort With Hypercalcemia To convert albumin to grams per liter, multiply by 10; calcium to millimoles per liter, multiply by 0.25; parathyroid hormone (PTH) to nanograms per liter, multiply by 1. CKD indicates chronic kidney disease; GFR, glomerular filtration rate; PHP, primary hyperparathyroidism.

Patients with hypercalcemia were grouped according to whether their PTH levels had been obtained. To identify individuals at high risk for undiagnosed PHP among those without recorded PTH, additional criteria were applied to distinguish hypercalcemia that may have been due to other causes (a complete list is shown in the Figure).^23,24^ Patients with ionized calcium within the reference range (≤5.6 mg/dL) or albumin greater than or equal to 5.2 g/dL (to convert to grams per liter, multiply by 10) were separated from high-risk groups to account for artificial elevations in serum calcium. Codes used to execute the search are available in the eMethods in Supplement 1.

Two groups were identified with a high probability of undetected PHP: (1) patients with hypercalcemia in the setting of PTH greater than or equal to 50 pg/mL (to convert to nanograms per liter, multiply by 1) and (2) those with unexplained hypercalcemia without further workup (Figure). Although the reference laboratory range for PTH is 10 to 65 pg/mL, concentrations as low as 25 pg/mL may be abnormally elevated and indicative of PHP in the setting of hypercalcemia.^25,26^ Therefore, PTH greater than or equal to 50 pg/mL was chosen as a conservative cutoff for this study.^27,28^ We assumed the latter group was at high risk for PHP because more than 90% of hypercalcemia cases are attributed to malignant neoplasms or PHP,^29^ and we excluded patients with malignant neoplasms or other potential causes of hypercalcemia. Although it is possible that some patients with undiagnosed malignant neoplasms were classified as high risk, it is much more likely that this group had undiagnosed PHP given the indolent nature of symptoms compared with those of cancer.

### Statistical Analysis

 Data analysis was performed from January 2010 to September 2020. Statistical analyses were performed within TriNetX to compare rates of symptoms and associated diagnoses between each high-risk group and controls (osteoporosis or osteopenia, fractures, urolithiasis, major depressive disorder [MDD], anxiety disorders, HTN, GERD, malaise or fatigue, joint pain or myalgias, constipation, insomnia, polyuria, weakness, abdominal pain, headache, nausea, amnesia, and gallstones).^6^ Two control groups were created by querying TriNetX for individuals without hypercalcemia with no history of elevated PTH, a diagnosis of PHP, or an exclusionary condition. The second control group also excluded patients with any of the aforementioned alternative causes of hypercalcemia. To limit confounding, the first high-risk group was compared with the first control group, whereas the second high-risk group was compared with the second control group. All comparisons were 1:1 propensity score–matched for age, sex, ethnicity, and race using nearest neighbor methods without replacement, and a caliper of 0.1 times the SD. Additional similar analyses were conducted comparing each high-risk group, patients with a confirmed diagnosis of PHP with controls, each high-risk group with those with a confirmed diagnosis, cohorts with varying PTH thresholds, patients who received a diagnosis within or beyond 1 year of the first instance of hypercalcemia, and patients treated within or beyond 1 year of diagnosis.

Outcomes were recorded during multiple time frames following the first instance of hypercalcemia: 0 to less than or equal to 1 year, 366 days to less than or equal to 2 years, and 731 days to less than or equal to 3 years. The exception was analysis of time from diagnosis to surgery, where time of diagnosis served as the index event. For this analysis, patients who did not have 3 years of follow-up after diagnosis were also excluded. To compare mean calcium and PTH levels between cohorts, 2-sided *t* tests were performed. Statistical significance was defined as *P* < .003, determined by applying a Bonferroni correction for 18 tests to an α of .05. To explore the association of region, regional data were used to compare the odds that patients identified as high-risk belonged to HCOs in the southern, northeastern, midwestern, and western US. Data analysis was performed within the TriNetX platform.

## Results

Among 135 034 patients with hypercalcemia who met the inclusion criteria (28 892 Black patients [21%] and 88 010 White patients [65%]; 3608 Hispanic patients [3%] and 98 279 non-Hispanic patients [73%]), 96 554 (72%) were female and 38 466 were (28%) male, with a mean (SD) age of 63 (10) years at first documentation of hypercalcemia. Complete demographics are described in Table 1. The number of individuals analyzed at each stage of the study are presented in the Figure.

**Table 1.** Cohort Demographics

Clinical characteristic^a^	Patients, No. (%) (N = 135 034)	
	Female (n = 96 554)^b^	Male (n = 38 466)^b^
Age, mean (SD), y	63.3 (10.3)	61.4 (10.5)
Ethnicity		
Hispanic or Latino	2573 (2.7)	1035 (2.7)
Not Hispanic or Latino	70 418 (72.9)	27 861 (72.4)
Unknown	23 563 (24.4)	9570 (24.9)
Race		
Asian	1306 (1.4)	505 (1.3)
Black or African American	21 963 (22.8)	6929 (18.0)
White	62 007 (64.2)	26 003 (67.6)
Other^c^	419 (0.4)	191 (0.5)
Unknown	10 859 (11.2)	4838 (12.6)

^a^ Sex, ethnicity, and race were classified according to documentation in electronic medical records.

^b^ Fourteen patients had an unknown sex.

^c^ Other race included American Indian or Alaska Native and Native Hawaiian or other Pacific Islander.

### Laboratory Testing and Diagnosis

Of 135 034 patients with hypercalcemia, 54 946 (40.7%) underwent further evaluation via obtaining PTH levels (Figure). Of the patients whose PTH levels were obtained, 13 136 (23.9%) had a diagnosis of PHP, 17 816 (32.4%) had PTH less than 50 pg/mL and no diagnosis, and the remaining 23 994 (43.7%) had PTH greater than or equal to 50 pg/mL yet did not receive a diagnosis. After excluding 3818 patients with elevated albumin or normal ionized calcium in the latter group, 20 176 patients (14.9% of the entire cohort) were identified as the first high-risk cohort.

Within the initial hypercalcemic group, 80 088 patients (59.3%) did not have PTH levels obtained (Figure). Among those without PTH levels available, 55 183 (68.9%) had other potential causes for hypercalcemia, whereas 24 905 (31.1%, or 18.4% of the entire cohort) lacked another explanation for hypercalcemia and had no diagnosis of PHP. This represented the second high-risk group.

### Incidence of Symptoms in High-risk Patients Without a Diagnosis

Compared with matched controls without hypercalcemia, high-risk patients with hypercalcemia and PTH greater than or equal to 50 pg/mL experienced significantly increased rates of all symptoms and diagnoses associated with PHP (Table 2). Patients with unexplained hypercalcemia without documented additional workup also experienced significantly increased rates vs controls (Table 2). Over the 3-year follow-up, the first high-risk group had significantly increased rates of most symptoms and diagnoses vs the second high-risk group, with the exception of abdominal pain, amnesia, and gallstones (eTable 1 in Supplement 1). Patients with confirmed PHP had significantly increased rates of all disease sequelae vs controls (eTable 2 in Supplement 1).

**Table 2.** Associated Diagnoses and Symptoms in Patients in High-risk Group 1 and Patients in High-risk Group 2 Compared With Matched Controls

Time and diagnosis	Patients, No. (%) after matching		OR (95% CI)	*P* value^b^	Patients, No. (%) after matching		OR (95% CI)	*P* value^b^
	High-risk group 1 (n = 20 175)^a^	Matched controls (n = 20 175)			High-risk group 2 (n = 24 905)^c^	Matched controls (n = 24 905)		
0 to ≤1 y								
Osteopenia or osteoporosis	2662 (13.2)	1099 (5.4)	2.64 (2.45-2.84)	<.001	1423 (5.7)	1065 (4.3)	1.36 (1.25-1.47)	<.001
Fractures	719 (3.6)	478 (2.4)	1.52 (1.35-1.71)	<.001	691 (2.8)	603 (2.4)	1.15 (1.03-1.29)	.01
Urolithiasis	771 (3.8)	281 (1.4)	2.81 (2.45-3.23)	<.001	746 (3.0)	378 (1.5)	2.00 (1.77-2.27)	<.001
MDD	2009 (10.0)	1393 (6.9)	1.49 (1.39-1.60)	<.001	2507 (10.1)	1565 (6.3)	1.67 (1.56-1.78)	<.001
Anxiety disorders	1920 (9.5)	1347 (6.7)	1.47 (1.37-1.58)	<.001	2481 (10.0)	1689 (6.8)	1.52 (1.43-1.62)	<.001
HTN	9107 (45.1)	6231 (30.9)	1.84 (1.77-1.92)	<.001	8872 (35.6)	5795 (23.3)	1.83 (1.76-1.90)	<.001
GERD	2952 (14.6)	2047 (10.1)	1.52 (1.43-1.61)	<.001	3186 (12.8)	2241 (9.0)	1.48 (1.40-1.57)	<.001
Malaise or fatigue	2065 (10.2)	1328 (6.6)	1.62 (1.51-1.74)	<.001	2086 (8.4)	1455 (5.8)	1.47 (1.38-1.58)	<.001
Joint pain or myalgias	3461 (17.2)	2385 (11.8)	1.55 (1.46-1.63)	<.001	3745 (15.0)	2834 (11.4)	1.38 (1.31-1.45)	<.001
Constipation	1247 (6.2)	719 (3.6)	1.78 (1.54-1.96)	<.001	1334 (5.4)	687 (2.8)	2.00 (1.82-2.19)	<.001
Insomnia	885 (4.4)	524 (2.6)	1.72 (1.54-1.92)	<.001	937 (3.8)	570 (2.3)	1.67 (1.50-1.86)	<.001
Polyuria	874 (4.3)	506 (2.5)	1.76 (1.58-1.97)	<.001	905 (3.6)	607 (2.4)	1.51 (1.36-1.68)	<.001
Weakness	1374 (6.8)	872 (4.3)	1.62 (1.48-1.77)	<.001	1413 (5.7)	891 (3.6)	1.62 (1.49-1.77)	<.001
Abdominal pain	2142 (10.6)	1573 (7.8)	1.41 (1.31-1.50)	<.001	3067 (12.3)	1916 (7.7)	1.69 (1.59-1.79)	<.001
Headache	1104 (5.5)	794 (3.9)	1.41 (1.29-1.55)	<.001	1320 (5.3)	891 (3.6)	1.51 (1.38-1.65)	<.001
Nausea	814 (4.0)	498 (2.5)	1.66 (1.48-1.86)	<.001	1013 (4.1)	567 (2.3)	1.82 (1.64-2.02)	<.001
Amnesia	738 (3.7)	506 (2.5)	1.48 (1.32-1.66)	<.001	992 (4.0)	566 (2.3)	1.78 (1.61-1.98)	<.001
Gallstones	373 (1.8)	217 (1.1)	1.73 (1.46-2.05)	<.001	480 (1.9)	282 (1.1)	1.72 (1.48-1.98)	<.001
366 d to ≤2 y								
Osteopenia or osteoporosis	2718 (13.5)	1070 (5.3)	2.78 (2.58-2.99)	<.001	1461 (5.9)	1002 (4.0)	1.49 (1.37-1.61)	<.001
Fractures	647 (3.2)	367 (1.8)	1.79 (1.57-2.04)	<.001	581 (2.3)	370 (1.5)	1.58 (1.39-1.81)	<.001
Urolithiasis	651 (3.2)	198 (1.0)	3.36 (2.87-3.95)	<.001	539 (2.2)	225 (0.9)	2.43 (2.08-2.84)	<.001
MDD	1909 (9.5)	1164 (5.8)	1.71 (1.58-1.84)	<.001	2088 (8.4)	1213 (4.9)	1.79 (1.66-1.92)	<.001
Anxiety disorders	1740 (8.6)	1105 (5.5)	1.63 (1.51-1.76)	<.001	2056 (8.3)	1379 (5.5)	1.54 (1.43-1.65)	<.001
HTN	8940 (44.3)	5057 (25.1)	2.38 (2.28-2.48)	<.001	7814 (31.4)	4327 (17.4)	2.17 (2.08-2.27)	<.001
GERD	2690 (13.3)	1606 (8.0)	1.78 (1.67-1.90)	<.001	2676 (10.7)	1643 (6.6)	1.70 (1.60-1.82)	<.001
Malaise or fatigue	1531 (7.6)	898 (4.5)	1.76 (1.62-1.92)	<.001	1487 (6.0)	958 (3.8)	1.59 (1.46-1.73)	<.001
Joint pain or myalgias	3327 (16.5)	2004 (9.9)	1.79 (1.69-1.90)	<.001	3167 (12.7)	2285 (9.2)	1.44 (1.36-1.53)	<.001
Constipation	1001 (5.0)	472 (2.3)	2.18 (1.95-2.44)	<.001	935 (3.8)	443 (1.8)	2.15 (1.92-2.42)	<.001
Insomnia	725 (3.6)	395 (2.0)	1.86 (1.65-2.11)	<.001	754 (3.0)	418 (1.7)	1.83 (1.62-2.06)	<.001
Polyuria	786 (3.9)	420 (2.1)	1.91 (1.69-2.15)	<.001	724 (2.9)	515 (2.1)	1.42 (1.27-1.59)	<.001
Weakness	948 (4.7)	547 (2.8)	1.77 (1.59-1.97)	<.001	949 (3.8)	548 (2.2)	1.76 (1.58-1.96)	<.001
Abdominal pain	1698 (8.4)	949 (4.7)	1.86 (1.72-2.02)	<.001	2020 (8.1)	1102 (4.4)	1.91 (1.77-2.06)	<.001
Headache	923 (4.6)	505 (2.5)	1.87 (1.67-2.09)	<.001	890 (3.6)	543 (2.2)	1.66 (1.49-1.85)	<.001
Nausea	598 (3.0)	292 (1.4)	2.08 (1.81-2.40)	<.001	614 (2.5)	277 (1.1)	2.25 (1.95-2.59)	<.001
Amnesia	635 (3.1)	373 (1.8)	1.73 (1.52-1.96)	<.001	683 (2.7)	336 (1.3)	2.06 (1.81-2.35)	<.001
Gallstones	265 (1.3)	124 (0.6)	2.15 (1.74-2.67)	<.001	264 (1.1)	107 (0.4)	2.48 (1.98-3.11)	<.001
731 d to ≤3 y								
Osteopenia or osteoporosis	2945 (14.6)	1254 (6.2)	2.58 (2.41-2.76)	<.001	1635 (6.6)	1117 (4.5)	1.50 (1.38-1.62)	<.001
Fractures	679 (3.4)	418 (2.1)	1.65 (1.46-1.86)	<.001	616 (2.5)	424 (1.7)	1.46 (1.29-1.66)	<.001
Urolithiasis	644 (3.2)	175 (0.9)	3.77 (3.19-4.46)	<.001	549 (2.2)	230 (0.9)	2.42 (2.07-2.82)	<.001
MDD	2024 (10.0)	1200 (5.9)	1.76 (1.64-1.90)	<.001	2087 (8.4)	1247 (5.0)	1.74 (1.61-1.87)	<.001
Anxiety disorders	1835 (9.1)	1163 (5.8)	1.64 (1.52-1.77)	<.001	2175 (8.7)	1433 (5.8)	1.57 (1.46-1.68)	<.001
HTN	9124 (45.2)	5346 (26.5)	2.29 (2.20-2.39)	<.001	8121 (32.6)	4670 (18.8)	2.10 (2.01-2.19)	<.001
GERD	2800 (13.9)	1760 (8.7)	1.69 (1.58-1.80)	<.001	2801 (11.2)	1803 (7.2)	1.62 (1.53-1.73)	<.001
Malaise or fatigue	1574 (7.8)	917 (4.5)	1.78 (1.63-1.93)	<.001	1488 (6.0)	1028 (4.1)	1.48 (1.36-1.60)	<.001
Joint pain or myalgias	3285 (16.3)	2082 (10.3)	1.69 (1.59-1.79)	<.001	3246 (13.0)	2361 (9.5)	1.43 (1.35-1.51)	<.001
Constipation	1017 (5.0)	511 (2.5)	2.04 (1.83-2.28)	<.001	948 (3.8)	513 (2.1)	1.88 (1.69-2.10)	<.001
Insomnia	774 (3.8)	436 (2.2)	1.81 (1.60-2.03)	<.001	765 (3.1)	484 (1.9)	1.60 (1.43-1.79)	<.001
Polyuria	849 (4.2)	511 (2.5)	1.69 (1.51-1.89)	<.001	737 (3.0)	525 (2.1)	1.42 (1.26-1.59)	<.001
Weakness	1034 (5.1)	577 (2.9)	1.84 (1.65-2.04)	<.001	914 (3.7)	587 (2.4)	1.58 (1.42-1.75)	<.001
Abdominal pain	1725 (8.6)	1041 (5.2)	1.72 (1.59-1.86)	<.001	2056 (8.3)	1145 (4.6)	1.87 (1.73-2.01)	<.001
Headache	848 (4.2)	480 (2.4)	1.80 (1.61-2.02)	<.001	892 (3.6)	542 (2.2)	1.67 (1.50-1.86)	<.001
Nausea	576 (2.9)	289 (1.3)	2.02 (1.75-2.33)	<.001	575 (2.3)	303 (1.2)	1.92 (1.67-2.21)	<.001
Amnesia	681 (3.4)	404 (2.0)	1.71 (1.51-1.94)	<.001	737 (3.0)	379 (1.5)	1.97 (1.74-2.24)	<.001
Gallstones	267 (1.3)	132 (0.7)	2.04 (1.65-2.51)	<.001	296 (1.2)	144 (0.6)	2.07 (1.69-2.53)	<.001
Entire study period (0-3 y)								
Osteopenia or osteoporosis	4447 (22.0)	2221 (11.0)	2.29 (2.16-2.42)	<.001	2758 (11.1)	2012 (8.1)	1.42 (1.33-1.51)	<.001
Fractures	1450 (7.2)	1044 (5.2)	1.42 (1.31-1.54)	<.001	1501 (6.0)	1166 (4.7)	1.31 (1.21-1.41)	<.001
Urolithiasis	1262 (6.3)	470 (2.3)	2.80 (2.51-3.12)	<.001	1315 (5.3)	637 (2.6)	2.12 (1.93-2.34)	<.001
MDD	3218 (16.0)	2253 (11.2)	1.51 (1.43-1.60)	<.001	3824 (15.4)	2491 (10.0)	1.63 (1.55-1.72)	<.001
Anxiety disorders	3097 (15.4)	2177 (10.8)	1.50 (1.41-1.59)	<.001	3889 (15.6)	2772 (11.1)	1.48 (1.40-1.56)	<.001
HTN	11 187 (55.5)	8114 (40.2)	1.85 (1.78-1.93)	<.001	11 436 (45.9)	7813 (31.4)	1.86 (1.79-1.93)	<.001
GERD	4602 (22.8)	3430 (17.0)	1.44 (1.33-1.52)	<.001	5035 (20.2)	3588 (14.4)	1.51 (1.44-1.58)	<.001
Malaise or fatigue	3687 (18.3)	2428 (12.0)	1.63 (1.54-1.73)	<.001	3812 (15.3)	2716 (10.9)	1.48 (1.40-1.56)	<.001
Joint pain or myalgias	6266 (31.1)	4591 (22.8)	1.53 (1.46-1.60)	<.001	6786 (27.2)	5395 (21.7)	1.35 (1.30-1.41)	<.001
Constipation	2335 (11.6)	1316 (6.5)	1.88 (1.75-2.01)	<.001	2447 (9.8)	1306 (5.2)	1.97 (1.84-2.12)	<.001
Insomnia	1592 (7.9)	994 (4.9)	1.65 (1.52-1.79)	<.001	1675 (6.7)	1034 (4.2)	1.67 (1.54-1.80)	<.001
Polyuria	1905 (9.4)	1205 (6.0)	1.64 (1.52-1.77)	<.001	1871 (7.5)	1319 (5.3)	1.45 (1.35-1.56)	<.001
Weakness	2522 (12.5)	1670 (8.3)	1.58 (1.52-1.77)	<.001	2639 (10.6)	1690 (6.8)	1.63 (1.53-1.74)	<.001
Abdominal pain	3979 (19.7)	2844 (14.1)	1.50 (1.42-1.58)	<.001	5188 (20.8)	3406 (13.7)	1.66 (1.58-1.74)	<.001
Headache	2155 (10.7)	1453 (7.2)	1.54 (1.44-1.65)	<.001	2462 (9.9)	1636 (6.6)	1.56 (1.46-1.67)	<.001
Nausea	1518 (7.5)	919 (4.6)	1.71 (1.57-1.86)	<.001	1795 (7.2)	1009 (4.1)	1.84 (1.70-1.99)	<.001
Amnesia	1484 (7.4)	965 (4.8)	1.58 (1.45-1.72)	<.001	1852 (7.4)	1005 (4.0)	1.91 (1.77-2.07)	<.001
Gallstones	708 (3.5)	442 (2.2)	1.62 (1.44-1.83)	<.001	866 (3.5)	475 (1.9)	1.85 (1.65-2.08)	<.001

Abbreviations: GERD, gastroesophageal reflux disease; HTN, hypertension; MDD, major depressive disorder; OR, odds ratio.

^a^ High risk-group 1 included a total of 20 176 patients with hypercalcemia and parathyroid hormone greater than or equal to 50 pg/mL.

^b^ Statistical significance is defined as *P* < .003.

^c^ High-risk group 2 included 24 905 patients with unexplained hypercalcemia and no further workup.

Compared with patients with a diagnosis, over the entire study period (0-3 years), the first high-risk group had significantly decreased rates of osteoporosis, urolithiasis, GERD, malaise and fatigue, polyuria, and weakness; however, there were no differences in other symptoms (eTable 3 in Supplement 1). There were also individual years when the first high-risk group experienced increased rates of certain symptoms (anxiety, insomnia, and HTN). The first high-risk group had 40% and 50% decreased odds of osteoporosis and urolithiasis, respectively, but had less than a 10% decrease or increase of other disease sequelae vs patients with a diagnosis. Conversely, the second high-risk group experienced significantly decreased rates of all disease sequelae vs those with confirmed PHP (eTable 3 in Supplement 1).

Documented PHP was associated with significantly elevated calcium levels compared with both the first high-risk group whose PTH levels were obtained (10.90 vs 10.80 mg/dL; difference, 0.10 mg/dL; 95% CI, 0.09-0.11 mg/dL; *P* < .001) and second high-risk group whose PTH levels were not obtained (10.90 vs 10.70 mg/dL; difference, 0.20 mg/dL; 95% CI, 0.19-0.21 mg/dL; *P* < .001). Patients with a diagnosis also had significantly elevated PTH values vs the first high-risk group (107.00 vs 93.00 pg/mL; difference, 14.00 pg/mL; 95% CI, 12.20-15.80 pg/mL; *P* < .001) (eTable 4 in Supplement 1). Furthermore, 1371 patients (6.8%) in high-risk group 1, 1402 patients (5.6%) in high-risk group 2, and 1199 (9.1%) of those with a confirmed diagnosis had a normal calcium level that later became elevated.

### Altering PTH Cutoffs

Stratifying PTH cutoffs to 40, 50, 65, and 100 pg/mL was not associated with proportionate increases in disease sequelae for the first high-risk group as well as the group with a diagnosis. The high-risk group with stratified PTH values was compared with matched controls (eTable 5 and eTable 6 in Supplement 1), the overall group who received a diagnosis (eTable 7 and eTable 8 in Supplement 1), and the group with a diagnosis stratified by PTH levels (eTable 9 and eTable 10 in Supplement 1).

### Rates of Missed Diagnoses by Region

Among the total cohort of patients with hypercalcemia (135 034 patients), 55 243 (40.9%) records were from patients who sought care in the South, 36 725 (27.2%) were in the Northeast, 25 955 (19.2%) were in the Midwest, 8737 (6.5%) were in the West, and 8374 (6.2%) were in an unknown region or outside the US. In each region, 18 114 patients (32.8%) in the South, 11 683 (31.8%) in the Northeast, 6986 (26.9%) in the Midwest, and 2477 (28.3%) in the West were considered high-risk and without a diagnosis. Compared with the South, patients in the Northeast (odds ratio [OR], 0.95; 95% CI, 0.90-1.00; *P* = .002), Midwest (OR, 0.75; 95% CI, 0.70-0.80; *P* < .001), and West (OR, 0.81; 95% CI, 0.80-0.90; *P* < .001) were less likely to be high-risk and not have a diagnosis.

### Association of Time to Diagnosis With Symptoms

Just 13 136 patients (9.7%) with hypercalcemia received a diagnosis of PHP (Figure). Of these individuals, 3686 (28.1%) received a diagnosis within 1 year and 9450 (71.9%) received a diagnosis after 1 year of their first instance of hypercalcemia. Patients who received a diagnosis within the first year were more likely to be symptomatic during that time; they had increased rates of osteoporosis, urolithiasis, MDD, anxiety, HTN, malaise and fatigue, constipation, weakness, abdominal pain, headache, nausea, and amnesia. However, by 2 to 3 years, those with workups exceeding 1 year had significantly increased rates of MDD, anxiety, HTN, GERD, malaise and fatigue, joint pain and myalgias, polyuria, weakness, abdominal pain, and headache (Table 3). Of note, rates of osteoporosis within this delayed group increased from 17.1% (628 patients) to 25.4% (935 patients) over the course of the study period (Table 3). The cohort who received a diagnosis within 1 year had significantly increased mean calcium (11.10 vs 10.80 mg/dL; difference, 0.30 mg/dL; 95% CI, 0.28-0.32 mg/dL; *P* < .001) and PTH (121 vs 102 pg/mL; difference, 19 pg/mL; 95% CI, 15-23 pg/mL; *P* < .001) values compared with those who received a diagnosis beyond 1 year (eTable 4 in Supplement 1).

**Table 3.** Diagnoses and Symptoms in 9450 Patients Who Received a Diagnosis >1 Year vs 3686 Patients Who Received a Diagnosis ≤1 Year After Laboratory Abnormality

Time and diagnosis	Patients, No. (%) after matching		OR (95% CI)	*P* value^a^
	Diagnosis >1 y after hypercalcemia (n = 3681)	Diagnosis ≤1 y after hypercalcemia (n = 3681)		
0 to ≤1 y				
Osteopenia or osteoporosis	628 (17.1)	1257 (34.1)	0.40 (0.36-0.44)	<.001
Fractures	156 (4.2)	168 (4.6)	0.93 (0.74-1.16)	.50
Urolithiasis	255 (6.9)	552 (15.0)	0.42 (0.36-0.49)	<.001
MDD	438 (11.9)	594 (16.1)	0.70 (0.62-0.80)	<.001
Anxiety disorders	381 (10.4)	494 (13.4)	0.75 (0.65-0.86)	<.001
HTN	1878 (51.0)	2065 (56.1)	0.82 (0.74-0.89)	<.001
GERD	676 (18.4)	767 (20.8)	0.86 (0.76-0.96)	.008
Malaise or fatigue	463 (12.6)	665 (18.1)	0.65 (0.57-0.74)	<.001
Joint pain or myalgias	788 (21.4)	807 (21.9)	0.97 (0.87-1.08)	.59
Constipation	269 (7.3)	405 (11.0)	0.64 (0.54-0.75)	<.001
Insomnia	168 (4.6)	177 (4.8)	0.95 (0.76-1.18)	.62
Polyuria	209 (5.7)	266 (7.2)	0.77 (0.64-0.93)	.007
Weakness	332 (9.0)	435 (11.8)	0.74 (0.64-0.86)	<.001
Abdominal pain	468 (12.7)	561 (15.2)	0.81 (0.71-0.93)	.002
Headache	258 (7.0)	327 (8.9)	0.77 (0.65-0.92)	.003
Nausea	191 (5.2)	252 (6.8)	0.75 (0.61-0.90)	.003
Amnesia	135 (3.7)	288 (7.8)	0.44 (0.36-0.55)	<.001
Gallstones	81 (2.2)	106 (2.9)	0.76 (0.57-1.02)	.06
366 d to ≤2 y				
Osteopenia or osteoporosis	786 (21.4)	877 (23.8)	0.87 (0.78-0.97)	.01
Fractures	158 (4.3)	119 (3.2)	1.34 (1.05-1.71)	.02
Urolithiasis	245 (6.7)	300 (8.1)	0.80 (0.67-0.96)	.01
MDD	452 (12.3)	413 (11.2)	1.11 (0.95-1.28)	.16
Anxiety disorders	382 (10.4)	337 (9.2)	1.15 (0.99-1.34)	.08
HTN	1944 (52.8)	1568 (42.6)	1.51 (1.38-1.65)	<.001
GERD	699 (19.0)	541 (14.7)	1.36 (1.20-1.54)	<.001
Malaise or fatigue	400 (10.9)	345 (9.4)	1.18 (1.01-1.37)	.03
Joint pain or myalgias	754 (20.5)	634 (17.2)	1.24 (1.10-1.37)	<.001
Constipation	247 (6.7)	230 (6.2)	1.08 (0.90-1.30)	.42
Insomnia	147 (4.0)	141 (3.8)	1.04 (0.83-1.32)	.72
Polyuria	171 (4.6)	171 (4.6)	1.00 (0.81-1.24)	>.99
Weakness	269 (7.3)	221 (6.0)	1.23 (1.03-1.48)	.02
Abdominal pain	434 (11.8)	376 (10.2)	1.18 (1.02-1.36)	.03
Headache	226 (6.1)	187 (5.1)	1.22 (1.00-1.49)	.05
Nausea	141 (3.8)	125 (3.4)	1.13 (0.89-1.45)	.32
Amnesia	125 (3.4)	154 (4.2)	0.81 (0.63-1.02)	.08
Gallstones	56 (1.5)	65 (1.8)	0.86 (0.60-1.23)	.41
731 d to ≤3 y				
Osteopenia or osteoporosis	935 (25.4)	866 (23.5)	1.11 (1.00-1.23)	.06
Fractures	163 (4.4)	118 (3.2)	1.40 (1.10-1.78)	.006
Urolithiasis	293 (8.0)	244 (6.6)	1.22 (1.02-1.45)	.03
MDD	479 (13.0)	370 (10.1)	1.34 (1.16-1.45)	<.001
Anxiety disorders	436 (11.8)	334 (9.1)	1.35 (1.16-1.57)	<.001
HTN	1980 (53.8)	1480 (40.2)	1.73 (1.58-1.90)	<.001
GERD	729 (19.8)	509 (13.8)	1.54 (1.36-1.74)	<.001
Malaise or fatigue	443 (12.0)	310 (8.4)	1.49 (1.28-1.73)	<.001
Joint pain or myalgias	802 (21.8)	620 (16.8)	1.38 (1.22-1.55)	<.001
Constipation	251 (6.8)	199 (5.4)	1.28 (1.06-1.55)	.01
Insomnia	180 (4.9)	139 (3.8)	1.31 (1.05-1.64)	.02
Polyuria	211 (5.7)	134 (3.6)	1.61 (1.29-2.01)	<.001
Weakness	281 (7.6)	176 (4.8)	1.65 (1.36-2.00)	<.001
Abdominal pain	435 (11.8)	294 (8.0)	1.54 (1.32-1.80)	<.001
Headache	233 (6.3)	172 (4.7)	1.38 (1.13-1.69)	.002
Nausea	147 (4.0)	120 (3.3)	1.23 (0.97-1.58)	.09
Amnesia	151 (4.1)	140 (3.8)	1.08 (0.86-1.37)	.51
Gallstones	61 (1.7)	47 (1.3)	1.30 (0.89-1.91)	.17
1-3 y				
Osteopenia or osteoporosis	1179 (32.0)	1163 (31.6)	1.02 (0.99-1.13)	.69
Fractures	253 (6.9)	194 (5.3)	1.33 (1.09-1.61)	.004
Urolithiasis	395 (10.7)	397 (10.8)	0.99 (0.86-1.15)	.94
MDD	638 (17.3)	554 (15.1)	1.18 (1.05-1.34)	.008
Anxiety disorders	577 (15.7)	495 (13.4)	1.20 (1.05-1.36)	.007
HTN	2267 (61.6)	1814 (49.3)	1.65 (1.50-1.81)	<.001
GERD	973 (26.4)	743 (20.2)	1.42 (1.27-1.58)	<.001
Malaise or fatigue	697 (18.9)	539 (14.6)	1.36 (1.20-1.54)	<.001
Joint pain or myalgias	1168 (31.7)	959 (26.1)	1.32 (1.19-1.46)	<.001
Constipation	416 (11.3)	350 (9.5)	1.21 (1.04-1.41)	.01
Insomnia	243 (6.6)	214 (5.8)	1.15 (0.95-1.38)	.16
Polyuria	329 (8.9)	269 (7.3)	1.25 (1.05-1.47)	.01
Weakness	465 (12.6)	341 (9.3)	1.42 (1.22-1.64)	<.001
Abdominal pain	713 (19.4)	552 (15.0)	1.36 (1.21-1.54)	<.001
Headache	386 (10.5)	301 (8.2)	1.32 (1.12-1.54)	<.001
Nausea	256 (7.0)	209 (5.7)	1.24 (1.03-1.50)	.02
Amnesia	227 (6.2)	241 (6.5)	0.94 (0.78-1.13)	.50
Gallstones	98 (2.7)	92 (2.5)	1.07 (0.80-1.42)	.66

Abbreviations: GERD, gastroesophageal reflux disease; HTN, hypertension; MDD, major depressive disorder; OR, odds ratio.

^a^ Statistical significance is defined as *P* < .003.

### Association of Time From Diagnosis to Surgery With Symptoms

Only 5280 (40.2%) of all patients with PHP diagnosed underwent parathyroidectomy (Figure). Among them, 4361 (82.6%) were treated within 1 year of diagnosis and 919 (17.4%) were treated beyond 1 year. There were 1398 patients who were then excluded from analysis because of lack of follow-up 3 years after diagnosis. Those treated within 1 year of diagnosis experienced higher rates of MDD, GERD, and constipation during that same year and had reductions in all symptoms after surgery. Conversely, delayed surgery beyond 1 year resulted in significantly increased rates of osteoporosis and HTN by 2 to 3 years after diagnosis compared with those treated within 1 year (Table 4).

**Table 4.** Diagnoses and Symptoms in 919 Patients Who Underwent Surgery >1 Year vs 4361 Patients Who Underwent Surgery ≤1 Year After Diagnosis of Primary Hyperparathyroidism^a^

Time and diagnosis	Patients, No. (%) after matching		OR (95% CI)	*P* value^b^
	Surgery >1 y after diagnosis (n = 748)	Surgery ≤1 y after diagnosis (n = 748)		
0 to ≤1 y				
Osteopenia or osteoporosis	277 (37.0)	299 (40.0)	0.88 (0.72-1.09)	.24
Fractures	42 (5.6)	36 (4.8)	1.18 (0.75-1.86)	.49
Urolithiasis	109 (14.6)	131 (17.5)	0.80 (0.61-1.06)	.12
MDD	103 (13.8)	154 (20.6)	0.62 (0.47-0.81)	<.001
Anxiety disorders	80 (10.7)	113 (15.1)	0.67 (0.50-0.91)	.01
HTN	436 (58.3)	471 (63.0)	0.82 (0.67-1.01)	.06
GERD	154 (20.6)	235 (31.4)	0.57 (0.45-0.72)	<.001
Malaise or fatigue	112 (15.0)	137 (18.3)	0.79 (0.60-1.03)	.08
Joint pain or myalgias	191 (25.5)	200 (26.7)	0.94 (0.75-1.18)	.60
Constipation	62 (8.3)	97 (13.0)	0.61 (0.43-0.85)	.003
Insomnia	48 (6.4)	40 (5.3)	1.21 (0.79-1.87)	.38
Polyuria	58 (7.8)	54 (7.2)	1.08 (0.74-1.59)	.69
Weakness	67 (9.0)	74 (9.9)	0.90 (0.63-1.27)	.54
Abdominal pain	124 (16.6)	108 (14.4)	1.18 (0.89-1.56)	.25
Headache	40 (5.3)	68 (9.1)	0.57 (0.38-0.85)	.005
Nausea	40 (5.3)	48 (6.4)	0.82 (0.54-1.27)	.38
Amnesia	42 (5.6)	58 (7.8)	0.71 (0.47-1.07)	.10
Gallstones	18 (2.4)	21 (2.8)	0.85 (0.45-1.62)	.63
366 d to ≤2 y				
Osteopenia or osteoporosis	258 (34.5)	210 (28.1)	1.35 (1.08-1.68)	.007
Fractures	33 (4.4)	22 (2.9)	1.52 (0.88-2.64)	.13
Urolithiasis	90 (12.0)	65 (8.7)	1.44 (1.03-2.01)	.03
MDD	114 (15.2)	104 (13.9)	1.11 (0.84-1.48)	.46
Anxiety disorders	89 (11.9)	75 (10.0)	1.21 (0.88-1.68)	.25
HTN	441 (59.0)	353 (47.2)	1.61 (1.31-1.97)	<.001
GERD	185 (24.7)	138 (18.4)	1.45 (1.13-1.86)	.003
Malaise or fatigue	96 (12.8)	75 (10.0)	1.32 (0.96-1.82)	.09
Joint pain or myalgias	175 (23.4)	148 (19.8)	1.24 (0.97-1.59)	.09
Constipation	69 (9.2)	42 (5.6)	1.71 (1.15-2.54)	.008
Insomnia	32 (4.3)	37 (4.9)	0.86 (0.53-1.39)	.54
Polyuria	57 (7.6)	36 (4.8)	1.63 (1.06-2.51)	.02
Weakness	49 (6.6)	30 (4.0)	1.68 (1.05-2.67)	.03
Abdominal pain	108 (14.4)	83 (11.1)	1.35 (1.00-1.84)	.05
Headache	53 (7.1)	37 (4.9)	1.47 (0.95-2.26)	.08
Nausea	42 (5.6)	28 (3.7)	1.53 (0.94-2.50)	.09
Amnesia	28 (3.7)	28 (3.7)	1.00 (0.59-1.71)	>.99
Gallstones	19 (2.5)	14 (1.9)	1.37 (0.68-2.75)	.38
731 d to ≤3 y				
Osteopenia or osteoporosis	288 (38.5)	181 (24.2)	1.96 (1.57-2.75)	<.001
Fractures	23 (3.1)	28 (3.7)	0.82 (0.47-1.43)	.48
Urolithiasis	72 (9.6)	63 (8.4)	1.16 (0.81-1.65)	.42
MDD	106 (14.2)	96 (12.8)	1.12 (0.83-1.51)	.45
Anxiety disorders	88 (11.8)	81 (10.8)	1.10 (0.80-1.51)	.57
HTN	433 (57.9)	361 (48.3)	1.47 (1.20-1.81)	<.001
GERD	161 (21.5)	148 (19.8)	1.11 (0.87-1.43)	.41
Malaise or fatigue	92 (12.3)	74 (9.9)	1.28 (0.92-1.77)	.14
Joint pain or myalgias	194 (25.9)	160 (21.4)	1.29 (1.01-1.64)	.04
Constipation	56 (7.5)	39 (5.2)	1.47 (0.97-2.24)	.07
Insomnia	39 (5.2)	35 (4.7)	1.12 (0.70-1.79)	.63
Polyuria	43 (5.7)	44 (5.9)	0.98 (0.63-1.51)	.91
Weakness	47 (6.3)	32 (4.3)	1.50 (0.95-2.38)	.08
Abdominal pain	88 (11.8)	66 (8.8)	1.38 (0.98-1.93)	.06
Headache	43 (5.7)	46 (6.1)	0.93 (0.61-1.43)	.74
Nausea	28 (3.7)	36 (4.8)	0.77 (0.46-1.27)	.31
Amnesia	29 (3.9)	28 (3.7)	1.04 (0.61-1.76)	.89
Gallstones	16 (2.1)	16 (2.1)	1.00 (0.50-2.02)	>.99
1-3 y				
Osteopenia or osteoporosis	362 (48.4)	257 (34.4)	1.79 (1.46-2.21)	<.001
Fractures	46 (6.1)	40 (5.3)	1.16 (0.75-1.79)	.51
Urolithiasis	114 (15.2)	88 (11.8)	1.35 (1.00-1.82)	.05
MDD	149 (19.9)	136 (18.2)	1.12 (0.87-1.45)	.39
Anxiety disorders	130 (17.4)	113 (15.1)	1.18 (0.90-1.56)	.23
HTN	496 (66.3)	407 (54.4)	1.65 (1.34-2.03)	<.001
GERD	234 (31.3)	191 (25.5)	1.33 (1.06-1.66)	.01
Malaise or fatigue	155 (20.7)	122 (16.3)	1.34 (1.03-74)	.03
Joint pain or myalgias	280 (37.4)	234 (31.3)	1.31 (1.06-1.63)	.01
Constipation	104 (13.9)	63 (8.4)	1.76 (1.26-2.45)	<.001
Insomnia	54 (7.2)	58 (7.8)	0.93 (0.63-1.36)	.69
Polyuria	88 (11.8)	67 (9.0)	1.36 (0.97-1.90)	.07
Weakness	86 (11.5)	55 (7.4)	1.64 (1.15-2.33)	.006
Abdominal pain	162 (21.7)	127 (17.0)	1.35 (1.04-1.75)	.02
Headache	87 (11.6)	71 (9.5)	1.26 (0.90-1.73)	.18
Nausea	66 (8.8)	56 (7.5)	1.20 (0.83-1.73)	.34
Amnesia	47 (6.3)	45 (6.0)	1.05 (0.69-1.60)	.83
Gallstones	30 (4.0)	23 (3.1)	1.31 (0.76-2.29)	.33

Abbreviations: GERD, gastroesophageal reflux disease; HTN, hypertension; MDD, major depressive disorder; OR, odds ratio.

^a^ Note that 1398 patients were excluded from this analysis because they did not have follow-up 3 years after diagnosis.

^b^ Statistical significance is defined as *P* < .003.

Time from diagnosis to surgery within 1 year vs surgery beyond 1 year was associated with increased calcium (11.00 vs 10.90 mg/dL; difference, 0.10 mg/dL; 95% CI, 0.06-0.15 mg/dL; *P* < .001) and PTH (135 vs 112 pg/mL; difference, 23 pg/mL; 95% CI, 12-34 pg/mL; *P* < .001) (eTable 4 in Supplement 1). Among the 7856 patients (59.8%) who did not undergo surgery, 1169 (14.9%) were prescribed bisphosphonates, 380 (4.8%) were prescribed cinacalcet, and 177 (2.3%) were prescribed a combination for medical management. The remainder did not have documented treatment.

## Discussion

The reported range of patients with hypercalcemia whose PTH levels are obtained is 23.4% to 33.0%.^13,15,18,19^ Approximately 43% of those with hypercalcemia have PHP.^15^ However, the condition is diagnosed in just 1.3% to 8.0% of patients.^15,18,19^ The findings of this cohort study are consistent with historical data, noting a workup including PTH for approximately 2 of 5 patients. We found that 33.3% of patients with hypercalcemia were at high risk for PHP but did not receive a diagnosis. When added to the 9.7% with a diagnosis, the actual prevalence of PHP in patients with hypercalcemia could be over 43%. Patients in the South had the greatest likelihood of being high-risk and not receiving a diagnosis. More than one-quarter may have not received a diagnosis even in the Midwest, which had the lowest rate of undetected PHP. This is indicative of a problem affecting patients throughout the US.

Prior studies^13,14,15,16,17,18,19^ have reported the underdiagnosis and undertreatment of PHP. Because the disease is often asymptomatic,^30^ whether it had a clinically meaningful impact on patients was unknown. Our study shows that patients at high risk who did not have a diagnosis experienced significantly increased disease sequelae vs matched controls. Compared with patients with a diagnosis, the high-risk group whose PTH levels were obtained had decreased rates of some, no difference in others, and increased rates of other associated symptoms and diagnoses. Differentiating these groups were patients with a diagnosis who were more likely to have classic PHP manifestations, such as osteoporosis and urolithiasis. Symptoms and diagnoses in patients whose PHP went undiagnosed likely contributed to decreased quality of life and increased use of medical services and associated costs. This is especially noteworthy, considering that parathyroidectomy is a cost-effective cure associated with substantial improvement in symptoms.^9,12,31,32,33^

It is unclear why patients with unexplained hypercalcemia did not undergo measurement of PTH. Although this group experienced increased rates of symptoms and diagnoses associated with PHP, their symptoms were considerably less severe compared with those with PTH measurements. It may be that they were considered to be asymptomatic and therefore did not undergo further workup. Diagnostic evaluation should not be restricted to the most symptomatic patients; therefore, this represents an opportunity for improvement. Furthermore, research indicates that patients who are labeled asymptomatic may still report symptoms when prompted or after undergoing cognitive testing.^12^ Our review only reported the rates of those symptoms that were coded, which likely underrepresented the true incidence of symptomatic patients. This suggests that there should be a strong suspicion for hyperparathyroidism in patients with hypercalcemia.^34^

A possible explanation for missed diagnoses in patients with hypercalcemia and inappropriately nonsuppressed PTH between 50 and 65 pg/mL could be they were not identified because their PTH levels were within the reference range. Patients without a diagnosis with frankly elevated PTH greater than or equal to 65 pg/mL may be explained by the fact that clinicians often attribute hypercalcemia to other causes, even in patients with elevated PTH.^35^

Another contributing factor for missed diagnoses may be that high-risk patients had lower calcium and PTH levels vs those whose PHP was diagnosed. Similarly, patients who experienced delayed diagnosis or time to treatment also had lower calcium and PTH compared with the timely diagnosis and treatment groups. Although greater PTH levels increased the likelihood of diagnosis and treatment, they did not correlate with disease burden in our study, a finding consistent with prior literature.^36^

Among patients with a PHP diagnosis, those whose workup took over a year had significantly increased rates of disease sequelae over time, as seen at 2 to 3 years after hypercalcemia. Our study found that 40.2% of patients underwent surgical management after diagnosis, which is within the reported range of 16.8% to 67.2%.^15,17,18,19^ Patients who waited longer than 1 year from diagnosis to surgery also had increased rates of serious complications such as osteoporosis and HTN over time. Therefore, efforts to reduce diagnostic and treatment delays are worthwhile endeavors. Raising awareness about the diagnosis of PHP is important because it will lead to the evaluation of those associated conditions that should prompt referral to surgery as per the American Association of Endocrine Surgeons guidelines.^11^

### Limitations

To our knowledge, this is the largest retrospective study of US patients to date, representing 30% of the population older than 40 years, which increases the generalizability of these results.^37^ However, it is not without limitations. If a patient received some of their workup outside an included HCO, they may be incorrectly categorized as having had an insufficient workup. This is unlikely because the TriNetX Research Network data are obtained from large HCOs with multiple inpatient and outpatient facilities. The analysis is limited by the accuracy of data entry in the electronic medical records. Both shortcomings are minimized by filtering for patients with at least 2 documented levels of hypercalcemia and at least 3 years of follow-up, suggesting established care within an included HCO. Given the variability in calcium reference ranges by as much as 0.5 mg/dL,^22^ this study did not include patients with calcium levels between 10.0 to 10.5 mg/dL, which may have been flagged as elevated by some laboratories. Some patients who were excluded as having another explanation for hypercalcemia may have had concomitant undiagnosed PHP. These limitations and the strict exclusion criteria may have underestimated the true prevalence of undiagnosed PHP. Conversely, our analysis assumed that all patients considered high risk actually had PHP.

## Conclusions

The findings of this cohort study suggest that many patients at high risk for PHP may not have a diagnosis and may be untreated despite experiencing associated symptoms and diagnoses. These findings were widespread across the US. Patients with prolonged time from hypercalcemia to diagnosis and diagnosis to treatment reported increased disease sequelae over time. Therefore, system-level interventions aimed at ensuring proper diagnoses and reducing duration of workup and time to treatment are necessary for improved outcomes.

## Notes

Supplement 1. **eMethods.** Diagnosis (*ICD-10*) and Procedure (*CPT*) Codes **eTable 1.** Associated Diagnoses and Symptoms in Patients in the First (Hypercalcemia and PTH ≥50 pg/mL Without a Documented Diagnosis) (n = 20,176) and Second (Unexplained Hypercalcemia and No Further Workup) (n = 24,905) High-risk Groups for PHP **eTable 2.** Associated Diagnoses and Symptoms in Patients Diagnosed With PHP (n = 13,136) Compared to Matched Controls **eTable 3.** Associated Diagnoses and Symptoms in High-risk Group 1 (Hypercalcemia and PTH ≥50 pg/mL) (n = 20,176) and High-risk Group 2 (Unexplained Hypercalcemia and No Further Workup) (n = 24,905) Compared to Those Diagnosed (n = 13,136) **eTable 4.** Lab Values for Patient Cohorts **eTable 5.** Associated Diagnoses and Symptoms in Patients at High Risk of PHP With Hypercalcemia and PTH ≥40 (n = 23,969) or 50 pg/mL (n = 20,176) Without a Documented Diagnosis of PHP Compared to Matched Controls **eTable 6.** Associated Diagnoses and Symptoms in Patients at High Risk of PHP With Hypercalcemia and PTH ≥65 (n = 14,959) or 100 (n = 7,387) pg/mL Without a Documented Diagnosis of PHP Compared to Matched Controls **eTable 7.** Comparing Diagnoses and Symptoms in Patients at High Risk of PHP With Hypercalcemia and PTH ≥40 (n = 23,969) or 50 pg/mL (n = 20,176) Without a Documented Diagnosis With Those Diagnosed (n = 13,136) **eTable 8.** Comparing Diagnoses and Symptoms in Patients at High Risk of PHP With Hypercalcemia and PTH ≥65 (n = 14,959) or 100 pg/mL (n = 7,387) Without a Documented Diagnosis With Those Diagnosed (n = 13,136) **eTable 9.** Comparing Diagnoses and Symptoms in Patients at High Risk of PHP With Hypercalcemia and PTH ≥40 (n = 23,969) or 50 pg/mL (n = 20,176) Without a Documented Diagnosis With Those Diagnosed With Similar PTH Levels (n = 12,294 for PTH ≥40 and n = 11,968 for ≥50 pg/mL) **eTable 10.** Comparing Diagnoses and Symptoms in Patients at High Risk of PHP With Hypercalcemia and PTH ≥65 (n = 14,959) or 100 pg/mL (n = 7,387) Without a Documented Diagnosis With Those Diagnosed With Similar PTH Levels (n = 11,262 for PTH ≥65 and n = 8,192 for ≥100 pg/mL)

Supplement 2.
Data Sharing Statement
