# Supplementary material for: Analysis of Time to Diagnosis and Outcomes Among Adults With Primary Hyperparathyroidism
Source: JAMA Netw Open. 2022 Dec 27;5(12):e2248332. doi: 10.1001/jamanetworkopen.2022.48332 (PMC9857508; doi:10.1001/jamanetworkopen.2022.48332)
Supplement: Supplement 1. — eMethods. Diagnosis (ICD-10) and Procedure (CPT) Codes eTable 1. Associated Diagnoses and Symptoms in Patients in the First (Hypercalcemia and PTH ≥50 pg/mL Without a Documented Diagnosis) (n = 20,176) and Second (Unexplained Hypercalcemia and No Further Workup) (n = 24,905) High-risk Groups for PHP eTable 2. Associated Diagnoses and Symptoms in Patients Diagnosed With PHP (n = 13,136) Compared to Matched Controls eTable 3. Associated Diagnoses and Symptoms in High-risk Group 1 (Hypercalcemia and PTH ≥50 pg/mL) (n = 20,176) and High-risk Group 2 (Unexplained Hypercalcemia and No Further Workup) (n = 24,905) Compared to Those Diagnosed (n = 13,136) eTable 4. Lab Values for Patient Cohorts eTable 5. Associated Diagnoses and Symptoms in Patients at High Risk of PHP With Hypercalcemia and PTH ≥40 (n = 23,969) or 50 pg/mL (n = 20,176) Without a Documented Diagnosis of PHP Compared to Matched Controls eTable 6. Associated Diagnoses and Symptoms in Patients at High Risk of PHP With Hypercalcemia and PTH ≥65 (n = 14,959) or 100 (n = 7,387) pg/mL Without a Documented Diagnosis of PHP Compared to Matched Controls eTable 7. Comparing Diagnoses and Symptoms in Patients at High Risk of PHP With Hypercalcemia and PTH ≥40 (n = 23,969) or 50 pg/mL (n = 20,176) Without a Documented Diagnosis With Those Diagnosed (n = 13,136) eTable 8. Comparing Diagnoses and Symptoms in Patients at High Risk of PHP With Hypercalcemia and PTH ≥65 (n = 14,959) or 100 pg/mL (n = 7,387) Without a Documented Diagnosis With Those Diagnosed (n = 13,136) eTable 9. Comparing Diagnoses and Symptoms in Patients at High Risk of PHP With Hypercalcemia and PTH ≥40 (n = 23,969) or 50 pg/mL (n = 20,176) Without a Documented Diagnosis With Those Diagnosed With Similar PTH Levels (n = 12,294 for PTH ≥40 and n = 11,968 for ≥50 pg/mL) eTable 10. Comparing Diagnoses and Symptoms in Patients at High Risk of PHP With Hypercalcemia and PTH ≥65 (n = 14,959) or 100 pg/mL (n = 7,387) Without a Documented Diagnosis With Those [file jamanetwopen-e2248332-s001.pdf]

Supplemental Online Content

Lorenz FJ, Beauchamp-Perez F, Manni A, Chung T, Goldenberg D, Goyal N. Analysis of time to diagnosis and outcomes among adults with primary hyperparathyroidism. *JAMA Netw Open*. 2022;5(12):e2248332. doi:10.1001/jamanetworkopen.2022.48332

eMethods. Diagnosis (*ICD-10*) and Procedure (*CPT*) Codes
eTable 1. Associated Diagnoses and Symptoms in Patients in the First (Hypercalcemia and PTH ≥50 pg/mL Without a Documented Diagnosis) (n = 20,176) and Second (Unexplained Hypercalcemia and No Further Workup) (n = 24,905) High-risk Groups for PHP
eTable 2. Associated Diagnoses and Symptoms in Patients Diagnosed With PHP (n = 13,136) Compared to Matched Controls
eTable 3. Associated Diagnoses and Symptoms in High-risk Group 1 (Hypercalcemia and PTH ≥50 pg/mL) (n = 20,176) and High-risk Group 2 (Unexplained Hypercalcemia and No Further Workup) (n = 24,905) Compared to Those Diagnosed (n = 13,136)
eTable 4. Lab Values for Patient Cohorts
eTable 5. Associated Diagnoses and Symptoms in Patients at High Risk of PHP With Hypercalcemia and PTH ≥40 (n = 23,969) or 50 pg/mL (n = 20,176) Without a Documented Diagnosis of PHP Compared to Matched Controls
eTable 6. Associated Diagnoses and Symptoms in Patients at High Risk of PHP With Hypercalcemia and PTH ≥65 (n = 14,959) or 100 (n = 7,387) pg/mL Without a Documented Diagnosis of PHP Compared to Matched Controls
eTable 7. Comparing Diagnoses and Symptoms in Patients at High Risk of PHP With Hypercalcemia and PTH ≥40 (n = 23,969) or 50 pg/mL (n = 20,176) Without a Documented Diagnosis With Those Diagnosed (n = 13,136)
eTable 8. Comparing Diagnoses and Symptoms in Patients at High Risk of PHP With Hypercalcemia and PTH ≥65 (n = 14,959) or 100 pg/mL (n = 7,387) Without a Documented Diagnosis With Those Diagnosed (n = 13,136)
eTable 9. Comparing Diagnoses and Symptoms in Patients at High Risk of PHP With Hypercalcemia and PTH ≥40 (n = 23,969) or 50 pg/mL (n = 20,176) Without a Documented Diagnosis With Those Diagnosed With Similar PTH Levels (n = 12,294 for PTH ≥40 and n = 11,968 for ≥50 pg/mL)
eTable 10. Comparing Diagnoses and Symptoms in Patients at High Risk of PHP With Hypercalcemia and PTH ≥65 (n = 14,959) or 100 pg/mL (n = 7,387) Without a Documented Diagnosis With Those Diagnosed With Similar PTH Levels (n = 11,262 for PTH ≥65 and n = 8,192 for ≥100 pg/mL)

This supplemental material has been provided by the authors to give readers additional information about their work.

© 2022 Lorenz FJ et al. *JAMA Network Open.*

eMethods. Diagnosis (*ICD-10*) and Procedure (*CPT*) Codes

<u>Diagnosis Codes</u>
D35.1 Benign Neoplasm of Parathyroid Gland
E21.0 Primary Hyperparathyroidism
E21.1 Secondary Hyperparathyroidism, Not Elsewhere Classified
N25.81 Secondary Hyperparathyroidism of Renal Origin
E21.2 Other Hyperparathyroidism
E21.4 Other Specified Disorders of Parathyroid Gland
C75.0 Malignant Neoplasm of Parathyroid Gland
E31.2 Multiple Endocrine Neoplasia (MEN) Syndromes
Z94.0 Kidney Transplant Status
N18.4-6 Chronic kidney disease stage 4, 5, or end stage
C73 Malignant Neoplasm of Thyroid Gland
C25 Malignant Neoplasm of Pancreas
C49 Malignant Neoplasm of Connective and Soft Tissue
C56 Malignant Neoplasm of Ovary
C61 Malignant Neoplasm of Prostate
C81-96 Malignant Neoplasms of Lymphoid, Hematopoietic, and Related Tissue
C18 Malignant Neoplasm of Colon
C19 Malignant Neoplasm of Rectosigmoid Junction
C20 Malignant Neoplasm of Rectum
C67 Malignant Neoplasm of Bladder
C50 Malignant Neoplasm of Breast
C74.1 Malignant Neoplasm of Adrenal Gland
A15-A19 Tuberculosis
D86 Sarcoidosis
E22.0 Acromegaly
E27.1 Primary Adrenocortical Insufficiency
M88 Osteitis Deformans
E05 Thyrotoxicosis (Hyperthyroidism)
E67.3 Hypervitaminosis D
E67.0 Hypervitaminosis A
M80, M81, M85.5, M85.9 Osteoporosis/Osteopenia
N20-N23 Urolithiasis
F32, F33 Major Depressive Disorder
R35 Polyuria
K59.0 Constipation
M79.1, M25.5 Myalgias/Joint Pain
R53.1 Weakness
R10.1, R10.3, R10.81, R10.84, R10.9 Abdominal pain
R53.8 Malaise and Fatigue
F41 Anxiety Disorders
G47.0 Insomnia
R41.1, R41.2, R41.3, R41.8, R41.9 Amnesia
K21 Gastroesophageal Reflux Disease
I10 Hypertension
R51 Headache
S12, S22, S32, S42, S52, S62, S72, S82, S92, M80 Fracture
R11.0 Nausea
K80, K81, K82.8 Gallstones

© 2022 Lorenz FJ et al. *JAMA Network Open.*

Procedure Codes
0GBP/0GBN Endocrine System/ Excision/ Inferior Parathyroid Gland, Left/Right
0GBM/0GBL Endocrine System/ Excision/ Superior Parathyroid Gland, Left/Right
0GBQ Endocrine System/ Excision/ Parathyroid Glands, Multiple
0GBR Endocrine System/ Excision Parathyroid Gland
0GTR Endocrine System/ Resection/ Parathyroid Gland
0GTM/0GTL Endocrine System/ Resection/ Superior Parathyroid Gland, Left/Right
0GTQ Endocrine System/ Resection/ Parathyroid Glands, Multiple
0GTP/0GTN Endocrine System/ Resection/ Inferior Parathyroid Gland, Left/Right
60500 Parathyroidectomy or Exploration of Parathyroid(s)
60505 Parathyroidectomy or Exploration of Parathyroid(s); With Mediastinal Exploration, Sternal Split or Transthoracic Approach
3E0636Z/3E0536Z/3E0336Z/3E0436Z Total Parenteral Nutrition

Laboratory Codes
8001 Glomerular Filtration Rate in Serum, Plasma, or Blood
9022 Calcium in Serum, Plasma, or Blood
38230-9/1994-4 Ionized Calcium in Blood
9039 Parathyrin in Serum or Plasma
9045 Albumin in Serum, Plasma, or Blood
9024 Creatinine in Serum, Plasma, or Blood
9034 Calcidiol in Serum or Plasma

Medication Codes
CV701 Thiazides/Related Diuretics
6448 Lithium
214342 Calcium Acetate
1897 Calcium Carbonate
1921069 Abaloparatide
32915 Teriparatide
46041 Alendronate
77655 Zoledronic Acid
115264 Ibandronate
73056 Risedronate
11473 Pamidronate
42682 Etidronate
57230 Tiludronate
407990 Cinacalcet

© 2022 Lorenz FJ et al. *JAMA Network Open.*

eTable 1: Associated Diagnoses and Symptoms in Patients in the First (Hypercalcemia and PTH ≥50 pg/mL Without a Documented Diagnosis) (n = 20,176) and Second (Unexplained Hypercalcemia and No Further Workup) (n = 24,905) High-risk Groups for PHP

	**Diagnosis**	**High-Risk PHP PTH ≥50** (n = 17,853 after matching)	**High-Risk PHP Unexplained Hypercalcemia** (n = 17,853 after matching)	**Odds Ratio (95% CI)**	[a]***P* value**
**0-1 Year**	Osteopenia/Osteoporosis	2,436 (13.6%)	1,206 (6.8%)	2.2 (2.0-2.3)	<0.001
	Fractures	682 (3.8%)	498 (2.8%)	1.4 (1.2-1.6)	<0.001
	Urolithiasis	747 (4.2%)	491 (2.8%)	1.5 (1.4-1.7)	<0.001
	MDD	1,914 (10.7%)	1,744 (9.8%)	1.1 (1.0-1.2)	0.003
	Anxiety Disorders	1,826 (10.2%)	1,684 (9.4%)	1.1 (1.0-1.2)	0.01
	HTN	8,497 (47.6%)	6,430 (36.0%)	1.6 (1.5-1.7)	<0.001
	GERD	2,749 (15.4%)	2,265 (12.7%)	1.3 (1.2-1.3)	<0.001
	Malaise/Fatigue	1,959 (11.0%)	1,494 (8.4%)	1.3 (1.3-1.4)	<0.001
	Joint Pain/Myalgias	5,851 (32.8%)	4,817 (27.0%)	1.3 (1.3-1.4)	<0.001
	Constipation	1,165 (6.5%)	950 (5.3%)	1.2 (1.1-1.4)	<0.001
	Insomnia	837 (4.7%)	638 (3.6%)	1.3 (1.2-1.5)	<0.001
	Polyuria	821 (4.6%)	651 (3.6%)	1.3 (1.1-1.4)	<0.001
	Weakness	1,315 (7.4%)	1,010 (5.7%)	1.3 (1.2-1.4)	<0.001
	Abdominal Pain	2,029 (11.4%)	2,106 (11.8%)	1.0 (0.9-1.0)	0.20
	Headache	1,033 (5.8%)	918 (5.1%)	1.1 (1.0-1.2)	0.01
	Nausea	783 (4.4%)	701 (3.9%)	1.1 (1.0-1.2)	0.03
	Amnesia	687 (3.8%)	747 (4.2%)	0.9 (0.8-1.0)	0.11
	Gallstones	357 (2.0%)	313 (1.8%)	1.1 (1.0-1.3)	0.09
**1-2 Years**	Osteopenia/Osteoporosis	2,510 (14.1%)	1,269 (7.1%)	2.1 (2.0-2.3)	<0.001
	Fractures	608 (3.4%)	421 (2.4%)	1.5 (1.3-1.7)	<0.001
	Urolithiasis	629 (3.5%)	359 (2.0%)	1.8 (1.6-2.0)	<0.001
	MDD	1,832 (10.3%)	1,463 (8.2%)	1.3 (1.2-1.4)	<0.001
	Anxiety Disorders	1,655 (9.3%)	1,397 (7.8%)	1.2 (1.1-1.3)	<0.001
	HTN	8,331 (46.7%)	5,688 (31.9%)	1.9 (1.8-2.0)	<0.001
	GERD	2,518 (14.1%)	1,912 (10.7%)	1.4 (1.3-1.5)	<0.001
	Malaise/Fatigue	1,451 (8.1%)	1,047 (5.9%)	1.4 (1.3-1.5)	<0.001
	Joint Pain/Myalgias	3,111 (17.4%)	2,201 (12.3%)	1.5 (1.4-1.6)	<0.001
	Constipation	930 (5.2%)	677 (3.8%)	1.4 (1.3-1.5)	<0.001
	Insomnia	680 (3.8%)	507 (2.8%)	1.4 (1.2-1.5)	<0.001
	Polyuria	742 (4.2%)	540 (3.0%)	1.4 (1.2-1.6)	<0.001
	Weakness	892 (5.0%)	687 (3.8%)	1.3 (1.2-1.5)	<0.001
	Abdominal Pain	1,584 (8.9%)	1,410 (7.9%)	1.1 (1.1-1.2)	<0.001
	Headache	866 (4.9%)	623 (3.5%)	1.4 (1.3-1.6)	<0.001
	Nausea	555 (3.1%)	424 (2.4%)	1.3 (1.2-1.5)	<0.001
	Amnesia	582 (3.3%)	511 (2.9%)	1.1 (1.0-1.3)	0.03
	Gallstones	249 (1.4%)	183 (1.0%)	1.4 (1.1-1.7)	0.001
**2-3 Years**	Osteopenia/Osteoporosis	2,699 (15.1%)	1,386 (7.8%)	2.1 (2.0-2.3)	<0.001
	Fractures	636 (3.6%)	461 (2.6%)	1.4 (1.2-1.6)	<0.001
	Urolithiasis	622 (3.5%)	351 (2.0%)	1.8 (1.6-2.1)	<0.001
	MDD	1,922 (10.8%)	1,498 (8.4%)	1.3 (1.2-1.4)	<0.001
	Anxiety Disorders	1,743 (9.8%)	1,499 (8.4%)	1.2 (1.1-1.3)	<0.001
	HTN	8,503 (47.6%)	5,855 (32.8%)	1.9 (1.8-1.9)	<0.001
	GERD	2,621 (14.7%)	1,991 (11.2%)	1.4 (1.3-1.5)	<0.001
	Malaise/Fatigue	1,485 (8.3%)	1,055 (5.9%)	1.4 (1.3-1.6)	<0.001
	Joint Pain/Myalgias	3,067 (17.2%)	2,341 (13.1%)	1.4 (1.3-1.5)	<0.001
	Constipation	947 (5.3%)	711 (4.0%)	1.4 (1.2-1.5)	<0.001
	Insomnia	727 (4.1%)	537 (3.0%)	1.4 (1.2-1.5)	<0.001
	Polyuria	804 (4.5%)	519 (2.9%)	1.6 (1.4-1.8)	<0.001
	Weakness	969 (5.4%)	662 (3.7%)	1.5 (1.3-1.6)	<0.001
	Abdominal Pain	1,611 (9.0%)	1,457 (8.2%)	1.1 (1.0-1.2)	0.004
	Headache	790 (4.4%)	626 (3.5%)	1.3 (1.1-1.4)	<0.001
	Nausea	542 (3.0%)	400 (2.2%)	1.4 (1.2-1.6)	<0.001
	Amnesia	621 (3.5%)	554 (3.1%)	1.1 (1.0-1.3)	0.05
	Gallstones	252 (1.4%)	200 (1.1%)	1.3 (1.0-1.5)	0.01
**Entire Study Period (0-3 Years)**	Osteopenia/Osteoporosis	4,073 (22.8%)	2,334 (13.1%)	2.0 (1.9-2.1)	<0.001
	Fractures	1,363 (7.6%)	1,100 (6.2%)	1.3 (1.2-1.4)	<0.001
	Urolithiasis	1,218 (6.8%)	865 (4.8%)	1.4 (1.3-1.6)	<0.001
	MDD	3,063 (17.2%)	2,684 (15.0%)	1.2 (1.1-1.2)	<0.001
	Anxiety Disorders	2,947 (16.5%)	2,661 (14.9%)	1.1 (1.1-1.2)	<0.001
	HTN	10,434 (58.4%)	8,224 (46.1%)	1.6 (1.6-1.7)	<0.001
	GERD	4,306 (24.1%)	3,587 (20.1%)	1.3 (1.2-1.3)	<0.001
	Malaise/Fatigue	3,495 (19.6%)	2,682 (15.0%)	1.4 (1.3-1.5)	<0.001
	Joint Pain/Myalgias	5,851 (32.8%)	4,817 (27.0%)	1.3 (1.3-1.4)	<0.001
	Constipation	2,181 (12.2%)	1,774 (9.9%)	1.3 (1.2-1.3)	<0.001

	Insomnia	1,499 (8.4%)	1,151 (6.4%)	1.3 (1.2-1.4)	<0.001
	Polyuria	1,798 (10.1%)	1,359 (7.6%)	1.4 (1.3-1.5)	<0.001
	Weakness	2,387 (13.4%)	1,897 (10.6%)	1.3 (1.2-1.4)	<0.001
	Abdominal Pain	3,734 (20.9%)	3,624 (20.3%)	1.0 (1.0-1.1)	0.15
	Headache	2,021 (11.3%)	1,711 (9.6%)	1.2 (1.1-1.3)	<0.001
	Nausea	1,433 (8.0%)	1,246 (7.0%)	1.2 (1.1-1.3)	<0.001
	Amnesia	1,369 (7.7%)	1,380 (7.7%)	1.0 (0.9-1.1)	0.83
	Gallstones	671 (3.8%)	582 (3.3%)	1.2 (1.0-1.3)	0.01

Abbreviations: PHP, primary hyperparathyroidism; PTH, parathyroid hormone; MDD, major depressive disorder; HTN, hypertension; GERD, gastroesophageal reflux disease; CI, confidence interval

[a]Statistical significance defined as *P* <0.003

© 2022 Lorenz FJ et al. *JAMA Network Open.*

eTable 2: Associated Diagnoses and Symptoms in Patients Diagnosed With PHP (n = 13,136) Compared to Matched Controls

	**Diagnosis**	**Diagnosed** (n = 13,136 after matching)	**Matched Controls** (n = 13,136 after matching)	**Odds Ratio (95% CI)**	[a]***P* value**
**0-1 Year**	Osteopenia/Osteoporosis	2,839 (21.6%)	849 (6.5%)	4.0 (3.7-4.3)	<0.001
	Fractures	545 (4.1%)	399 (3.0%)	1.4 (1.2-1.6)	<0.001
	Urolithiasis	1,172 (8.9%)	201 (1.5%)	6.3 (5.4-7.3)	<0.001
	MDD	1,657 (12.6%)	1,104 (8.4%)	1.6 (1.5-1.7)	<0.001
	Anxiety Disorders	1,410 (10.7%)	1,121 (8.5%)	1.3 (1.2-1.4)	<0.001
	HTN	6,869 (52.3%)	4,473 (34.1%)	2.1 (2.0-2.2)	<0.001
	GERD	2,415 (18.4%)	1,599 (12.2%)	1.6 (1.5-1.7)	<0.001
	Malaise/Fatigue	1,778 (13.5%)	1,003 (7.6%)	1.9 (1.7-2.1)	<0.001
	Joint Pain/Myalgias	2,791 (21.2%)	1,890 (14.4%)	1.6 (1.5-1.7)	<0.001
	Constipation	1,025 (7.8%)	528 (4.0%)	2.0 (1.8-2.3)	<0.001
	Insomnia	602 (4.6%)	391 (3.0%)	1.6 (1.4-1.8)	<0.001
	Polyuria	791 (6.0%)	406 (3.1%)	2.0 (1.8-2.3)	<0.001
	Weakness	1,228 (9.3%)	679 (5.2%)	1.9 (1.7-2.1)	<0.001
	Abdominal Pain	1,713 (13.0%)	1,190 (9.1%)	1.5 (1.4-1.6)	<0.001
	Headache	927 (7.1%)	609 (4.6%)	1.6 (1.4-1.7)	<0.001
	Nausea	685 (5.2%)	404 (3.1%)	1.7 (1.5-2.0)	<0.001
	Amnesia	615 (4.7%)	356 (2.7%)	1.8 (1.5-2.0)	<0.001
	Gallstones	307 (2.3%)	182 (1.4%)	1.7 (1.4-2.0)	<0.001
**1-2 Years**	Osteopenia/Osteoporosis	2,836 (21.6%)	764 (5.8%)	4.5 (4.1-4.8)	<0.001
	Fractures	463 (3.5%)	278 (2.1%)	1.7 (1.5-2.0)	<0.001
	Urolithiasis	931 (7.1%)	151 (1.1%)	6.6 (5.5-7.8)	<0.001
	MDD	1,522 (11.6%)	863 (6.6%)	1.9 (1.7-2.0)	<0.001
	Anxiety Disorders	1,277 (9.7%)	891 (6.8%)	1.5 (1.4-1.6)	<0.001
	HTN	6,541 (49.8%)	3,625 (27.6%)	2.6 (2.5-2.7)	<0.001
	GERD	2,257 (17.2%)	1,210 (9.2%)	2.0 (1.9-2.2)	<0.001
	Malaise/Fatigue	1,340 (10.2%)	700 (5.3%)	2.0 (1.8-2.2)	<0.001
	Joint Pain/Myalgias	2,573 (19.6%)	1,609 (12.2%)	1.7 (1.6-1.9)	<0.001
	Constipation	816 (6.2%)	330 (2.5%)	2.6 (2.3-2.9)	<0.001
	Insomnia	541 (4.1%)	301 (2.3%)	1.8 (1.6-2.1)	<0.001
	Polyuria	660 (5.0%)	337 (2.6%)	2.0 (1.8-2.3)	<0.001
	Weakness	902 (6.9%)	422 (3.2%)	2.2 (2.0-2.5)	<0.001
	Abdominal Pain	1,438 (10.9%)	755 (5.7%)	2.0 (1.8-2.2)	<0.001
	Headache	707 (5.4%)	371 (2.8%)	2.0 (1.7-2.2)	<0.001
	Nausea	472 (3.6%)	243 (1.8%)	2.0 (1.7-2.3)	<0.001
	Amnesia	486 (3.7%)	209 (1.6%)	2.4 (2.0-2.8)	<0.001
	Gallstones	234 (1.8%)	98 (0.7%)	2.4 (1.9-3.1)	<0.001
**2-3 Years**	Osteopenia/Osteoporosis	3,177 (24.2%)	915 (7.0%)	4.3 (3.9-4.6)	<0.001
	Fractures	496 (3.8%)	296 (2.3%)	1.7 (1.5-2.0)	<0.001
	Urolithiasis	956 (7.3%)	124 (0.9%)	8.2 (6.8-9.9)	<0.001
	MDD	1,561 (11.9%)	886 (6.7%)	1.9 (1.7-2.0)	<0.001
	Anxiety Disorders	1,363 (10.4%)	951 (7.2%)	1.5 (1.4-1.6)	<0.001
	HTN	6,634 (50.5%)	3,833 (29.2%)	2.5 (2.4-2.6)	<0.001
	GERD	2,310 (17.6%)	1,258 (9.6%)	2.0 (1.9-2.2)	<0.001
	Malaise/Fatigue	1,384 (10.5%)	720 (5.5%)	2.0 (1.8-2.2)	<0.001
	Joint Pain/Myalgias	2,619 (19.9%)	1,610 (12.3%)	1.8 (1.7-1.9)	<0.001
	Constipation	776 (5.9%)	365 (2.8%)	2.2 (1.9-2.5)	<0.001
	Insomnia	577 (4.4%)	344 (2.6%)	1.7 (1.5-2.0)	<0.001
	Polyuria	666 (5.1%)	330 (2.5%)	2.1 (1.8-2.4)	<0.001
	Weakness	846 (6.4%)	465 (3.5%)	1.9 (1.7-2.1)	<0.001
	Abdominal Pain	1,374 (10.5%)	778 (5.9%)	1.9 (1.7-2.0)	<0.001
	Headache	723 (5.5%)	378 (2.9%)	2.0 (1.7-2.2)	<0.001
	Nausea	476 (3.6%)	231 (1.8%)	2.1 (1.8-2.5)	<0.001
	Amnesia	506 (3.9%)	274 (2.1%)	1.9 (1.6-2.2)	<0.001
	Gallstones	227 (1.7%)	113 (0.9%)	2.0 (1.6-2.5)	<0.001
**Entire Study Period (0-3 Years)**	Osteopenia/Osteoporosis	4,860 (37.0%)	1,606 (12.2%)	4.2 (4.0-4.5)	<0.001
	Fractures	1,113 (8.5%)	786 (6.0%)	1.5 (1.3-1.6)	<0.001
	Urolithiasis	1,913 (14.6%)	324 (2.5%)	6.7 (6.0-7.6)	<0.001
	MDD	2,677 (20.4%)	1,748 (13.3%)	1.7 (1.6-1.8)	<0.001
	Anxiety Disorders	2,399 (18.3%)	1,803 (13.7%)	1.4 (1.3-1.5)	<0.001
	HTN	8,577 (65.3%)	5,864 (44.6%)	2.3 (2.2-2.5)	<0.001
	GERD	3,913 (29.8%)	2,531 (19.3%)	1.8 (1.7-1.9)	<0.001
	Malaise/Fatigue	3,289 (25.0%)	1,868 (14.2%)	2.0 (1.9-2.1)	<0.001
	Joint Pain/Myalgias	5,085 (38.7%)	3,543 (27.0%)	1.7 (1.6-1.8)	<0.001
	Constipation	1,923 (14.6%)	940 (7.2%)	2.2 (2.0-2.4)	<0.001
	Insomnia	1,150 (8.8%)	728 (5.5%)	1.6 (1.5-1.8)	<0.001
	Polyuria	1,669 (12.7%)	857 (6.5%)	2.1 (1.9-2.3)	<0.001
	Weakness	2,268 (17.3%)	1,267 (9.6%)	2.0 (1.8-2.1)	<0.001

© 2022 Lorenz FJ et al. *JAMA Network Open.*

	Abdominal Pain	3,300 (25.1%)	2,128 (16.2%)	1.7 (1.6-1.8)	<0.001
	Headache	1,794 (13.7%)	1,109 (8.4%)	1.7 (1.6-1.9)	<0.001
	Nausea	1,302 (9.9%)	739 (5.6%)	1.8 (1.7-2.0)	<0.001
	Amnesia	1,159 (8.8%)	664 (5.1%)	1.8 (1.6-2.0)	<0.001
	Gallstones	580 (4.4%)	334 (2.5%)	1.8 (1.5-2.0)	<0.001

Abbreviations: PHP, primary hyperparathyroidism; PTH, parathyroid hormone; MDD, major depressive disorder; HTN, hypertension; GERD, gastroesophageal reflux disease; CI, confidence interval

[a]Statistical significance defined as *P* <0.003

© 2022 Lorenz FJ et al. *JAMA Network Open.*

eTable 3: Associated Diagnoses and Symptoms in High-risk Group 1 (Hypercalcemia and PTH ≥50 pg/mL) (n = 20,176) and High-risk Group 2 (Unexplained Hypercalcemia and No Further Workup) (n = 24,905) Compared to Those Diagnosed (n = 13,136)

	**Diagnosis**	**High-Risk PHP PTH ≥50** (n = 12,842 after matching)	**Diagnosed** (n = 12,842 after matching)	**Odds Ratio (95% CI)**	[a]***P* value**	**High-Risk PHP Unexplained Hypercalcemia** (n = 13,136 after matching)	**Diagnosed** (n = 13,136 after matching)	**Odds Ratio (95% CI)**	[a]***P* value**
**0-1 Year**	Osteopenia/Osteoporosis	1,970 (15.3%)	2,797 (21.8%)	0.7 (0.6-0.7)	<0.001	882 (6.7%)	2,839 (21.6%)	0.3 (0.2-0.3)	<0.001
	Fractures	551 (4.3%)	530 (4.1%)	1.0 (0.9-1.2)	0.51	362 (2.8%)	545 (4.1%)	0.7 (0.6-0.7)	<0.001
	Urolithiasis	620 (4.8%)	1,132 (8.8%)	0.5 (0.5-0.6)	<0.001	374 (2.8%)	1,172 (8.9%)	0.3 (0.3-0.3)	<0.001
	MDD	1,585 (12.3%)	1,606 (12.5%)	1.0 (0.9-1.1)	0.69	1,325 (10.1%)	1,658 (12.6%)	0.8 (0.7-0.8)	<0.001
	Anxiety Disorders	1,549 (12.1%)	1,359 (10.6%)	1.2 (1.1-1.3)	<0.001	1,344 (10.2%)	1,410 (10.7%)	0.9 (0.9-1.0)	0.18
	HTN	6,804 (53.0%)	6,761 (52.6%)	1.0 (1.0-1.1)	0.59	4,892 (37.2%)	6,871 (52.3%)	0.5 (0.5-0.6)	<0.001
	GERD	2,252 (17.5%)	2,364 (18.4%)	0.9 (0.9-1.0)	0.07	1,735 (13.2%)	2,416 (18.4%)	0.7 (0.6-0.7)	<0.001
	Malaise/Fatigue	1,593 (12.4%)	1,735 (13.5%)	0.9 (0.8-1.0)	0.008	1,162 (8.8%)	1,778 (13.5%)	0.6 (0.6-0.7)	<0.001
	Joint Pain/Myalgias	2,631 (20.5%)	2,725 (21.2%)	1.0 (0.9-1.0)	0.15	2,057 (15.7%)	2,791 (21.2%)	0.7 (0.6-0.7)	<0.001
	Constipation	950 (7.4%)	1,005 (7.8%)	0.9 (0.9-1.0)	0.20	726 (5.5%)	1,026 (7.8%)	0.7 (0.6-0.8)	<0.001
	Insomnia	699 (5.4%)	583 (4.5%)	1.2 (1.1-1.4)	<0.001	499 (3.8%)	602 (4.6%)	0.8 (0.7-0.9)	0.002
	Polyuria	639 (5.0%)	773 (6.0%)	0.8 (0.7-0.9)	<0.001	486 (3.7%)	791 (6.0%)	0.6 (0.5-0.7)	<0.001
	Weakness	1,068 (8.3%)	1,195 (9.3%)	0.9 (0.8-1.0)	0.005	794 (6.0%)	1,228 (9.3%)	0.6 (0.6-0.7)	<0.001
	Abdominal Pain	1,683 (13.1%)	1,661 (12.9%)	1.0 (0.9-1.1)	0.68	1,603 (12.2%)	1,713 (13.0%)	0.9 (0.9-1.0)	0.04
	Headache	856 (6.7%)	897 (7.0%)	1.0 (0.9-1.0)	0.31	714 (5.4%)	927 (7.1%)	0.8 (0.7-0.8)	<0.001
	Nausea	672 (5.2%)	660 (5.1%)	1.0 (0.9-1.1)	0.74	547 (4.2%)	685 (5.2%)	0.8 (0.7-0.9)	<0.001
	Amnesia	557 (4.3%)	609 (4.7%)	0.9 (0.8-1.0)	0.12	512 (3.9%)	615 (4.7%)	0.8 (0.7-0.9)	0.002
	Gallstones	293 (2.3%)	301 (2.3%)	1.0 (0.8-1.1)	0.74	255 (1.9%)	307 (2.3%)	0.8 (0.7-1.0)	0.03
**1-2 Years**	Osteopenia/Osteoporosis	2,059 (16.0%)	2,795 (21.8%)	0.7 (0.6-0.7)	<0.001	927 (7.1%)	2,836 (21.6%)	0.3 (0.3-0.3)	<0.001
	Fractures	479 (3.7%)	453 (3.5%)	1.1 (0.9-1.2)	0.39	305 (2.3%)	463 (3.5%)	0.7 (0.6-0.8)	<0.001
	Urolithiasis	518 (4.0%)	903 (7.0%)	0.6 (0.5-0.6)	<0.001	283 (2.2%)	931 (7.1%)	0.3 (0.3-0.3)	<0.001
	MDD	1,511 (11.8%)	1,476 (11.5%)	1.0 (1.0-1.1)	0.50	1,120 (8.5%)	1,522 (11.6%)	0.7 (0.7-0.8)	<0.001
	Anxiety Disorders	1,390 (10.8%)	1,236 (9.6%)	1.1 (1.1-1.2)	0.002	1,122 (8.5%)	1,277 (9.7%)	0.9 (0.8-0.9)	<0.001
	HTN	6,679 (52.0%)	6,446 (50.2%)	1.1 (1.0-1.1)	0.004	4,345 (33.1%)	6,541 (49.8%)	0.5 (0.5-0.5)	<0.001
	GERD	2,058 (16.0%)	2,211 (17.2%)	0.9 (0.9-1.0)	0.01	1,493 (11.4%)	2,257 (17.2%)	0.6 (0.6-0.7)	<0.001
	Malaise/Fatigue	1,206 (9.4%)	1,306 (10.2%)	0.9 (0.8-1.0)	0.04	813 (6.2%)	1,340 (10.2%)	0.6 (0.5-0.6)	<0.001
	Joint Pain/Myalgias	2,548 (19.8%)	2,506 (19.5%)	1.0 (1.0-1.1)	0.51	1,759 (13.4%)	2,573 (19.6%)	0.6 (0.6-0.7)	<0.001
	Constipation	747 (5.8%)	799 (6.2%)	0.9 (0.8-1.0)	0.17	481 (3.7%)	816 (6.2%)	0.6 (0.5-0.6)	<0.001
	Insomnia	558 (4.3%)	527 (4.1%)	1.1 (0.9-1.2)	0.34	381 (2.9%)	541 (4.1%)	0.7 (0.6-0.8)	<0.001
	Polyuria	593 (4.6%)	643 (5.0%)	0.9 (0.8-1.0)	0.14	374 (2.8%)	660 (5.0%)	0.6 (0.5-0.6)	<0.001
	Weakness	732 (5.7%)	880 (6.9%)	0.8 (0.7-0.9)	<0.001	526 (4.0%)	902 (6.9%)	0.6 (0.5-0.6)	<0.001
	Abdominal Pain	1,290 (10.0%)	1,394 (10.9%)	0.9 (0.8-1.0)	0.03	1,060 (8.1%)	1,438 (10.9%)	0.7 (0.7-0.8)	<0.001
	Headache	718 (5.6%)	676 (5.3%)	1.1 (1.0-1.2)	0.25	484 (3.7%)	707 (5.4%)	0.7 (0.6-0.8)	<0.001
	Nausea	467 (3.6%)	458 (3.6%)	1.0 (0.9-1.2)	0.76	342 (2.6%)	472 (3.6%)	0.7 (0.6-0.8)	<0.001
	Amnesia	458 (3.6%)	476 (3.7%)	1.0 (0.8-1.1)	0.55	346 (2.6%)	486 (3.7%)	0.7 (0.6-0.8)	<0.001
	Gallstones	201 (1.6%)	229 (1.8%)	0.9 (0.7-1.1)	0.17	146 (1.1%)	234 (1.8%)	0.6 (0.5-0.8)	<0.001
**2-3 Years**	Osteopenia/Osteoporosis	2,198 (17.1%)	3,132 (24.4%)	0.6 (0.6-0.7)	<0.001	1,047 (8.0%)	3,177 (24.2%)	0.3 (0.3-0.3)	<0.001
	Fractures	506 (3.9%)	485 (3.8%)	1.0 (0.9-1.2)	0.50	356 (2.7%)	496 (3.8%)	0.7 (0.6-0.8)	<0.001
	Urolithiasis	525 (4.1%)	923 (7.2%)	0.6 (0.5-0.6)	<0.001	282 (2.1%)	956 (7.3%)	0.3 (0.2-0.3)	<0.001
	MDD	1,583 (12.3%)	1,509 (11.8%)	1.1 (1.0-1.1)	0.16	1,132 (8.6%)	1,561 (11.9%)	0.7 (0.6-0.8)	<0.001
	Anxiety Disorders	1,462 (11.4%)	1,314 (10.2%)	1.1 (1.0-1.2)	0.003	1,183 (9.0%)	1,363 (10.4%)	0.9 (0.8-0.9)	<0.001
	HTN	6,796 (52.9%)	6,538 (50.9%)	1.1 (1.0-1.1)	0.001	4,496 (34.2%)	6,634 (50.5%)	0.5 (0.5-0.5)	<0.001
	GERD	2,135 (16.6%)	2,250 (17.5%)	0.9 (0.9-1.0)	0.06	1,553 (11.8%)	2,310 (17.6%)	0.6 (0.6-0.7)	<0.001
	Malaise/Fatigue	1,234 (9.6%)	1,356 (10.6%)	0.9 (0.8-1.0)	0.01	833 (6.3%)	1,384 (10.5%)	0.6 (0.5-0.6)	<0.001
	Joint Pain/Myalgias	2,508 (19.5%)	2,560 (19.9%)	1.0 (0.9-1.0)	0.41	1,824 (13.9%)	2,619 (19.9%)	0.6 (0.6-0.7)	<0.001
	Constipation	775 (6.0%)	762 (5.9%)	1.0 (0.9-1.1)	0.73	546 (4.2%)	776 (5.9%)	0.7 (0.6-0.8)	<0.001

© 2022 Lorenz FJ et al. *JAMA Network Open.*

	Insomnia	598 (4.7%)	558 (4.3%)	1.1 (1.0-1.2)	0.23	381 (2.9%)	577 (4.4%)	0.7 (0.6-0.7)	<0.001
	Polyuria	628 (4.9%)	656 (5.1%)	1.0 (0.9-1.1)	0.42	383 (2.9%)	666 (5.1%)	0.6 (0.5-0.6)	<0.001
	Weakness	802 (6.2%)	828 (6.4%)	1.0 (0.9-1.1)	0.51	519 (4.0%)	846 (6.4%)	0.6 (0.5-0.7)	<0.001
	Abdominal Pain	1,333 (10.4%)	1,334 (10.4%)	1.0 (0.9-1.1)	0.98	1,126 (8.6%)	1,374 (10.5%)	0.8 (0.7-0.9)	<0.001
	Headache	666 (5.2%)	698 (5.4%)	1.0 (0.9-1.1)	0.37	484 (3.7%)	723 (5.5%)	0.7 (0.6-0.7)	<0.001
	Nausea	467 (3.6%)	462 (3.6%)	1.0 (0.9-1.2)	0.87	332 (2.5%)	476 (3.6%)	0.7 (0.6-0.8)	<0.001
	Amnesia	488 (3.8%)	498 (3.9%)	1.0 (0.9-1.1)	0.75	408 (3.1%)	506 (3.9%)	0.8 (0.7-0.9)	<0.001
	Gallstones	211 (1.6%)	224 (1.7%)	0.9 (0.8-1.1)	0.53	158 (1.2%)	227 (1.7%)	0.7 (0.6-0.8)	<0.001
**Entire Study Period (0-3 Years)**	Osteopenia/Osteoporosis	3,326 (25.9%)	4,787 (37.3%)	0.6 (0.6-0.6)	<0.001	1,746 (13.3%)	4,860 (37.0%)	0.3 (0.2-0.3)	<0.001
	Fractures	1,091 (8.5%)	1,089 (8.5%)	1.0 (0.9-1.1)	0.96	813 (6.2%)	1,113 (8.5%)	0.7 (0.6-0.8)	<0.001
	Urolithiasis	1,010 (7.9%)	1,847 (14.4%)	0.5 (0.5-0.6)	<0.001	676 (5.1%)	1,913 (14.6%)	0.3 (0.3-0.3)	<0.001
	MDD	2,528 (19.7%)	2,599 (20.2%)	1.0 (0.9-1.0)	0.27	2,060 (15.7%)	2,678 (20.4%)	0.7 (0.7-0.8)	<0.001
	Anxiety Disorders	2,485 (19.4%)	2,320 (18.1%)	1.1 (1.0-1.2)	0.008	2,108 (16.0%)	2,399 (18.3%)	0.9 (0.8-0.9)	<0.001
	HTN	8,371 (65.2%)	8,425 (65.6%)	1.0 (0.9-1.0)	0.48	6,261 (47.7%)	8,579 (65.3%)	0.5 (0.5-0.5)	<0.001
	GERD	3,515 (27.4%)	3,823 (29.8%)	0.9 (0.8-0.9)	<0.001	2,751 (20.9%)	3,914 (29.8%)	0.6 (0.6-0.7)	<0.001
	Malaise/Fatigue	2,859 (22.3%)	3,208 (25.0%)	0.9 (0.8-0.9)	<0.001	2,112 (16.1%)	3,289 (25.0%)	0.6 (0.5-0.6)	<0.001
	Joint Pain/Myalgias	4,771 (37.2%)	4,966 (38.7%)	0.9 (0.9-1.0)	0.01	3,783 (28.8%)	5,085 (38.7%)	0.6 (0.6-0.7)	<0.001
	Constipation	1,779 (13.9%)	1,887 (14.7%)	0.9 (0.9-1.0)	0.05	1,345 (10.2%)	1,924 (14.6%)	0.7 (0.6-0.7)	<0.001
	Insomnia	1,236 (9.6%)	1,116 (8.7%)	1.1 (1.0-1.2)	0.009	852 (6.5%)	1,150 (8.8%)	0.7 (0.7-0.8)	<0.001
	Polyuria	1,432 (11.2%)	1,634 (12.7%)	0.9 (0.8-0.9)	<0.001	1,009 (7.7%)	1,669 (12.7%)	0.6 (0.5-0.6)	<0.001
	Weakness	1,954 (15.2%)	2,212 (17.2%)	0.9 (0.8-0.9)	<0.001	1,482 (11.3%)	2,268 (17.3%)	0.6 (0.6-0.7)	<0.001
	Abdominal Pain	3,075 (23.9%)	3,203 (24.9%)	0.9 (0.9-1.0)	0.07	2,801 (21.3%)	3,300 (25.1%)	0.8 (0.8-0.9)	<0.001
	Headache	1,685 (13.1%)	1,737 (13.5%)	1.0 (0.9-1.0)	0.34	1,343 (10.2%)	1,794 (13.7%)	0.7 (0.7-0.8)	<0.001
	Nausea	1,214 (9.5%)	1,262 (9.8%)	1.0 (0.9-1.0)	0.31	981 (7.5%)	1,302 (9.9%)	0.7 (0.7-0.8)	<0.001
	Amnesia	1,104 (8.6%)	1,140 (8.9%)	1.0 (0.9-1.1)	0.43	965 (7.3%)	1,159 (8.8%)	0.8 (0.7-0.9)	<0.001
	Gallstones	550 (4.3%)	571 (4.4%)	1.0 (0.9-1.1)	0.52	474 (3.6%)	580 (4.4%)	0.8 (0.7-0.9)	<0.001

Abbreviations: PHP, primary hyperparathyroidism; PTH, parathyroid hormone; MDD, major depressive disorder; HTN, hypertension; GERD, gastroesophageal reflux disease; CI, confidence interval

[a]Statistical significance defined as *P* <0.003

© 2022 Lorenz FJ et al. *JAMA Network Open.*

eTable 4: Lab Values for Patient Cohorts

**Patient Cohort**	**Mean Calcium +/- SD** (mg/dL)	**Mean PTH +/- SD** (pg/mL)	**Mean Albumin +/- SD** (g/dL)	[a]**Corrected Calcium** (mg/dL)
High Risk PHP PTH ≥40 (n = 23,969)	10.8 +/- 0.5	84 +/- 67	4.2 +/- 0.4	10.6
High Risk PHP PTH ≥50 (n = 20,176)	10.8 +/- 0.5	93 +/- 70	4.2 +/- 0.4	10.6
High Risk PHP PTH ≥65 (n = 14,959)	10.8 +/- 0.5	108 +/- 77	4.2 +/- 0.4	10.6
High Risk PHP PTH ≥100 (n = 7,387)	10.9 +/- 0.6	148 +/- 100	4.1 +/- 0.4	10.8
Unexplained hypercalcemia and no further workup (n = 24,905)	10.7 +/- 0.5	-	4.2 +/- 0.5	10.5
All PHP Diagnosed (n = 13,136)	10.9 +/- 0.5	107 +/- 101	4.2 +/- 0.6	10.7
PHP Diagnosed PTH ≥40 (n = 12,294)	10.9 +/- 0.5	115 +/- 100	4.2 +/- 0.6	10.7
PHP Diagnosed PTH ≥50 (n = 11,968)	10.9 +/- 0.5	118 +/- 100	4.2 +/- 0.6	10.7
PHP Diagnosed PTH ≥65 (n = 11,262)	10.9 +/- 0.5	125 +/- 103	4.2 +/- 0.6	10.7
PHP Diagnosed PTH ≥100 (n = 8,192)	10.9 +/- 0.6	152 +/- 116	4.2 +/- 0.6	10.7
Hypercalcemia to Diagnosis ≤ 1 Year (n = 3,686)	11.1 +/- 0.7	121 +/- 112	4.2 +/- 0.6	10.9
Hypercalcemia to Diagnosis > 1 Year (n = 9,450)	10.8 +/- 0.4	102 +/- 96	4.2 +/- 0.6	10.6
Diagnosis to Surgery ≤ 1 Year (n = 3,134)	11.0 +/- 0.6	135 +/- 154	4.3 +/- 0.6	10.8
Diagnosis to Surgery > 1 Year (n = 748)	10.9 +/- 0.4	112 +/- 72	4.2 +/- 0.6	10.7

[a]Corrected calcium = serum calcium + 0.8 (4 – serum albumin)

Abbreviations: PHP, primary hyperparathyroidism; PTH, parathyroid hormone; SD, standard deviation

© 2022 Lorenz FJ et al. *JAMA Network Open.*

eTable 5: Associated Diagnoses and Symptoms in Patients at High Risk of PHP With Hypercalcemia and PTH ≥40 (n = 23,969) or 50 pg/mL (n = 20,176) Without a Documented Diagnosis of PHP Compared to Matched Controls

	**Diagnosis**	**High-Risk PHP PTH ≥40** (n = 23,968 after matching)	**Matched Controls** (n = 23,968 after matching)	**Odds Ratio (95% CI)**	[a]***P* value**	**High-Risk PHP PTH ≥50** (n = 20,175 after matching)	**Matched Controls** (n = 20,175 after matching)	**Odds Ratio (95% CI)**	[a]***P* value**
**0-1 Year**	Osteopenia/Osteoporosis	3,291 (13.7%)	1,439 (6.0%)	2.5 (2.3-2.7)	< 0.001	2,662 (13.2%)	1,099 (5.4%)	2.6 (2.5-2.8)	< 0.001
	Fractures	904 (3.8%)	619 (2.6%)	1.5 (1.3-1.4)	< 0.001	719 (3.6%)	478 (2.4%)	1.5 (1.4-1.7)	< 0.001
	Urolithiasis	977 (4.1%)	343 (1.4%)	2.9 (2.6-3.3)	< 0.001	771 (3.8%)	281 (1.4%)	2.8 (2.5-3.2)	< 0.001
	MDD	2,478 (10.3%)	1,677 (7.0%)	1.5 (1.4-1.6)	< 0.001	2,009 (10.0%)	1,393 (6.9%)	1.5 (1.4-1.6)	< 0.001
	Anxiety Disorders	2,368 (9.9%)	1,632 (6.8%)	1.5 (1.4-1.6)	< 0.001	1,920 (9.5%)	1,347 (6.7%)	1.5 (1.4-1.6)	< 0.001
	HTN	11,179 (46.6%)	7,494 (31.3%)	1.9 (1.9-2.0)	< 0.001	9,107 (45.1%)	6,231 (30.9%)	1.8 (1.8-1.9)	< 0.001
	GERD	3,610 (15.1%)	2,591 (10.8%)	1.5 (1.4-1.5)	< 0.001	2,952 (14.6%)	2,047 (10.1%)	1.5 (1.4-1.6)	< 0.001
	Malaise/Fatigue	2,547 (10.6%)	1,640 (6.8%)	1.6 (1.5-1.7)	< 0.001	2,065 (10.2%)	1,328 (6.6%)	1.6 (1.5-1.3)	< 0.001
	Joint Pain/ Myalgias	4,326 (18.0%)	2,947 (12.3%)	1.6 (1.5-1.7)	< 0.001	3,461 (17.2%)	2,385 (11.8%)	1.5 (1.5-1.6)	< 0.001
	Constipation	1,533 (6.4%)	847 (3.5%)	1.9 (1.7-2.0)	< 0.001	1,247 (6.2%)	719 (3.6%)	1.8 (1.6-2.0)	< 0.001
	Insomnia	1,114 (4.6%)	672 (2.8%)	1.7 (1.5-1.9)	< 0.001	885 (4.4%)	524 (2.6%)	1.7 (1.5-1.9)	< 0.001
	Polyuria	1,099 (4.6%)	619 (2.6%)	1.8 (1.6-2.0)	< 0.001	874 (4.3%)	506 (2.5%)	1.8 (1.6-2.0)	< 0.001
	Weakness	1,719 (7.2%)	1,118 (4.7%)	1.6 (1.5-1.7)	< 0.001	1,374 (6.8%)	872 (4.3%)	1.6 (1.5-1.8)	< 0.001
	Abdominal Pain	2,658 (11.1%)	1,950 (8.1%)	1.4 (1.3-1.5)	< 0.001	2,142 (10.6%)	1,573 (7.8%)	1.4 (1.3-1.5)	< 0.001
	Headache	1,407 (5.9%)	966 (4.0%)	1.5 (1.4-1.6)	< 0.001	1,104 (5.5%)	794 (3.9%)	1.4 (1.3-1.6)	< 0.001
	Nausea	1,049 (4.4%)	611 (2.5%)	1.8 (1.6-1.9)	< 0.001	814 (4.0%)	498 (2.5%)	1.7 (1.5-1.9)	< 0.001
	Amnesia	910 (3.8%)	585 (2.4%)	1.6 (1.4-1.8)	< 0.001	738 (3.7%)	506 (2.5%)	1.5 (1.3-1.7)	< 0.001
	Gallstones	462 (1.9%)	320 (1.3%)	1.5 (1.3-1.7)	< 0.001	373 (1.8%)	217 (1.1%)	1.7 (1.5-2.1)	< 0.001
**1-2 Years**	Osteopenia/Osteoporosis	3,387 (14.1%)	1,365 (5.7%)	2.7 (2.6-2.9)	< 0.001	2,718 (13.5%)	1,070 (5.3%)	2.8 (2.6-3.0)	< 0.001
	Fractures	844 (3.5%)	446 (1.9%)	1.9 (1.7-2.2)	< 0.001	647 (3.2%)	367 (1.8%)	1.8 (1.6-2.0)	< 0.001
	Urolithiasis	827 (3.5%)	220 (0.9%)	3.9 (3.3-4.5)	< 0.001	651 (3.2%)	198 (1.0%)	3.4 (2.9-3.9)	< 0.001
	MDD	2,329 (9.7%)	1,395 (5.8%)	1.7 (1.6-1.9)	< 0.001	1,909 (9.5%)	1,164 (5.8%)	1.7 (1.6-1.8)	< 0.001
	Anxiety Disorders	2,164 (9.0%)	1,249 (5.2%)	1.8 (1.7-1.9)	< 0.001	1,740 (8.6%)	1,105 (5.5%)	1.6 (1.5-1.8)	< 0.001
	HTN	10,967 (45.8%)	6,132 (25.6%)	2.5 (2.4-2.6)	< 0.001	8,940 (44.3%)	5,057 (25.1%)	2.4 (2.3-2.5)	< 0.001
	GERD	3,331 (13.9%)	1,991 (8.3%)	1.8 (1.7-1.9)	< 0.001	2,690 (13.3%)	1,606 (8.0%)	1.8 (1.7-1.9)	< 0.001
	Malaise/Fatigue	1,912 (8.0%)	1,118 (4.7%)	1.8 (1.6-1.9)	< 0.001	1,531 (7.6%)	898 (4.5%)	1.8 (1.6-1.9)	< 0.001
	Joint Pain/Myalgias	4,113 (17.2%)	2,502 (10.4%)	1.8 (1.7-1.9)	< 0.001	3,327 (16.5%)	2,004 (9.9%)	1.8 (1.7-1.9)	< 0.001
	Constipation	1,238 (5.2%)	584 (2.4%)	2.2 (2.0-2.4)	< 0.001	1,001 (5.0%)	472 (2.3%)	2.2 (2.0-2.4)	< 0.001
	Insomnia	917 (3.8%)	508 (2.1%)	1.8 (1.6-2.1)	< 0.001	725 (3.6%)	395 (2.0%)	1.9 (1.6-2.1)	< 0.001
	Polyuria	968 (4.0%)	547 (2.3%)	1.8 (1.6-2.0)	< 0.001	786 (3.9%)	420 (2.1%)	1.9 (1.7-2.2)	< 0.001
	Weakness	1,204 (5.0%)	703 (2.9%)	1.6 (1.6-1.9)	< 0.001	948 (4.7%)	547 (2.8%)	1.8 (1.6-2.0)	< 0.001
	Abdominal Pain	2,114 (8.8%)	1,225 (5.1%)	1.8 (1.7-1.9)	< 0.001	1,698 (8.4%)	949 (4.7%)	1.9 (1.7-2.0)	< 0.001
	Headache	1,183 (4.9%)	645 (2.7%)	1.9 (1.7-2.1)	< 0.001	923 (4.6%)	505 (2.5%)	1.9 (1.7-2.1)	< 0.001
	Nausea	765 (3.2%)	372 (1.6%)	2.1 (1.8-2.4)	< 0.001	598 (3.0%)	292 (1.4%)	2.1 (1.8-2.4)	< 0.001
	Amnesia	785 (3.3%)	436 (1.8%)	1.8 (1.6-2.1)	< 0.001	635 (3.1%)	373 (1.8%)	1.7 (1.5-2.0)	< 0.001
	Gallstones	323 (1.3%)	157 (0.7%)	2.1 (1.7-2.5)	< 0.001	265 (1.3%)	124 (0.6%)	2.2 (1.7-2.7)	< 0.001
**2-3 Years**	Osteopenia/Osteoporosis	3,631 (15.1%)	1,510 (6.3%)	2.7 (2.5-2.8)	< 0.001	2,945 (14.6%)	1,254 (6.2%)	2.6 (2.4-2.8)	< 0.001
	Fractures	878 (3.7%)	499 (2.1%)	1.8 (1.6-2.0)	< 0.001	679 (3.4%)	418 (2.1%)	1.6 (1.5-1.9)	< 0.001
	Urolithiasis	834 (3.5%)	212 (0.9%)	4.0 (3.5-4.7)	< 0.001	644 (3.2%)	175 (0.9%)	3.8 (3.2-4.5)	< 0.001
	MDD	2,482 (10.4%)	1,461 (6.1%)	1.8 (1.7-1.9)	< 0.001	2,024 (10.0%)	1,200 (5.9%)	1.8 (1.6-1.9)	< 0.001
	Anxiety Disorders	2,253 (9.4%)	1,419 (5.9%)	1.6 (1.5-1.8)	< 0.001	1,835 (9.1%)	1,163 (5.8%)	1.6 (1.5-1.8)	< 0.001
	HTN	11,167 (46.6%)	6,417 (26.8%)	2.4 (2.3-2.5)	< 0.001	9,124 (45.2%)	5,346 (26.5%)	2.3 (2.2-2.4)	< 0.001
	GERD	3,441 (14.4%)	2,140 (8.9%)	1.7 (1.6-1.8)	< 0.001	2,800 (13.9%)	1,760 (8.7%)	1.7 (1.6-1.8)	< 0.001
	Malaise/Fatigue	1,972 (8.2%)	1,122 (4.7%)	1.8 (1.7-2.0)	< 0.001	1,574 (7.8%)	917 (4.5%)	1.8 (1.6-1.9)	< 0.001
	Joint Pain/Myalgias	4,104 (17.1%)	2,555 (10.7%)	1.7 (1.6-1.8)	< 0.001	3,285 (16.3%)	2,082 (10.3%)	1.7 (1.6-1.8)	< 0.001
	Constipation	1,265 (5.3%)	612 (2.6%)	2.1 (1.9-2.3)	< 0.001	1,017 (5.0%)	511 (2.5%)	2.0 (1.8-2.3)	< 0.001
	Insomnia	963 (4.0%)	522 (2.2%)	1.9 (1.7-2.1)	< 0.001	774 (3.8%)	436 (2.2%)	1.8 (1.6-2.0)	< 0.001

© 2022 Lorenz FJ et al. *JAMA Network Open.*

	Polyuria	1,036 (4.3%)	599 (2.5%)	1.8 (1.6-2.0)	< 0.001	849 (4.2%)	511 (2.5%)	1.7 (1.5-1.9)	< 0.001
	Weakness	1,306 (5.4%)	683 (2.9%)	2.0 (1.8-2.2)	< 0.001	1,034 (5.1%)	577 (2.9%)	1.8 (1.7-2.0)	< 0.001
	Abdominal Pain	2,146 (9.0%)	1,241 (5.2%)	1.8 (1.7-1.9)	< 0.001	1,725 (8.6%)	1,041 (5.2%)	1.7 (1.6-1.9)	< 0.001
	Headache	1,089 (4.5%)	586 (2.4%)	1.9 (1.7-2.1)	< 0.001	848 (4.2%)	480 (2.4%)	1.8 (1.6-2.0)	< 0.001
	Nausea	769 (3.2%)	350 (1.5%)	2.2 (2.0-2.5)	< 0.001	576 (2.9%)	289 (1.3%)	2.0 (1.8-2.3)	< 0.001
	Amnesia	863 (3.6%)	493 (2.1%)	1.8 (1.6-2.0)	< 0.001	681 (3.4%)	404 (2.0%)	1.7 (1.5-1.9)	< 0.001
	Gallstones	344 (1.4%)	155 (0.6%)	2.2 (1.8-2.7)	< 0.001	267 (1.3%)	132 (0.7%)	2.0 (1.7-2.5)	< 0.001
**Entire Study Period (0-3 Years)**	Osteopenia/Osteoporosis	5,449 (22.7%)	2,661 (11.1%)	2.4 (2.2-2.5)	< 0.001	4,447 (22.0%)	2,221 (11.0%)	2.3 (2.2-2.4)	< 0.001
	Fractures	1,836 (7.7%)	1,254 (5.2%)	1.5 (1.4-1.6)	< 0.001	1,450 (7.2%)	1,044 (5.2%)	1.4 (1.3-1.5)	< 0.001
	Urolithiasis	1,600 (6.7%)	569 (2.4%)	2.9 (2.7-3.2)	< 0.001	1,262 (6.3%)	470 (2.3%)	2.8 (2.5-3.1)	< 0.001
	MDD	3,962 (16.5%)	2,751 (11.5%)	1.5 (1.4-1.6)	< 0.001	3,218 (16.0%)	2,253 (11.2%)	1.5 (1.4-1.6)	< 0.001
	Anxiety Disorders	3,824 (16.0%)	2,672 (11.1%)	1.5 (1.4-1.6)	< 0.001	3,097 (15.4%)	2,177 (10.8%)	1.5 (1.4-1.6)	< 0.001
	HTN	13,639 (56.9%)	9,749 (40.7%)	1.9 (1.9-2.0)	< 0.001	11,187 (55.5%)	8,114 (40.2%)	1.9 (1.8-1.9)	< 0.001
	GERD	5,619 (23.4%)	4,151 (17.3%)	1.5 (1.4-1.5)	< 0.001	4,602 (22.8%)	3,430 (17.0%)	1.4 (1.4-1.5)	< 0.001
	Malaise/Fatigue	4,565 (19.0%)	2,947 (12.3%)	1.7 (1.6-1.8)	< 0.001	3,687 (18.3%)	2,428 (12.0%)	1.6 (1.5-1.7)	< 0.001
	Joint Pain/Myalgias	7,759 (32.4%)	5,589 (23.3%)	1.6 (1.5-1.6)	< 0.001	6,266 (31.1%)	4,591 (22.8%)	1.5 (1.5-1.6)	< 0.001
	Constipation	2,874 (12.0%)	1,577 (6.6%)	1.9 (1.8-2.1)	< 0.001	2,335 (11.6%)	1,316 (6.5%)	1.9 (1.7-2.0)	< 0.001
	Insomnia	1,999 (8.3%)	1,193 (5.0%)	1.7 (1.6-1.9)	< 0.001	1,592 (7.9%)	994 (4.9%)	1.7 (1.5-1.8)	< 0.001
	Polyuria	2,371 (9.9%)	1,427 (6.0%)	1.7 (1.6-1.9)	< 0.001	1,905 (9.4%)	1,205 (6.0%)	1.6 (1.5-1.8)	< 0.001
	Weakness	3,145 (13.1%)	1,992 (8.3%)	1.7 (1.6-1.8)	< 0.001	2,522 (12.5%)	1,670 (8.3%)	1.6 (1.5-1.7)	< 0.001
	Abdominal Pain	4,906 (20.5%)	3,417 (14.3%)	1.5 (1.5-1.6)	< 0.001	3,979 (19.7%)	2,844 (14.1%)	1.5 (1.4-1.6)	< 0.001
	Headache	2,719 (11.3%)	1,772 (7.4%)	1.6 (1.5-1.7)	< 0.001	2,155 (10.7%)	1,453 (7.2%)	1.5 (1.4-1.7)	< 0.001
	Nausea	1,941 (8.1%)	1,115 (4.7%)	1.8 (1.7-1.9)	< 0.001	1,518 (7.5%)	919 (4.6%)	1.7 (1.6-1.9)	< 0.001
	Amnesia	1,852 (7.7%)	1,173 (4.9%)	1.6 (1.5-1.8)	< 0.001	1,484 (7.4%)	965 (4.8%)	1.6 (1.5-1.7)	< 0.001
	Gallstones	883 (3.7%)	532 (2.2%)	1.7 (1.5-1.9)	< 0.001	708 (3.5%)	442 (2.2%)	1.6 (1.4-1.8)	< 0.001

Abbreviations: PHP, primary hyperparathyroidism; PTH, parathyroid hormone; MDD, major depressive disorder; HTN, hypertension; GERD, gastroesophageal reflux disease; CI, confidence interval

[a]Statistical significance defined as *P* <0.003

© 2022 Lorenz FJ et al. *JAMA Network Open.*

eTable 6: Associated Diagnoses and Symptoms in Patients at High Risk of PHP With Hypercalcemia and PTH ≥65 (n = 14,959) or 100 (n = 7,387) pg/mL Without a Documented Diagnosis of PHP Compared to Matched Controls

	**Diagnosis**	**High-Risk PHP PTH ≥65** (n =14,959 after matching)	**Matched Controls** (n = 14,959 after matching)	**Odds Ratio (95% CI)**	[a]***P* value**	**High-Risk PHP PTH ≥100** (n = 7,386 after matching)	**Matched Controls** (n = 7,386 after matching)	**Odds Ratio (95% CI)**	[a]***P* value**
**0-1 Year**	Osteopenia/Osteoporosis	1,859 (12.4%)	886 (5.9%)	2.3 (2.1-2.5)	< 0.001	826 (11.2%)	388 (5.3%)	2.3 (2.0-2.6)	< 0.001
	Fractures	479 (3.2%)	360 (2.4%)	1.3 (1.2-1.5)	< 0.001	197 (2.7%)	180 (2.4%)	1.1 (0.9-1.3)	0.38
	Urolithiasis	532 (3.6%)	187 (1.3%)	2.9 (2.5-3.4)	< 0.001	234 (3.2%)	99 (1.3%)	2.4 (1.9-3.1)	< 0.001
	MDD	1,395 (9.3%)	1,047 (7.0%)	1.4 (1.3-1.5)	< 0.001	583 (7.9%)	467 (6.3%)	1.3 (1.1-1.4)	< 0.001
	Anxiety Disorders	1,330 (8.9%)	1,026 (6.9%)	1.3 (1.2-1.4)	< 0.001	560 (7.6%)	451 (6.1%)	1.3 (1.1-1.4)	< 0.001
	HTN	6,433 (43.0%)	4,232 (28.3%)	1.9 (1.8-2.0)	< 0.001	2,857 (38.7%)	2,137 (28.9%)	1.5 (1.4-1.7)	< 0.001
	GERD	2,047 (13.7%)	1,476 (9.9%)	1.4 (1.3-1.6)	< 0.001	880 (11.9%)	707 (9.6%)	1.3 (1.2-1.4)	< 0.001
	Malaise/Fatigue	1,412 (9.4%)	755 (5.0%)	2.0 (1.8-2.1)	< 0.001	621 (8.4%)	460 (6.2%)	1.4 (1.2-1.6)	< 0.001
	Joint Pain/Myalgias	2,358 (15.8%)	1,846 (12.3%)	1.3 (1.2-1.4)	< 0.001	1,033 (14.0%)	807 (10.9%)	1.3 (1.2-1.5)	< 0.001
	Constipation	851 (5.7%)	453 (3.0%)	1.9 (1.7-2.2)	< 0.001	347 (4.7%)	243 (3.3%)	1.5 (1.2-1.7)	< 0.001
	Insomnia	584 (3.9%)	351 (2.3%)	1.7 (1.5-1.9)	< 0.001	250 (3.4%)	194 (2.6%)	1.3 (1.1-1.6)	0.007
	Polyuria	599 (4.0%)	377 (2.5%)	1.6 (1.4-1.8)	< 0.001	246 (3.3%)	191 (2.6%)	1.3 (1.1-1.6)	0.008
	Weakness	936 (6.3%)	497 (3.3%)	1.9 (1.7-2.2)	< 0.001	402 (5.4%)	299 (4.0%)	1.4 (1.2-1.6)	< 0.001
	Abdominal Pain	1,439 (9.6%)	918 (6.1%)	1.6 (1.5-1.8)	< 0.001	596 (8.1%)	534 (7.2%)	1.1 (1.0-1.3)	0.06
	Headache	765 (5.1%)	496 (3.3%)	1.6 (1.4-1.8)	< 0.001	309 (4.2%)	268 (3.6%)	1.2 (1.0-1.4)	0.08
	Nausea	538 (3.6%)	275 (1.8%)	2.0 (1.7-2.3)	< 0.001	226 (3.1%)	166 (2.2%)	1.4 (1.1-1.7)	0.002
	Amnesia	523 (3.5%)	355 (2.4%)	1.5 (1.3-1.7)	< 0.001	213 (2.9%)	164 (2.2%)	1.3 (1.1-1.6)	0.01
	Gallstones	266 (1.8%)	149 (1.0%)	1.8 (1.5-2.2)	< 0.001	124 (1.7%)	88 (1.2%)	1.4 (1.1-1.9)	0.01
**1-2 Years**	Osteopenia/Osteoporosis	1,914 (12.8%)	693 (4.6%)	3.0 (2.8-3.3)	< 0.001	838 (11.3%)	379 (5.1%)	2.4 (2.1-2.7)	< 0.001
	Fractures	418 (2.8%)	241 (1.6%)	1.8 (1.5-2.1)	< 0.001	173 (2.3%)	137 (1.9%)	1.3 (1.0-1.6)	0.04
	Urolithiasis	458 (3.1%)	135 (0.9%)	3.5 (2.9-4.2)	< 0.001	212 (2.9%)	60 (0.8%)	3.6 (2.7-4.8)	< 0.001
	MDD	1,346 (9.0%)	734 (4.9%)	1.9 (1.7-2.1)	< 0.001	570 (7.7%)	405 (5.5%)	1.4 (1.3-1.6)	< 0.001
	Anxiety Disorders	1,205 (8.1%)	721 (4.9%)	1.8 (1.6-1.9)	< 0.001	512 (6.9%)	365 (4.9%)	1.4 (1.2-1.6)	< 0.001
	HTN	6,297 (42.1%)	2,991 (20.0%)	2.9 (2.8-3.1)	< 0.001	2,786 (37.7%)	1,763 (23.9%)	1.9 (1.8-2.1)	< 0.001
	GERD	1,877 (12.5%)	1,051 (7.0%)	1.9 (1.8-2.1)	< 0.001	774 (10.5%)	542 (7.3%)	1.5 (1.3-1.7)	< 0.001
	Malaise/Fatigue	1,058 (7.1%)	489 (3.3%)	2.3 (2.0-2.5)	< 0.001	441 (6.0%)	327 (4.4%)	1.4 (1.2-1.6)	< 0.001
	Joint Pain/Myalgias	2,298 (15.4%)	1,264 (8.5%)	2.0 (1.8-2.1)	< 0.001	1,007 (13.6%)	712 (9.6%)	1.5 (1.4-1.6)	< 0.001
	Constipation	706 (4.7%)	298 (2.0%)	2.4 (2.1-2.8)	< 0.001	295 (4.0%)	156 (2.1%)	1.9 (1.6-2.3)	< 0.001
	Insomnia	488 (3.3%)	275 (1.8%)	1.8 (1.6-2.1)	< 0.001	209 (2.8%)	158 (2.1%)	1.3 (1.1-1.6)	0.007
	Polyuria	543 (3.6%)	266 (1.8%)	2.1 (1.8-2.4)	< 0.001	242 (3.3%)	159 (2.2%)	1.5 (1.3-1.9)	< 0.001
	Weakness	636 (4.3%)	297 (2.0%)	2.2 (1.9-2.5)	< 0.001	279 (3.8%)	200 (2.7%)	1.4 (1.2-1.7)	< 0.001
	Abdominal Pain	1,179 (7.9%)	590 (3.9%)	2.1 (1.9-2.3)	< 0.001	489 (6.6%)	349 (4.7%)	1.3 (1.2-1.6)	< 0.001
	Headache	644 (4.3%)	282 (1.9%)	2.3 (2.0-2.7)	< 0.001	275 (3.7%)	181 (2.5%)	1.5 (1.3-1.9)	< 0.001
	Nausea	400 (2.7%)	150 (1.0%)	2.7 (2.2-3.3)	< 0.001	176 (2.4%)	113 (1.5%)	1.6 (1.2-2.0)	< 0.001
	Amnesia	459 (3.1%)	203 (1.4%)	2.3 (1.9-2.7)	< 0.001	194 (2.6%)	125 (1.7%)	1.6 (1.2-2.0)	< 0.001
	Gallstones	189 (1.3%)	74 (0.5%)	2.6 (2.0-3.4)	< 0.001	92 (1.2%)	36 (0.5%)	2.6 (1.7-3.8)	< 0.001
**2-3 Years**	Osteopenia/Osteoporosis	2,059 (13.8%)	575 (3.8%)	4.0 (3.6-4.4)	< 0.001	887 (12.0%)	421 (5.7%)	2.3 (2.0-2.5)	< 0.001
	Fractures	474 (3.2%)	163 (1.1%)	3.0 (2.5-3.6)	< 0.001	186 (2.5%)	127 (1.7%)	1.5 (1.2-1.9)	< 0.001
	Urolithiasis	438 (2.9%)	94 (0.6%)	4.8 (3.8-6.0)	< 0.001	201 (2.7%)	61 (0.8%)	3.4 (2.5-4.5)	< 0.001
	MDD	1,429 (9.6%)	533 (3.6%)	2.9 (2.6-3.2)	< 0.001	591 (8.0%)	409 (5.5%)	1.5 (1.3-1.7)	< 0.001
	Anxiety Disorders	1,272 (8.5%)	531 (3.6%)	2.5 (2.3-2.8)	< 0.001	547 (7.4%)	502 (5.4%)	1.4 (1.2-1.6)	< 0.001
	HTN	6,469 (43.2%)	2,295 (15.3%)	4.2 (4.0-4.4)	< 0.001	2,873 (38.9%)	1,858 (25.2%)	1.9 (1.8-2.0)	< 0.001
	GERD	1,911 (12.8%)	830 (5.5%)	2.5 (2.3-2.8)	< 0.001	791 (10.7%)	609 (8.2%)	1.3 (1.2-1.5)	< 0.001
	Malaise/Fatigue	1,101 (7.4%)	386 (2.6%)	3.0 (2.7-3.4)	< 0.001	494 (6.7%)	304 (4.1%)	1.7 (1.4-1.9)	< 0.001
	Joint Pain/Myalgias	2,313 (15.5%)	952 (6.4%)	2.7 (2.5-2.9)	< 0.001	972 (13.2%)	733 (9.9%)	1.4 (1.2-1.5)	< 0.001
	Constipation	688 (4.6%)	246 (1.6%)	2.9 (2.5-3.3)	< 0.001	280 (3.8%)	183 (2.5%)	1.6 (1.3-1.9)	< 0.001

© 2022 Lorenz FJ et al. *JAMA Network Open.*

	Insomnia	516 (3.5%)	205 (1.4%)	2.6 (2.2-3.0)	< 0.001	220 (3.0%)	165 (2.2%)	1.3 (1.1-1.6)	0.005
	Polyuria	584 (3.9%)	198 (1.3%)	3.0 (2.6-3.6)	< 0.001	235 (3.2%)	160 (2.2%)	1.5 (1.2-1.9)	< 0.001
	Weakness	699 (4.7%)	201 (1.3%)	3.6 (3.1-4.2)	< 0.001	303 (4.1%)	185 (2.5%)	1.7 (1.4-2.0)	< 0.001
	Abdominal Pain	1,193 (8.0%)	474 (3.2%)	2.6 (2.4-3.0)	< 0.001	497 (6.7%)	366 (5.0%)	1.4 (1.2-1.6)	< 0.001
	Headache	603 (4.0%)	233 (1.6%)	2.7 (2.3-3.1)	< 0.001	256 (3.5%)	161 (2.2%)	1.6 (1.3-2.0)	< 0.001
	Nausea	396 (2.6%)	160 (1.1%)	2.5 (2.1-3.0)	< 0.001	157 (2.1%)	89 (1.2%)	1.8 (1.4-2.3)	< 0.001
	Amnesia	471 (3.1%)	174 (1.2%)	2.8 (2.3-3.3)	< 0.001	175 (2.4%)	143 (1.9%)	1.2 (1.0-1.5)	0.07
	Gallstones	194 (1.3%)	70 (0.5%)	2.8 (2.1-3.7)	< 0.001	81 (1.1%)	52 (0.7%)	1.6 (1.1-2.2)	0.01
**Entire Study Period (0-3 Years)**	Osteopenia/Osteoporosis	3,152 (21.1%)	1,426 (9.5%)	2.5 (2.4-2.7)	< 0.001	1,422 (19.3%)	726 (9.8%)	2.2 (2.0-2.4)	< 0.001
	Fractures	992 (6.6%)	632 (4.2%)	1.6 (1.5-1.8)	< 0.001	409 (5.5%)	352 (4.8%)	1.2 (1.0-1.4)	0.03
	Urolithiasis	887 (5.9%)	317 (2.1%)	2.9 (2.6-3.3)	< 0.001	414 (5.6%)	135 (1.8%)	3.2 (2.6-3.9)	< 0.001
	MDD	2,252 (15.1%)	1,468 (9.8%)	1.6 (1.5-1.7)	< 0.001	923 (12.5%)	690 (9.3%)	1.4 (1.2-1.5)	< 0.001
	Anxiety Disorders	2,168 (4.5%)	1,475 (9.9%)	1.5 (1.4-1.7)	< 0.001	942 (12.8%)	709 (9.6%)	1.4 (1.2-1.5)	< 0.001
	HTN	7,944 (53.1%)	5,039 (33.7%)	2.2 (2.1-2.3)	< 0.001	3,603 (48.8%)	2,708 (36.7%)	1.6 (1.5-1.8)	< 0.001
	GERD	3,220 (21.5%)	2,131 (14.6%)	1.7 (1.6-1.8)	< 0.001	1,400 (19.0%)	1,136 (15.4%)	1.3 (1.2-1.4)	< 0.001
	Malaise/Fatigue	2,554 (17.1%)	1,308 (8.7%)	2.1 (2.0-2.3)	< 0.001	1,115 (15.1%)	802 (10.9%)	1.5 (1.3-1.6)	< 0.001
	Joint Pain/Myalgias	4,341 (29.0%)	2,911 (19.5%)	1.7 (1.6-1.8)	< 0.001	1,925 (26.1%)	1,556 (21.1%)	1.3 (1.2-1.4)	< 0.001
	Constipation	1,613 (10.8%)	800 (5.3%)	2.1 (2.0-2.3)	< 0.001	676 (9.2%)	443 (6.0%)	1.6 (1.4-1.8)	< 0.001
	Insomnia	1,075 (7.2%)	612 (4.1%)	1.8 (1.6-2.0)	< 0.001	457 (6.2%)	308 (4.2%)	1.5 (1.3-1.8)	< 0.001
	Polyuria	1,317 (8.8%)	694 (4.6%)	2.0 (1.8-2.2)	< 0.001	551 (7.5%)	392 (5.3%)	1.4 (1.3-1.6)	< 0.001
	Weakness	1,723 (11.5%)	837 (5.6%)	2.2 (2.0-2.4)	< 0.001	757 (10.2%)	552 (7.5%)	1.4 (1.3-1.6)	< 0.001
	Abdominal Pain	2,760 (18.4%)	1,595 (10.7%)	1.9 (1.8-2.2)	< 0.001	1,167 (15.8%)	935 (12.7%)	1.3 (1.2-1.4)	< 0.001
	Headache	1,523 (10.2%)	855 (5.7%)	1.9 (1.7-2.0)	< 0.001	635 (8.6%)	471 (6.4%)	1.4 (1.2-1.6)	< 0.001
	Nausea	1,027 (6.9%)	502 (3.4%)	2.1 (1.9-2.4)	< 0.001	443 (6.0%)	331 (4.5%)	1.4 (1.2-1.6)	< 0.001
	Amnesia	1,040 (7.0%)	592 (4.0%)	1.8 (1.6-2.0)	< 0.001	428 (5.8%)	324 (4.4%)	1.3 (1.2-1.6)	< 0.001
	Gallstones	510 (3.4%)	253 (1.7%_	2.1 (1.8-2.4)	< 0.001	234 (3.2%)	157 (2.1%)	1.5 (1.2-1.8)	< 0.001

Abbreviations: PHP, primary hyperparathyroidism; PTH, parathyroid hormone; MDD, major depressive disorder; HTN, hypertension; GERD, gastroesophageal reflux disease; CI, confidence interval

[a]Statistical significance defined as *P* <0.003

© 2022 Lorenz FJ et al. *JAMA Network Open.*

eTable 7: Comparing Diagnoses and Symptoms in Patients at High Risk of PHP With Hypercalcemia and PTH ≥40 (n = 23,969) or 50 pg/mL (n = 20,176) Without a Documented Diagnosis With Those Diagnosed (n = 13,136)

	**Diagnosis**	**High-Risk PHP PTH ≥40** (n = 13,119 after matching)	**Diagnosed** (n = 13,119 after matching)	**Odds Ratio (95% CI)**	[a]***P* value**	**High-Risk PHP PTH ≥50** (n = 12,842 after matching)	**Diagnosed** (n = 12,842 after matching)	**Odds Ratio (95% CI)**	[a]***P* value**
**0-1 Year**	Osteopenia/Osteoporosis	2,011 (15.3%)	2,837 (21.6%)	0.7 (0.6-0.7)	<0.001	1,970 (15.3%)	2,797 (21.8%)	0.7 (0.6-0.7)	<0.001
	Fractures	556 (4.2%)	544 (4.1%)	1.0 (0.9-1.2)	0.71	551 (4.3%)	530 (4.1%)	1.0 (0.9-1.2)	0.51
	Urolithiasis	665 (5.1%)	1,166 (8.9%)	0.5 (0.5-0.6)	<0.001	620 (4.8%)	1,132 (8.8%)	0.5 (0.5-0.6)	<0.001
	MDD	1,684 (12.8%)	1,657 (12.6%)	1.0 (0.9-1.1)	0.62	1,585 (12.3%)	1,606 (12.5%)	1.0 (0.9-1.1)	0.69
	Anxiety Disorders	1,597 (12.2%)	1,407 (10.7%)	1.2 (1.1-1.2)	<0.001	1,549 (12.1%)	1,359 (10.6%)	1.2 (1.1-1.3)	<0.001
	HTN	7,081 (54.0%)	6,863 (52.3%)	1.1 (1.0-1.1)	0.007	6,804 (53.0%)	6,761 (52.6%)	1.0 (1.0-1.1)	0.59
	GERD	2,312 (17.6%)	2,411 (18.4%)	1.0 (0.9-1.0)	0.11	2,252 (17.5%)	2,364 (18.4%)	0.9 (0.9-1.0)	0.07
	Malaise/Fatigue	1,686 (12.9%)	1,775 (13.5%)	0.9 (0.9-1.0)	0.10	1,593 (12.4%)	1,735 (13.5%)	0.9 (0.8-1.0)	0.008
	Joint Pain/Myalgias	2,793 (21.3%)	2,785 (21.2%)	1.0 (0.9-1.1)	0.90	2,631 (20.5%)	2,725 (21.2%)	1.0 (0.9-1.0)	0.15
	Constipation	974 (7.4%)	1,024 (7.8%)	0.9 (0.9-1.0)	0.24	950 (7.4%)	1,005 (7.8%)	0.9 (0.9-1.0)	0.20
	Insomnia	742 (5.7%)	602 (4.6%)	1.2 (1.1-1.4)	<0.001	699 (5.4%)	583 (4.5%)	1.2 (1.1-1.4)	<0.001
	Polyuria	702 (5.4%)	790 (6.0%)	0.9 (0.8-1.0)	0.02	639 (5.0%)	773 (6.0%)	0.8 (0.7-0.9)	<0.001
	Weakness	1,150 (8.8%)	1,226 (9.3%)	0.9 (0.9-1.0)	0.10	1,068 (8.3%)	1,195 (9.3%)	0.9 (0.8-1.0)	0.005
	Abdominal Pain	1,780 (13.6%)	1,709 (13.0%)	1.0 (1.0-1.1)	0.20	1,683 (13.1%)	1,661 (12.9%)	1.0 (0.9-1.1)	0.68
	Headache	929 (7.1%)	926 (7.1%)	1.0 (0.9-1.1)	0.94	856 (6.7%)	897 (7.0%)	1.0 (0.9-1.0)	0.31
	Nausea	741 (5.6%)	684 (5.2%)	1.1 (1.0-1.2)	0.12	672 (5.2%)	660 (5.1%)	1.0 (0.9-1.1)	0.74
	Amnesia	586 (4.5%)	614 (4.7%)	1.0 (0.8-1.1)	0.41	557 (4.3%)	609 (4.7%)	0.9 (0.8-1.0)	0.12
	Gallstones	294 (2.2%)	307 (2.3%)	1.0 (0.8-1.1)	0.59	293 (2.3%)	301 (2.3%)	1.0 (0.8-1.1)	0.74
**1-2 Years**	Osteopenia/Osteoporosis	2,082 (15.9%)	2,836 (21.6%)	0.7 (0.6-0.7)	<0.001	2,059 (16.0%)	2,795 (21.8%)	0.7 (0.6-0.7)	<0.001
	Fractures	507 (3.9%)	463 (3.5%)	1.1 (1.0-1.2)	0.15	479 (3.7%)	453 (3.5%)	1.1 (0.9-1.2)	0.39
	Urolithiasis	571 (4.4%)	928 (7.1%)	0.6 (0.5-0.7)	<0.001	518 (4.0%)	903 (7.0%)	0.6 (0.5-0.6)	<0.001
	MDD	1,566 (11.9%)	1,520 (11.6%)	1.0 (1.0-1.1)	0.38	1,511 (11.8%)	1,476 (11.5%)	1.0 (1.0-1.1)	0.50
	Anxiety Disorders	1,460 (11.1%)	1,275 (9.7%)	1.2 (1.1-1.3)	<0.001	1,390 (10.8%)	1,236 (9.6%)	1.1 (1.1-1.2)	0.002
	HTN	6,946 (52.9%)	6,532 (49.8%)	1.1 (1.1-1.2)	<0.001	6,679 (52.0%)	6,446 (50.2%)	1.1 (1.0-1.1)	0.004
	GERD	2,102 (16.0%)	2,254 (17.2%)	0.9 (0.9-1.0)	0.01	2,058 (16.0%)	2,211 (17.2%)	0.9 (0.9-1.0)	0.01
	Malaise/Fatigue	1,258 (9.6%)	1,339 (10.2%)	0.9 (0.9-1.0)	0.09	1,206 (9.4%)	1,306 (10.2%)	0.9 (0.8-1.0)	0.04
	Joint Pain/Myalgias	2,640 (20.1%)	2,569 (19.6%)	1.0 (1.0-1.1)	0.27	2,548 (19.8%)	2,506 (19.5%)	1.0 (1.0-1.1)	0.51
	Constipation	765 (5.8%)	815 (6.2%)	0.9 (0.8-1.0)	0.19	747 (5.8%)	799 (6.2%)	0.9 (0.8-1.0)	0.17
	Insomnia	620 (4.7%)	541 (4.1%)	1.2 (1.0-1.3)	0.02	558 (4.3%)	527 (4.1%)	1.1 (0.9-1.2)	0.34
	Polyuria	603 (4.6%)	659 (5.0%)	0.9 (0.8-1.0)	0.11	593 (4.6%)	643 (5.0%)	0.9 (0.8-1.0)	0.14
	Weakness	792 (6.0%)	901 (6.9%)	0.9 (0.8-1.0)	0.006	732 (5.7%)	880 (6.9%)	0.8 (0.7-0.9)	<0.001
	Abdominal Pain	1,385 (10.6%)	1,435 (10.9%)	1.0 (0.9-1.0)	0.32	1,290 (10.0%)	1,394 (10.9%)	0.9 (0.8-1.0)	0.03
	Headache	789 (6.0%)	703 (5.4%)	1.1 (1.0-1.3)	0.02	718 (5.6%)	676 (5.3%)	1.1 (1.0-1.2)	0.25
	Nausea	522 (4.0%)	472 (3.6%)	1.1 (1.0-1.3)	0.11	467 (3.6%)	458 (3.6%)	1.0 (0.9-1.2)	0.76
	Amnesia	487 (3.7%)	485 (3.7%)	1.0 (0.9-1.1)	0.95	458 (3.6%)	476 (3.7%)	1.0 (0.8-1.1)	0.55
	Gallstones	210 (1.6%)	234 (1.8%)	0.9 (0.7-1.1)	0.25	201 (1.6%)	229 (1.8%)	0.9 (0.7-1.1)	0.17
**2-3 Years**	Osteopenia/Osteoporosis	2,237 (17.1%)	3,174 (24.2%)	0.6 (0.6-0.7)	<0.001	2,198 (17.1%)	3,132 (24.4%)	0.6 (0.6-0.7)	<0.001
	Fractures	546 (4.2%)	496 (3.8%)	1.1 (1.0-1.3)	0.11	506 (3.9%)	485 (3.8%)	1.0 (0.9-1.2)	0.50
	Urolithiasis	573 (4.4%)	952 (7.3%)	0.6 (0.5-0.6)	<0.001	525 (4.1%)	923 (7.2%)	0.6 (0.5-0.6)	<0.001
	MDD	1,658 (12.6%)	1,558 (11.9%)	1.1 (1.0-1.2)	0.06	1,583 (12.3%)	1,509 (11.8%)	1.1 (1.0-1.1)	0.16
	Anxiety Disorders	1,518 (11.6%)	1,361 (10.4%)	1.1 (1.0-1.2)	0.002	1,462 (11.4%)	1,314 (10.2%)	1.1 (1.0-1.2)	0.003
	HTN	7,063 (53.8%)	6,625 (50.5%)	1.1 (1.1-1.2)	<0.001	6,796 (52.9%)	6,538 (50.9%)	1.1 (1.0-1.1)	0.001
	GERD	2,174 (16.6%)	2,308 (17.6%)	0.9 (0.9-1.0)	0.03	2,135 (16.6%)	2,250 (17.5%)	0.9 (0.9-1.0)	0.06
	Malaise/Fatigue	1,271 (9.7%)	1,383 (10.5%)	0.9 (0.8-1.0)	0.02	1,234 (9.6%)	1,356 (10.6%)	0.9 (0.8-1.0)	0.01
	Joint Pain/Myalgias	2,637 (20.1%)	2,615 (19.9%)	1.0 (1.0-1.1)	0.73	2,508 (19.5%)	2,560 (19.9%)	1.0 (0.9-1.0)	0.41
	Constipation	785 (6.0%)	775 (5.9%)	1.0 (0.9-1.1)	0.79	775 (6.0%)	762 (5.9%)	1.0 (0.9-1.1)	0.73
	Insomnia	633 (4.8%)	577 (4.4%)	1.1 (1.0-1.2)	0.10	598 (4.7%)	558 (4.3%)	1.1 (1.0-1.2)	0.23

© 2022 Lorenz FJ et al. *JAMA Network Open.*

	Polyuria	657 (5.0%)	665 (5.1%)	1.0 (0.9-1.1)	0.82	628 (4.9%)	656 (5.1%)	1.0 (0.9-1.1)	0.42
	Weakness	841 (6.4%)	845 (6.4%)	1.0 (0.9-1.1)	0.92	802 (6.2%)	828 (6.4%)	1.0 (0.9-1.1)	0.51
	Abdominal Pain	1,410 (10.7%)	1,369 (10.4%)	1.0 (1.0-1.1)	0.41	1,333 (10.4%)	1,334 (10.4%)	1.0 (0.9-1.1)	0.98
	Headache	753 (5.7%)	721 (5.5%)	1.0 (0.9-1.2)	0.39	666 (5.2%)	698 (5.4%)	1.0 (0.9-1.1)	0.37
	Nausea	509 (3.9%)	475 (3.6%)	1.1 (0.9-1.2)	0.27	467 (3.6%)	462 (3.6%)	1.0 (0.9-1.2)	0.87
	Amnesia	511 (3.9%)	506 (3.9%)	1.0 (0.9-1.1)	0.87	488 (3.8%)	498 (3.9%)	1.0 (0.9-1.1)	0.75
	Gallstones	227 (1.7%)	226 (1.7%)	1.0 (0.8-1.2)	0.96	211 (1.6%)	224 (1.7%)	0.9 (0.8-1.1)	0.53
**Entire Study Period (0-3 Years)**	Osteopenia/Osteoporosis	3,355 (25.6%)	4,856 (37.0%)	0.6 (0.6-0.6)	<0.001	3,326 (25.9%)	4,787 (37.3%)	0.6 (0.6-0.6)	<0.001
	Fractures	1,141 (8.7%)	1,112 (8.5%)	1.0 (0.9-1.1)	0.52	1,091 (8.5%)	1,089 (8.5%)	1.0 (0.9-1.1)	0.96
	Urolithiasis	1,101 (8.4%)	1,905 (14.5%)	0.5 (0.5-0.6)	<0.001	1,010 (7.9%)	1,847 (14.4%)	0.5 (0.5-0.6)	<0.001
	MDD	2,646 (20.2%)	2,675 (20.4%)	1.0 (0.9-1.0)	0.66	2,528 (19.7%)	2,599 (20.2%)	1.0 (0.9-1.0)	0.27
	Anxiety Disorders	2,589 (19.7%)	2,395 (18.3%)	1.1 (1.0-1.2)	0.002	2,485 (19.4%)	2,320 (18.1%)	1.1 (1.0-1.2)	0.008
	HTN	8,623 (65.7%)	8,568 (65.3%)	1.0 (1.0-1.1)	0.47	8,371 (65.2%)	8,425 (65.6%)	1.0 (0.9-1.0)	0.48
	GERD	3,573 (27.2%)	3,906 (29.8%)	0.9 (0.8-0.9)	<0.001	3,515 (27.4%)	3,823 (29.8%)	0.9 (0.8-0.9)	<0.001
	Malaise/Fatigue	2,986 (22.8%)	3,286 (25.0%)	0.9 (0.8-0.9)	<0.001	2,859 (22.3%)	3,208 (25.0%)	0.9 (0.8-0.9)	<0.001
	Joint Pain/Myalgias	4,968 (37.9%)	5,076 (38.7%)	1.0 (0.9-1.0)	0.17	4,771 (37.2%)	4,966 (38.7%)	0.9 (0.9-1.0)	0.01
	Constipation	1,801 (13.7%)	1,922 (14.7%)	0.9 (0.9-1.0)	0.03	1,779 (13.9%)	1,887 (14.7%)	0.9 (0.9-1.0)	0.05
	Insomnia	1,320 (10.1%)	1,150 (8.8%)	1.2 (1.1-1.3)	<0.001	1,236 (9.6%)	1,116 (8.7%)	1.1 (1.0-1.2)	0.009
	Polyuria	1,514 (11.5%)	1,666 (12.7%)	0.9 (0.8-1.0)	0.004	1,432 (11.2%)	1,634 (12.7%)	0.9 (0.8-0.9)	<0.001
	Weakness	2,065 (15.7%)	2,266 (17.3%)	0.9 (0.8-1.0)	<0.001	1,954 (15.2%)	2,212 (17.2%)	0.9 (0.8-0.9)	<0.001
	Abdominal Pain	3,221 (24.6%)	3,292 (25.1%)	1.0 (0.9-1.0)	0.31	3,075 (23.9%)	3,203 (24.9%)	0.9 (0.9-1.0)	0.07
	Headache	1,822 (13.9%)	1,789 (13.6%)	1.0 (1.0-1.1)	0.55	1,685 (13.1%)	1,737 (13.5%)	1.0 (0.9-1.0)	0.34
	Nausea	1,324 (10.1%)	1,301 (9.9%)	1.0 (0.9-1.1)	0.64	1,214 (9.5%)	1,262 (9.8%)	1.0 (0.9-1.0)	0.31
	Amnesia	1,170 (8.9%)	1,157 (8.8%)	1.0 (0.9-1.1)	0.78	1,104 (8.6%)	1,140 (8.9%)	1.0 (0.9-1.1)	0.43
	Gallstones	567 (4.3%)	579 (4.4%)	1.0 (0.9-1.1)	0.72	550 (4.3%)	571 (4.4%)	1.0 (0.9-1.1)	0.52

Abbreviations: PHP, primary hyperparathyroidism; PTH, parathyroid hormone; MDD, major depressive disorder; HTN, hypertension; GERD, gastroesophageal reflux disease; CI, confidence interval

[a]Statistical significance defined as P <0.003

© 2022 Lorenz FJ et al. *JAMA Network Open.*

eTable 8: Comparing Diagnoses and Symptoms in Patients at High Risk of PHP With Hypercalcemia and PTH ≥65 (n = 14,959) or 100 pg/mL (n = 7,387) Without a Documented Diagnosis With Those Diagnosed (n = 13,136)

	**Diagnosis**	**High-Risk PHP PTH ≥65** (n = 11,076 after matching)	**Diagnosed** (n = 11,076 after matching)	**Odds Ratio (95% CI)**	[a]***P* value**	**High-Risk PHP PTH ≥100** (n = 5,686 after matching)	**Diagnosed** (n = 5,686 after matching)	**Odds Ratio (95% CI)**	[a]***P* value**
**0-1 Year**	Osteopenia/Osteoporosis	1,698 (15.3%)	2,464 (22.2%)	0.6 (0.6-0.7)	<0.001	809 (14.2%)	1,250 (22.0%)	0.6 (0.5-0.6)	<0.001
	Fractures	434 (3.9%)	462 (4.2%)	0.9 (0.8-1.1)	0.34	191 (3.4%)	242 (4.3%)	0.8 (0.6-0.9)	0.01
	Urolithiasis	496 (4.5%)	944 (8.5%)	0.5 (0.4-0.6)	<0.001	227 (4.0%)	445 (7.8%)	0.5 (0.4-0.6)	<0.001
	MDD	1,275 (11.5%)	1,353 (12.2%)	0.9 (0.9-1.0)	0.11	569 (10.0%)	665 (11.7%)	0.8 (0.7-0.9)	0.004
	Anxiety Disorders	1,229 (11.1%)	1,159 (10.5%)	1.1 (1.0-1.2)	0.13	547 (9.6%)	599 (10.5%)	0.9 (0.8-1.0)	0.11
	HTN	5,854 (52.9%)	5,945 (53.7%)	1.0 (0.9-1.0)	0.22	2,803 (49.3%)	3,100 (54.5%)	0.8 (0.8-0.9)	<0.001
	GERD	1,875 (16.9%)	2,049 (18.5%)	0.9 (0.8-1.0)	0.002	860 (15.1%)	1,047 (18.4%)	0.8 (0.7-0.9)	<0.001
	Malaise/Fatigue	1,302 (11.8%)	1,505 (13.6%)	0.8 (0.8-0.9)	<0.001	604 (10.6%)	774 (13.6%)	0.8 (0.7-0.8)	<0.001
	Joint Pain/Myalgias	2,122 (19.2%)	2,352 (21.2%)	0.9 (0.8-0.9)	<0.001	1,009 (17.7%)	1,159 (20.4%)	0.8 (0.8-0.9)	<0.001
	Constipation	776 (7.0%)	883 (8.0%)	0.9 (0.8-1.0)	0.006	341 (6.0%)	468 (8.2%)	0.7 (0.6-0.8)	<0.001
	Insomnia	531 (4.8%)	504 (4.6%)	1.1 (0.9-1.2)	0.39	242 (4.3%)	267 (4.7%)	0.9 (0.8-1.1)	0.26
	Polyuria	542 (4.9%)	681 (6.1%)	0.8 (0.7-0.9)	<0.001	243 (4.3%)	347 (6.1%)	0.7 (0.6-0.8)	<0.001
	Weakness	860 (7.8%)	1,040 (9.4%)	0.8 (0.7-0.9)	<0.001	394 (6.9%)	538 (9.5%)	0.7 (0.6-0.8)	<0.001
	Abdominal Pain	1,302 (11.8%)	1,423 (12.8%)	0.9 (0.8-1.0)	0.01	575 (10.1%)	702 (12.3%)	0.8 (0.7-0.9)	<0.001
	Headache	695 (6.3%)	769 (6.9%)	0.9 (0.8-1.0)	0.05	299 (5.3%)	388 (6.8%)	0.8 (0.6-0.9)	<0.001
	Nausea	512 (4.6%)	560 (5.1%)	0.9 (0.8-1.0)	0.13	221 (3.9%)	282 (5.0%)	0.8 (0.6-0.9)	0.005
	Amnesia	480 (4.3%)	536 (4.8%)	0.9 (0.8-1.0)	0.07	209 (3.7%)	286 (5.0%)	0.7 (0.6-0.9)	<0.001
	Gallstones	239 (2.2%)	253 (2.3%)	0.9 (0.8-1.1)	0.52	118 (2.1%)	130 (2.3%)	0.9 (0.7-1.2)	0.44
**1-2 Years**	Osteopenia/Osteoporosis	1,760 (15.9%)	2,462 (22.2%)	0.7 (0.6-0.7)	<0.001	827 (14.5%)	1,243 (21.9%)	0.6 (0.6-0.7)	<0.001
	Fractures	377 (3.4%)	386 (3.5%)	1.0 (0.8-1.1)	0.74	171 (3.0%)	185 (3.3%)	0.9 (0.7-1.1)	0.45
	Urolithiasis	431 (3.9%)	756 (6.8%)	0.6 (0.5-0.6)	<0.001	207 (3.6%)	366 (6.4%)	0.5 (0.5-0.7)	<0.001
	MDD	1,234 (11.1%)	1,262 (11.4%)	1.0 (0.9-1.1)	0.55	555 (9.8%)	620 (10.9%)	0.9 (0.8-1.0)	0.05
	Anxiety Disorders	1,111 (10.0%)	1,055 (9.5%)	1.1 (1.0-1.2)	0.21	505 (8.9%)	532 (9.4%)	0.9 (0.8-1.1)	0.38
	HTN	5,727 (51.7%)	5,670 (51.2%)	1.0 (1.0-1.1)	0.44	2,733 (48.1%)	2,913 (51.2%)	0.9 (0.8-0.9)	<0.001
	GERD	1,705 (15.4%)	1,897 (17.1%)	0.9 (0.8-0.9)	<0.001	756 (13.3%)	993 (17.5%)	0.7 (0.7-0.8)	<0.001
	Malaise/Fatigue	961 (8.7%)	1,145 (10.3%)	0.8 (0.8-0.9)	<0.001	432 (7.6%)	566 (10.0%)	0.7 (0.7-0.8)	<0.001
	Joint Pain/Myalgias	2,083 (18.8%)	2,166 (19.6%)	1.0 (0.9-1.0)	0.16	985 (17.3%)	1,116 (19.6%)	0.9 (0.8-0.9)	0.002
	Constipation	643 (5.8%)	709 (6.4%)	0.9 (0.8-1.0)	0.06	290 (5.1%)	370 (6.5%)	0.8 (0.7-0.9)	0.001
	Insomnia	447 (4.0%)	457 (4.1%)	1.0 (0.9-1.1)	0.73	205 (3.6%)	243 (4.3%)	0.8 (0.7-1.0)	0.07
	Polyuria	497 (4.5%)	574 (5.2%)	0.9 (0.8-1.0)	0.02	236 (4.2%)	285 (5.0%)	0.8 (0.7-1.0)	0.03
	Weakness	580 (5.2%)	776 (7.0%)	0.7 (0.7-0.8)	<0.001	271 (4.8%)	391 (6.9%)	0.7 (0.6-0.8)	<0.001
	Abdominal Pain	1,062 (9.6%)	1,208 (10.9%)	0.9 (0.8-0.9)	0.001	475 (8.4%)	632 (11.1%)	0.7 (0.6-0.8)	<0.001
	Headache	587 (5.3%)	582 (5.3%)	1.0 (0.9-1.1)	0.88	270 (4.7%)	296 (5.2%)	0.9 (0.8-1.1)	0.26
	Nausea	372 (3.4%)	385 (3.5%)	1.0 (0.8-1.1)	0.63	173 (3.0%)	200 (3.5%)	0.9 (0.7-1.1)	0.16
	Amnesia	417 (3.8%)	429 (3.9%)	1.0 (0.8-1.1)	0.67	190 (3.3%)	242 (4.3%)	0.8 (0.6-0.9)	0.01
	Gallstones	170 (1.5%)	204 (1.8%)	0.8 (0.7-1.0)	0.07	88 (1.5%)	118 (2.1%)	0.7 (0.6-1.0)	0.03
**2-3 Years**	Osteopenia/Osteoporosis	1,888 (17.0%)	2,747 (24.8%)	0.6 (0.6-0.7)	<0.001	874 (15.4%)	1,396 (24.6%)	0.6 (0.5-0.6)	<0.001
	Fractures	429 (3.9%)	427 (3.9%)	1.0 (0.9-1.2)	0.94	182 (3.2%)	216 (3.8%)	0.8 (0.7-1.0)	0.08
	Urolithiasis	411 (3.7%)	767 (6.9%)	0.5 (0.5-0.6)	<0.001	195 (3.4%)	361 (6.3%)	0.5 (0.4-0.6)	<0.001
	MDD	1,307 (11.8%)	1,290 (11.6%)	1.0 (0.9-1.1)	0.72	574 (10.1%)	639 (11.2%)	0.9 (0.8-1.0)	0.05
	Anxiety Disorders	1,182 (10.7%)	1,118 (10.1%)	1.1 (1.0-1.2)	0.16	536 (9.4%)	556 (9.8%)	1.0 (0.8-1.1)	0.52
	HTN	5,858 (52.9%)	5,780 (52.2%)	1.0 (1.0-1.1)	0.29	2,813 (49.5%)	2,991 (52.6%)	0.9 (0.8-0.9)	<0.001
	GERD	1,741 (15.7%)	1,951 (17.6%)	0.9 (0.8-0.9)	<0.001	773 (13.6%)	1,009 (17.7%)	0.7 (0.7-0.8)	<0.001
	Malaise/Fatigue	1,009 (9.1%)	1,174 (10.6%)	0.8 (0.8-0.9)	<0.001	484 (8.5%)	607 (10.7%)	0.8 (0.7-0.9)	<0.001
	Joint Pain/Myalgias	2,099 (19.0%)	2,209 (19.9%)	0.9 (0.9-1.0)	0.06	953 (16.8%)	1,150 (20.2%)	0.8 (0.7-0.9)	<0.001
	Constipation	608 (5.5%)	684 (6.2%)	0.9 (0.8-1.0)	0.03	271 (4.8%)	358 (6.3%)	0.7 (0.6-0.9)	<0.001
	Insomnia	473 (4.3%)	480 (4.3%)	1.0 (0.9-1.1)	0.82	214 (3.8%)	262 (4.6%)	0.8 (0.7-1.0)	0.02

© 2022 Lorenz FJ et al. *JAMA Network Open.*

	Polyuria	532 (4.8%)	584 (5.3%)	0.9 (0.8-1.0)	0.11	230 (4.0%)	308 (5.4%)	0.7 (0.6-0.9)	<0.001
	Weakness	641 (5.8%)	721 (6.5%)	0.9 (0.8-1.0)	0.03	293 (5.2%)	374 (6.6%)	0.8 (0.7-0.9)	0.001
	Abdominal Pain	1,076 (9.7%)	1,147 (10.4%)	0.9 (0.9-1.0)	0.11	482 (8.5%)	573 (10.1%)	0.8 (0.7-0.9)	0.003
	Headache	544 (4.9%)	593 (5.4%)	0.9 (0.8-1.0)	0.14	248 (4.4%)	299 (5.3%)	0.8 (0.7-1.0)	0.03
	Nausea	364 (3.3%)	384 (3.5%)	0.9 (0.8-1.1)	0.46	152 (2.7%)	205 (3.6%)	0.7 (0.6-0.9)	0.004
	Amnesia	419 (3.8%)	440 (4.0%)	1.0 (0.8-1.1)	0.46	170 (3.0%)	242 (4.3%)	0.7 (0.6-0.8)	<0.001
	Gallstones	174 (1.6%)	188 (1.7%)	0.9 (0.8-1.1)	0.46	78 (1.4%)	105 (1.8%)	0.7 (0.6-1.0)	0.04
**Entire Study Period (0-3 Years)**	Osteopenia/Osteoporosis	2,893 (26.1%)	4,201 (37.9%)	0.6 (0.5-0.6)	<0.001	1,399 (24.6%)	2,132 (37.5%)	0.5 (0.5-0.6)	<0.001
	Fractures	903 (8.2%)	938 (8.5%)	1.0 (0.9-1.1)	0.39	400 (7.0%)	471 (8.3%)	0.8 (0.7-1.0)	0.01
	Urolithiasis	827 (7.5%)	1,556 (14.0%)	0.5 (0.5-0.5)	<0.001	401 (7.1%)	756 (13.3%)	0.5 (0.4-0.6)	<0.001
	MDD	2,058 (18.6%)	2,210 (20.0%)	0.9 (0.9-1.0)	0.01	898 (15.8%)	1,097 (19.3%)	0.8 (0.7-0.9)	<0.001
	Anxiety Disorders	2,005 (18.1%)	1,981 (17.9%)	1.0 (0.9-1.1)	0.67	923 (16.2%)	1,012 (17.8%)	0.9 (0.8-1.0)	0.03
	HTN	7,215 (65.1%)	7,378 (66.6%)	0.9 (0.9-1.0)	0.02	3,528 (62.0%)	3,826 (67.3%)	0.8 (0.7-0.9)	<0.001
	GERD	2,933 (26.5%)	3,286 (29.7%)	0.9 (0.8-0.9)	<0.001	1,370 (24.1%)	1,704 (30.0%)	0.7 (0.7-0.8)	<0.001
	Malaise/Fatigue	2,337 (21.1%)	2,778 (25.1%)	0.8 (0.8-0.9)	<0.001	1,088 (19.1%)	1,408 (24.8%)	0.7 (0.7-0.8)	<0.001
	Joint Pain/Myalgias	3,931 (35.5%)	4,274 (38.6%)	0.9 (0.8-0.9)	<0.001	1,882 (33.1%)	2,149 (37.8%)	0.8 (0.8-0.9)	<0.001
	Constipation	1,459 (13.2%)	1,669 (15.1%)	0.9 (0.8-0.9)	<0.001	661 (11.6%)	883 (15.5%)	0.7 (0.6-0.8)	<0.001
	Insomnia	974 (8.8%)	969 (8.7%)	1.0 (0.9-1.1)	0.91	445 (7.8%)	519 (9.1%)	0.8 (0.7-1.0)	0.01
	Polyuria	1,203 (10.9%)	1,448 (13.1%)	0.8 (0.7-0.9)	<0.001	539 (9.5%)	742 (13.0%)	0.7 (0.6-0.8)	<0.001
	Weakness	1,573 (14.2%)	1,926 (17.4%)	0.8 (0.7-0.8)	<0.001	736 (12.9%)	981 (17.3%)	0.7 (0.6-0.8)	<0.001
	Abdominal Pain	2,503 (22.6%)	2,754 (24.9%)	0.9 (0.8-0.9)	<0.001	1,131 (19.9%)	1,398 (24.6%)	0.8 (0.7-0.8)	<0.001
	Headache	1,387 (12.5%)	1,478 (13.3%)	0.9 (0.9-1.0)	0.07	619 (10.9%)	740 (13.0%)	0.8 (0.7-0.9)	<0.001
	Nausea	955 (8.6%)	1,064 (9.6%)	0.9 (0.8-1.0)	0.01	432 (7.6%)	549 (9.7%)	0.8 (0.7-0.9)	<0.001
	Amnesia	946 (8.5%)	1,008 (9.1%)	0.9 (0.8-1.0)	0.14	420 (7.4%)	547 (9.6%)	0.7 (0.7-0.9)	<0.001
	Gallstones	455 (4.1%)	487 (4.4%)	0.9 (0.8-1.1)	0.29	225 (4.0%)	266 (4.7%)	0.8 (0.7-1.0)	0.06

Abbreviations: PHP, primary hyperparathyroidism; PTH, parathyroid hormone; MDD, major depressive disorder; HTN, hypertension; GERD, gastroesophageal reflux disease; CI, confidence interval

[a]Statistical significance defined as *P* <0.003

© 2022 Lorenz FJ et al. *JAMA Network Open.*

eTable 9: Comparing Diagnoses and Symptoms in Patients at High Risk of PHP With Hypercalcemia and PTH ≥40 (n = 23,969) or 50 pg/mL (n = 20,176) Without a Documented Diagnosis With Those Diagnosed With Similar PTH Levels (n = 12,294 for PTH ≥40 and n = 11,968 for ≥50 pg/mL)

	**Diagnosis**	**High-Risk PHP PTH ≥40** (n = 12,277 after matching)	**Diagnosed PTH ≥40** (n = 12,277 after matching)	**Odds Ratio (95% CI)**	[a]***P* value**	**High-Risk PHP PTH ≥50** (n = 11,876 after matching)	**Diagnosed PTH ≥50** (n = 11,876 after matching)	**Odds Ratio (95% CI)**	[a]***P* value**
**0-1 Year**	Osteopenia/Osteoporosis	1,900 (15.5%)	2,677 (21.8%)	0.7 (0.6-0.7)	<0.001	1,826 (15.4%)	2,589 (21.8%)	0.7 (0.6-0.7)	<0.001
	Fractures	515 (4.2%)	509 (4.1%)	1.0 (0.9-1.1)	0.85	511 (4.3%)	492 (4.1%)	1.0 (0.9-1.2)	0.54
	Urolithiasis	621 (5.1%)	1,099 (9.0%)	0.5 (0.5-0.6)	<0.001	576 (4.9%)	1,043 (8.8%)	0.5 (0.5-0.6)	<0.001
	MDD	1,567 (12.8%)	1,560 (12.7%)	1.0 (0.9-1.1)	0.89	1,481 (12.5%)	1,510 (12.7%)	1.0 (0.9-1.1)	0.57
	Anxiety Disorders	1,501 (12.2%)	1,328 (10.8%)	1.1 (1.1-1.2)	<0.001	1,453 (12.2%)	1,273 (10.7%)	1.2 (1.1-1.3)	<0.001
	HTN	6,607 (53.8%)	6,357 (51.8%)	1.1 (1.0-1.1)	0.001	6,272 (52.8%)	6,153 (51.8%)	1.0 (1.0-1.1)	0.12
	GERD	2,167 (17.7%)	2,259 (18.4%)	1.0 (0.9-1.0)	0.13	2,082 (17.5%)	2,185 (18.4%)	0.9 (0.9-1.0)	0.08
	Malaise/Fatigue	1,566 (12.8%)	1,673 (13.6%)	0.9 (0.9-1.0)	0.04	1,484 (12.5%)	1,581 (13.3%)	0.9 (0.9-1.0)	0.06
	Joint Pain/Myalgias	2,617 (21.3%)	2,592 (21.1%)	1.0 (1.0-1.1)	0.70	2,442 (20.6%)	2,509 (21.1%)	1.0 (0.9-1.0)	0.28
	Constipation	915 (7.5%)	958 (7.8%)	1.0 (0.9-1.0)	0.30	879 (7.4%)	919 (7.7%)	1.0 (0.9-1.0)	0.33
	Insomnia	705 (5.7%)	562 (4.6%)	1.3 (1.1-1.4)	<0.001	651 (5.5%)	544 (4.6%)	1.2 (1.1-1.4)	0.001
	Polyuria	659 (5.4%)	740 (6.0%)	0.9 (0.8-1.0)	0.03	581 (4.9%)	714 (6.0%)	0.8 (0.7-0.9)	<0.001
	Weakness	1,072 (8.7%)	1,146 (9.3%)	0.9 (0.9-1.0)	0.10	990 (8.3%)	1,090 (9.2%)	0.9 (0.8-1.0)	0.02
	Abdominal Pain	1,668 (13.6%)	1,601 (13.0%)	1.0 (1.0-1.1)	0.21	1,568 (13.2%)	1,541 (13.0%)	1.0 (0.9-1.1)	0.60
	Headache	866 (7.1%)	859 (7.0%)	1.0 (0.9-1.1)	0.86	798 (6.7%)	823 (6.9%)	1.0 (0.9-1.1)	0.52
	Nausea	690 (5.6%)	640 (5.2%)	1.1 (1.0-1.2)	0.16	628 (5.3%)	604 (5.1%)	1.0 (0.9-1.2)	0.48
	Amnesia	544 (4.4%)	572 (4.7%)	0.9 (0.8-1.1)	0.39	503 (4.2%)	549 (4.6%)	0.9 (0.8-1.0)	0.15
	Gallstones	274 (2.2%)	291 (2.4%)	0.9 (0.8-1.1)	0.47	275 (2.3%)	281 (2.4%)	1.0 (0.8-1.2)	0.80
**1-2 Years**	Osteopenia/Osteoporosis	1,975 (16.1%)	2,665 (21.7%)	0.7 (0.6-0.7)	<0.001	1,903 (16.0%)	2,586 (21.8%)	0.7 (0.6-0.7)	<0.001
	Fractures	482 (3.9%)	431 (3.5%)	1.1 (1.0-1.3)	0.09	446 (3.8%)	412 (3.5%)	1.1 (0.9-1.2)	0.24
	Urolithiasis	529 (4.3%)	875 (7.1%)	0.6 (0.5-0.7)	<0.001	473 (4.0%)	831 (7.0%)	0.6 (0.5-0.6)	<0.001
	MDD	1,462 (11.9%)	1,440 (11.7%)	1.0 (0.9-1.1)	0.66	1,404 (11.8%)	1,391 (11.7%)	1.0 (0.9-1.1)	0.79
	Anxiety Disorders	1,384 (11.3%)	1,208 (9.8%)	1.2 (1.1-1.3)	<0.001	1,296 (10.9%)	1,160 (9.8%)	1.1 (1.0-1.2)	0.004
	HTN	6,483 (52.8%)	6,082 (49.5%)	1.1 (1.1-1.2)	<0.001	6,178 (52.0%)	5,890 (49.6%)	1.1 (1.0-1.2)	<0.001
	GERD	1,987 (16.2%)	2,125 (17.3%)	0.9 (0.9-1.0)	0.02	1,902 (16.0%)	2,056 (17.3%)	0.9 (0.9-1.0)	0.007
	Malaise/Fatigue	1,180 (9.6%)	1,264 (10.3%)	0.9 (0.9-1.0)	0.07	1,124 (9.5%)	1,204 (10.1%)	0.9 (0.9-1.0)	0.08
	Joint Pain/Myalgias	2,471 (20.1%)	2,418 (19.7%)	1.0 (1.0-1.1)	0.40	2,368 (19.9%)	2,326 (19.6%)	1.0 (1.0-1.1)	0.49
	Constipation	710 (5.8%)	771 (6.3%)	0.9 (0.8-1.0)	0.10	684 (5.8%)	744 (6.3%)	0.9 (0.8-1.0)	0.10
	Insomnia	582 (4.7%)	504 (4.1%)	1.2 (1.0-1.3)	0.02	523 (4.4%)	486 (4.1%)	1.1 (1.0-1.2)	0.23
	Polyuria	556 (4.5%)	612 (5.0%)	0.9 (0.8-1.0)	0.09	528 (4.4%)	588 (5.0%)	0.9 (0.8-1.0)	0.07
	Weakness	742 (6.0%)	841 (6.9%)	0.9 (0.8-1.0)	0.01	686 (5.8%)	799 (6.7%)	0.8 (0.8-0.9)	0.002
	Abdominal Pain	1,286 (10.5%)	1,328 (10.8%)	1.0 (0.9-1.0)	0.38	1,204 (10.1%)	1,280 (10.8%)	0.9 (0.9-1.0)	0.11
	Headache	734 (6.0%)	650 (5.3%)	1.1 (1.0-1.3)	0.02	665 (5.6%)	616 (5.2%)	1.1 (1.0-1.2)	0.16
	Nausea	485 (4.0%)	437 (3.6%)	1.1 (1.0-1.3)	0.11	441 (3.7%)	418 (3.5%)	1.1 (0.9-1.2)	0.42
	Amnesia	470 (3.8%)	450 (3.7%)	1.0 (0.9-1.2)	0.50	419 (3.5%)	433 (3.6%)	1.0 (0.8-1.1)	0.63
	Gallstones	197 (1.6%)	225 (1.8%)	0.9 (0.7-1.1)	0.17	182 (1.5%)	219 (1.8%)	0.8 (0.7-1.0)	0.06
**2-3 Years**	Osteopenia/Osteoporosis	2,107 (17.2%)	2,992 (24.4%)	0.6 (0.6-0.7)	<0.001	2,039 (17.2%)	2,898 (24.4%)	0.6 (0.6-0.7)	<0.001
	Fractures	519 (4.2%)	460 (3.7%)	1.1 (1.0-1.3)	0.05	475 (4.0%)	444 (3.7%)	1.1 (0.9-1.2)	0.30
	Urolithiasis	527 (4.3%)	872 (7.1%)	0.6 (0.5-0.7)	<0.001	474 (4.0%)	833 (7.0%)	0.6 (0.5-0.6)	<0.001
	MDD	1,545 (12.6%)	1,477 (12.0%)	1.1 (1.0-1.1)	0.19	1,479 (12.5%)	1,431 (12.0%)	1.0 (1.0-1.1)	0.34
	Anxiety Disorders	1,426 (11.6%)	1,290 (10.5%)	1.1 (1.0-1.2)	0.006	1,368 (11.5%)	1,243 (10.5%)	1.1 (1.0-1.2)	0.01
	HTN	6,581 (53.6%)	6,189 (50.4%)	1.1 (1.1-1.2)	<0.001	6,271 (52.8%)	5,991 (50.4%)	1.1 (1.0-1.2)	<0.001
	GERD	2,026 (16.5%)	2,169 (17.7%)	0.9 (0.9-1.0)	0.02	1,978 (16.7%)	2,090 (17.6%)	0.9 (0.9-1.0)	0.05
	Malaise/Fatigue	1,191 (9.7%)	1,299 (10.6%)	0.9 (0.8-1.0)	0.02	1,151 (9.7%)	1,246 (10.5%)	0.9 (0.8-1.0)	0.04
	Joint Pain/Myalgias	2,467 (20.1%)	2,458 (20.0%)	1.0 (0.9-1.1)	0.89	2,327 (19.6%)	2,373 (20.0%)	1.0 (0.9-1.0)	0.45

© 2022 Lorenz FJ et al. *JAMA Network Open.*

	Constipation	727 (5.9%)	734 (6.0%)	1.0 (0.9-1.1)	0.85	712 (6.0%)	712 (6.0%)	1.0 (0.9-1.1)	1.0
	Insomnia	598 (4.9%)	531 (4.3%)	1.1 (1.0-1.3)	0.04	560 (4.7%)	514 (4.3%)	1.1 (1.0-1.2)	0.15
	Polyuria	607 (4.9%)	623 (5.1%)	1.0 (0.9-1.1)	0.64	576 (4.9%)	600 (5.1%)	1.0 (0.9-1.1)	0.47
	Weakness	792 (6.5%)	796 (6.5%)	1.0 (0.9-1.1)	0.92	750 (6.3%)	756 (6.4%)	1.0 (0.9-1.1)	0.87
	Abdominal Pain	1,313 (10.7%)	1,277 (10.4%)	1.0 (1.0-1.1)	0.45	1,240 (10.4%)	1,229 (10.3%)	1.0 (0.9-1.1)	0.82
	Headache	697 (5.7%)	670 (5.5%)	1.0 (0.9-1.2)	0.45	621 (5.2%)	645 (5.4%)	1.0 (0.9-1.1)	0.49
	Nausea	474 (3.9%)	445 (3.6%)	1.1 (0.9-1.2)	0.33	424 (3.6%)	427 (3.6%)	1.0 (0.9-1.1)	0.92
	Amnesia	480 (3.9%)	471 (3.8%)	1.0 (0.9-1.2)	0.77	456 (3.8%)	455 (3.8%)	1.0 (0.9-1.1)	0.97
	Gallstones	213 (1.7%)	215 (1.8%)	1.0 (0.8-1.2)	0.92	198 (1.7%)	210 (1.8%)	0.9 (0.8-1.1)	0.54
**Entire Study Period (0-3 Years)**	Osteopenia/Osteoporosis	3,159 (25.7%)	4,584 (37.3%)	0.6 (0.6-0.6)	<0.001	3,064 (25.8%)	4,438 (37.4%)	0.6 (0.6-0.6)	<0.001
	Fractures	1,073 (8.7%)	1,032 (8.4%)	1.0 (1.0-1.1)	0.35	1,017 (8.6%)	997 (8.4%)	1.0 (0.9-1.1)	0.64
	Urolithiasis	1,021 (8.3%)	1,787 (14.6%)	0.5 (0.5-0.6)	<0.001	925 (7.8%)	1,710 (14.4%)	0.5 (0.5-0.5)	<0.001
	MDD	2,469 (20.1%)	2,520 (20.5%)	1.0 (0.9-1.0)	0.42	2,357 (19.8%)	2,438 (20.5%)	1.0 (0.9-1.0)	0.19
	Anxiety Disorders	2,447 (19.9%)	2,256 (18.4%)	1.1 (1.0-1.2)	0.002	2,332 (19.6%)	2,172 (18.3%)	1.1 (1.0-1.2)	0.008
	HTN	8,052 (65.6%)	7,983 (65.0%)	1.0 (1.0-1.1)	0.35	7,732 (65.1%)	7,734 (65.1%)	1.0 (0.9-1.1)	0.98
	GERD	3,355 (27.3%)	3,670 (29.9%)	0.9 (0.8-0.9)	<0.001	3,258 (27.4%)	3,549 (29.9%)	0.9 (0.8-0.9)	<0.001
	Malaise/Fatigue	2,791 (22.7%)	3,087 (25.1%)	0.9 (0.8-0.9)	<0.001	2,655 (22.4%)	2,957 (24.9%)	0.9 (0.8-0.9)	<0.001
	Joint Pain/Myalgias	4,660 (38.0%)	4,733 (38.6%)	1.0 (0.9-1.0)	0.34	4,437 (37.4%)	4,562 (38.4%)	1.0 (0.9-1.0)	0.09
	Constipation	1,688 (13.7%)	1,804 (14.7%)	0.9 (0.9-1.0)	0.03	1,641 (13.8%)	1,739 (14.6%)	0.9 (0.9-1.0)	0.07
	Insomnia	1,241 (10.1%)	1,068 (8.7%)	1.2 (1.1-1.3)	<0.001	1,154 (9.7%)	1,030 (8.7%)	1.1 (1.0-1.2)	0.005
	Polyuria	1,408 (11.5%)	1,559 (12.7%)	0.9 (0.8-1.0)	0.003	1,306 (11.0%)	1,505 (12.7%)	0.9 (0.8-0.9)	<0.001
	Weakness	1,935 (15.8%)	2,113 (17.2%)	0.9 (0.8-1.0)	0.002	1,823 (15.4%)	2,032 (17.1%)	0.9 (0.8-0.9)	<0.001
	Abdominal Pain	3,021 (24.6%)	3,078 (25.1%)	1.0 (0.9-1.0)	0.40	2,865 (24.1%)	2,963 (24.9%)	1.0 (0.9-1.0)	0.14
	Headache	1,694 (13.8%)	1,655 (13.5%)	1.0 (1.0-1.1)	0.47	1,577 (13.3%)	1,592 (13.4%)	1.0 (0.9-1.1)	0.77
	Nausea	1,234 (10.1%)	1,216 (9.9%)	1.0 (0.9-1.1)	0.70	1,135 (9.6%)	1,163 (9.8%)	1.0 (0.9-1.1)	0.54
	Amnesia	1,099 (9.0%)	1,081 (8.8%)	1.0 (0.9-1.1)	0.69	1,010 (8.5%)	1,046 (8.8%)	1.0 (0.9-1.1)	0.41
	Gallstones	535 (4.4%)	550 (4.5%)	1.0 (0.9-1.1)	0.64	511 (4.3%)	534 (4.5%)	1.0 (0.8-1.1)	0.47

Abbreviations: PHP, primary hyperparathyroidism; PTH, parathyroid hormone; MDD, major depressive disorder; HTN, hypertension; GERD, gastroesophageal reflux disease; CI, confidence interval

[a]Statistical significance defined as P <0.003

© 2022 Lorenz FJ et al. *JAMA Network Open.*

eTable 10: Comparing Diagnoses and Symptoms in Patients at High Risk of PHP With Hypercalcemia and PTH ≥65 (n = 14,959) or 100 pg/mL (n = 7,387) Without a Documented Diagnosis With Those Diagnosed With Similar PTH Levels (n = 11,262 for PTH ≥65 and n = 8,192 for ≥100 pg/mL)

	**Diagnosis**	**High-Risk PHP PTH ≥65** (n = 10,336 after matching)	**Diagnosed PTH ≥65** (n = 10,336 after matching)	**Odds Ratio (95% CI)**	[a]***P* value**	**High-Risk PHP PTH ≥100** (n = 5,578 after matching)	**Diagnosed PTH ≥100** (n = 5,578 after matching)	**Odds Ratio (95% CI)**	[a]***P* value**
**0-1 Year**	Osteopenia/Osteoporosis	1,565 (15.1%)	2,303 (22.3%)	0.6 (0.6-0.7)	<0.001	799 (14.3%)	1,256 (22.5%)	0.6 (0.5-0.6)	<0.001
	Fractures	400 (3.9%)	421 (4.1%)	0.9 (0.8-1.1)	0.45	190 (3.4%)	223 (4.0%)	0.8 (0.7-1.0)	0.10
	Urolithiasis	476 (4.6%)	872 (8.4%)	0.5 (0.5-0.6)	<0.001	226 (4.1%)	453 (8.1%)	0.5 (0.4-0.6)	<0.001
	MDD	1,227 (11.9%)	1,283 (12.4%)	1.0 (0.9-1.0)	0.23	562 (10.1%)	641 (11.5%)	0.9 (0.8-1.0)	0.02
	Anxiety Disorders	1,159 (11.2%)	1,093 (10.6%)	1.1 (1.0-1.2)	0.14	543 (9.7%)	553 (9.9%)	1.0 (0.9-1.1)	0.75
	HTN	5,433 (52.6%)	5,430 (52.5%)	1.0 (0.9-1.1)	0.97	2,769 (49.6%)	2,995 (53.7%)	0.9 (0.8-0.9)	<0.001
	GERD	1,752 (17.0%)	1,900 (18.4%)	0.9 (0.8-1.0)	0.007	852 (15.3%)	1,007 (18.1%)	0.8 (0.7-0.9)	<0.001
	Malaise/Fatigue	1,232 (11.9%)	1,366 (13.2%)	0.9 (0.8-1.0)	0.005	600 (10.8%)	723 (13.0%)	0.8 (0.7-0.9)	<0.001
	Joint Pain/Myalgias	1,974 (19.1%)	2,191 (21.2%)	0.9 (0.8-0.9)	<0.001	1,001 (17.9%)	1,165 (20.9%)	0.8 (0.8-0.9)	<0.001
	Constipation	719 (7.0%)	804 (7.8%)	0.9 (0.8-1.0)	0.02	339 (6.1%)	434 (7.8%)	0.8 (0.7-0.9)	<0.001
	Insomnia	514 (5.0%)	456 (4.4%)	1.1 (1.0-1.3)	0.06	239 (4.3%)	247 (4.4%)	1.0 (0.8-1.2)	0.71
	Polyuria	504 (4.9%)	628 (6.1%)	0.8 (0.7-0.9)	<0.001	240 (4.3%)	338 (6.1%)	0.7 (0.6-0.8)	<0.001
	Weakness	810 (7.8%)	953 (9.2%)	0.8 (0.8-0.9)	<0.001	392 (7.0%)	501 (9.0%)	0.8 (0.7-0.9)	<0.001
	Abdominal Pain	1,227 (11.9%)	1,325 (12.8%)	0.9 (0.8-1.0)	0.04	569 (10.2%)	700 (12.5%)	0.8 (0.7-0.9)	<0.001
	Headache	648 (6.3%)	697 (6.7%)	0.9 (0.8-1.0)	0.17	297 (5.3%)	380 (6.8%)	0.8 (0.7-0.9)	<0.001
	Nausea	476 (4.6%)	505 (4.9%)	0.9 (0.8-1.1)	0.34	221 (4.0%)	262 (4.7%)	0.8 (0.7-1.0)	0.06
	Amnesia	450 (4.4%)	485 (4.7%)	0.9 (0.8-1.1)	0.24	206 (3.7%)	263 (4.7%)	0.8 (0.6-0.9)	0.007
	Gallstones	227 (2.2%)	246 (2.4%)	0.9 (0.8-1.1)	0.38	115 (2.1%)	133 (2.4%)	0.9 (0.7-1.1)	0.25
**1-2 Years**	Osteopenia/Osteoporosis	1,622 (15.7%)	2,304 (22.3%)	0.6 (0.6-0.7)	<0.001	812 (14.6%)	1,215 (21.8%)	0.6 (0.6-0.7)	<0.001
	Fractures	354 (3.4%)	354 (3.4%)	1.0 (0.9-1.2)	1.0	168 (3.0%)	188 (3.4%)	0.9 (0.7-1.1)	0.28
	Urolithiasis	414 (4.0%)	704 (6.8%)	0.6 (0.5-0.6)	<0.001	206 (3.7%)	360 (6.5%)	0.6 (0.5-0.7)	<0.001
	MDD	1,183 (11.4%)	1,190 (11.5%)	1.0 (0.9-1.1)	0.88	549 (9.8%)	614 (11.0%)	0.9 (0.8-1.0)	0.04
	Anxiety Disorders	1,060 (10.3%)	999 (9.7%)	1.1 (1.0-1.2)	0.16	503 (9.0%)	512 (9.2%)	1.0 (0.9-1.1)	0.77
	HTN	5,305 (51.3%)	5,185 (50.2%)	1.0 (1.0-1.1)	0.10	2,696 (48.3%)	2,809 (50.4%)	0.9 (0.9-1.0)	0.03
	GERD	1,605 (15.5%)	1,795 (17.4%)	0.9 (0.8-0.9)	<0.001	746 (13.4%)	915 (16.4%)	0.8 (0.7-0.9)	<0.001
	Malaise/Fatigue	924 (8.9%)	1,037 (10.0%)	0.9 (0.8-1.0)	0.007	428 (7.7%)	529 (9.5%)	0.8 (0.7-0.9)	<0.001
	Joint Pain/Myalgias	1,959 (19.0%)	2,018 (19.5%)	1.0 (0.9-1.0)	0.30	975 (17.5%)	1,091 (19.6%)	0.9 (0.8-1.0)	0.005
	Constipation	602 (5.8%)	653 (6.3%)	0.9 (0.8-1.0)	0.14	286 (5.1%)	336 (6.0%)	0.8 (0.7-1.0)	0.04
	Insomnia	435 (4.2%)	420 (4.1%)	1.0 (0.9-1.2)	0.60	201 (3.6%)	231 (4.1%)	0.9 (0.7-1.0)	0.14
	Polyuria	457 (4.4%)	522 (5.1%)	0.9 (0.8-1.0)	0.03	231 (4.1%)	244 (4.4%)	0.9 (0.8-1.1)	0.54
	Weakness	552 (5.3%)	700 (6.8%)	0.8 (0.7-0.9)	<0.001	268 (4.8%)	336 (6.0%)	0.8 (0.7-0.9)	0.004

© 2022 Lorenz FJ et al. *JAMA Network Open.*

	Abdominal Pain	999 (9.7%)	1,097 (10.6%)	0.9 (0.8-1.0)	0.02	468 (8.4%)	568 (10.2%)	0.8 (0.7-0.9)	0.001
	Headache	557 (5.4%)	520 (5.0%)	1.1 (1.0-1.2)	0.25	267 (4.8%)	266 (4.8%)	1.0 (0.8-1.2)	0.96
	Nausea	353 (3.4%)	351 (3.4%)	1.0 (0.9-1.2)	0.94	171 (3.1%)	179 (3.2%)	1.0 (0.8-1.2)	0.66
	Amnesia	394 (3.8%)	376 (3.6%)	1.0 (0.9-1.2)	0.51	188 (3.4%)	217 (3.9%)	0.9 (0.7-1.1)	0.14
	Gallstones	160 (1.5%)	189 (1.8%)	0.8 (0.7-1.0)	0.12	82 (1.5%)	99 (1.8%)	0.8 (0.6-1.1)	0.20
**2-3 Years**	Osteopenia/Osteoporosis	1,748 (16.9%)	2,551 (24.7%)	0.6 (0.6-0.7)	<0.001	862 (15.5%)	1,332 (23.9%)	0.6 (0.5-0.6)	<0.001
	Fractures	395 (3.8%)	384 (3.7%)	1.0 (0.9-1.2)	0.69	178 (3.2%)	193 (3.5%)	0.9 (0.7-1.1)	0.43
	Urolithiasis	399 (3.9%)	712 (6.9%)	0.5 (0.5-0.6)	<0.001	192 (3.4%)	357 (6.4%)	0.5 (0.4-0.6)	<0.001
	MDD	1,243 (12.0%)	1,228 (11.9%)	1.0 (0.9-1.1)	0.75	567 (10.2%)	630 (11.3%)	0.9 (0.8-1.0)	0.05
	Anxiety Disorders	1,123 (10.9%)	1,068 (10.3%)	1.1 (1.0-1.2)	0.21	531 (9.5%)	513 (9.2%)	1.0 (0.9-1.2)	0.56
	HTN	5,427 (52.5%)	5,311 (51.4%)	1.0 (1.0-1.1)	0.11	2,774 (49.7%)	2,883 (51.7%)	0.9 (0.9-1.0)	0.04
	GERD	1,648 (15.9%)	1,835 (17.8%)	0.9 (0.8-0.9)	<0.001	762 (13.7%)	947 (17.0%)	0.8 (0.7-0.9)	<0.001
	Malaise/Fatigue	952 (9.2%)	1,083 (10.5%)	0.9 (0.8-0.9)	0.002	478 (8.6%)	589 (10.6%)	0.8 (0.7-0.9)	<0.001
	Joint Pain/Myalgias	1,955 (18.9%)	2,077 (20.1%)	0.9 (0.9-1.0)	0.03	940 (16.9%)	1,094 (19.6%)	0.8 (0.8-0.9)	<0.001
	Constipation	566 (5.5%)	613 (5.9%)	0.9 (0.8-1.0)	0.16	266 (4.8%)	313 (5.6%)	0.8 (0.7-1.0)	0.04
	Insomnia	451 (4.4%)	444 (4.3%)	1.0 (0.9-1.2)	0.81	211 (3.8%)	223 (4.0%)	0.9 (0.8-1.1)	0.56
	Polyuria	496 (4.8%)	538 (5.2%)	0.9 (0.8-1.0)	0.18	229 (4.1%)	279 (5.0%)	0.8 (0.7-1.0)	0.02
	Weakness	591 (5.7%)	671 (6.5%)	0.9 (0.8-1.0)	0.02	290 (5.2%)	348 (6.2%)	0.8 (0.7-1.0)	0.02
	Abdominal Pain	986 (9.5%)	1,070 (10.4%)	0.9 (0.8-1.0)	0.05	477 (8.6%)	565 (10.1%)	0.8 (0.7-0.9)	0.004
	Headache	517 (5.0%)	556 (5.4%)	0.9 (0.8-1.0)	0.22	245 (4.4%)	275 (4.9%)	0.9 (0.7-1.1)	0.18
	Nausea	337 (3.3%)	363 (3.5%)	0.9 (0.8-1.1)	0.32	150 (2.7%)	171 (3.1%)	0.9 (0.7-1.1)	0.23
	Amnesia	389 (3.8%)	405 (3.9%)	1.0 (0.8-1.1)	0.56	169 (3.0%)	220 (3.9%)	0.8 (0.6-0.9)	0.008
	Gallstones	166 (1.6%)	177 (1.7%)	0.9 (0.8-1.2)	0.55	77 (1.4%)	90 (1.6%)	0.9 (0.6-1.2)	0.31
**Entire Study Period (0-3 Years)**	Osteopenia/Osteoporosis	2,673 (25.9%)	3,911 (37.8%)	0.6 (0.5-0.6)	<0.001	1,382 (24.8%)	2,104 (37.7%)	0.5 (0.5-0.6)	<0.001
	Fractures	834 (8.1%)	876 (8.5%)	0.9 (0.9-1.0)	0.29	393 (7.0%)	462 (8.3%)	0.8 (0.7-1.0)	0.01
	Urolithiasis	795 (7.7%)	1,452 (14.0%)	0.5 (0.5-0.6)	<0.001	398 (7.1%)	756 (13.6%)	0.5 (0.4-0.6)	<0.001
	MDD	1,968 (19.0%)	2,082 (20.1%)	0.9 (0.9-1.0)	0.05	888 (15.9%)	1,098 (19.7%)	0.8 (0.7-0.9)	<0.001
	Anxiety Disorders	1,895 (18.3%)	1,873 (18.1%)	1.0 (0.9-1.1)	0.69	916 (16.4%)	963 (17.3%)	0.9 (0.9-1.0)	0.23
	HTN	6,694 (64.8%)	6,809 (65.9%)	1.0 (0.9-1.0)	0.09	3,485 (62.5%)	3,742 (67.1%)	0.8 (0.8-0.9)	<0.001
	GERD	2,756 (26.7%)	3,102 (30.0%)	0.8 (0.8-0.9)	<0.001	1,354 (24.3%)	1,647 (29.5%)	0.8 (0.7-0.8)	<0.001
	Malaise/Fatigue	2,210 (21.4%)	2,566 (24.8%)	0.8 (0.8-0.9)	<0.001	1,079 (19.3%)	1,377 (24.7%)	0.7 (0.7-0.8)	<0.001
	Joint Pain/Myalgias	3,670 (35.5%)	3,987 (38.6%)	0.9 (0.8-0.9)	<0.001	1,860 (33.3%)	2,132 (38.2%)	0.8 (0.7-0.9)	<0.001
	Constipation	1,361 (13.2%)	1,522 (14.7%)	0.9 (0.8-1.0)	0.001	654 (11.7%)	805 (14.4%)	0.8 (0.7-0.9)	<0.001
	Insomnia	940 (9.1%)	888 (8.6%)	1.1 (1.0-1.2)	0.20	439 (7.9%)	469 (8.4%)	0.9 (0.8-1.1)	0.30
	Polyuria	1,124 (10.9%)	1,332 (12.9%)	0.8 (0.8-0.9)	<0.001	531 (9.5%)	696 (12.5%)	0.7 (0.7-0.8)	<0.001
	Weakness	1,474 (14.3%)	1,779 (17.2%)	0.8 (0.7-0.9)	<0.001	730 (13.1%)	933 (16.7%)	0.7 (0.7-0.8)	<0.001
	Abdominal Pain	2,334 (22.6%)	2,565 (24.8%)	0.9 (0.8-0.9)	<0.001	1,118 (20.0%)	1,352 (24.2%)	0.8 (0.7-0.9)	<0.001

© 2022 Lorenz FJ et al. *JAMA Network Open.*

	Headache	1,303 (12.6%)	1,357 (13.1%)	1.0 (0.9-1.0)	0.26	612 (11.0%)	714 (12.8%)	0.8 (0.7-0.9)	0.003
	Nausea	889 (8.6%)	983 (9.5%)	0.9 (0.8-1.0)	0.02	429 (7.7%)	490 (8.8%)	0.9 (0.8-1.0)	0.04
	Amnesia	885 (8.6%)	922 (8.9%)	1.0 (0.9-1.1)	0.36	417 (7.5%)	508 (9.1%)	0.8 (0.7-0.9)	0.002
	Gallstones	431 (4.2%)	471 (4.6%)	0.9 (0.8-1.0)	0.17	218 (3.9%)	242 (4.3%)	0.9 (0.7-1.1)	0.25

Abbreviations: PHP, primary hyperparathyroidism; PTH, parathyroid hormone; MDD, major depressive disorder; HTN, hypertension; GERD, gastroesophageal reflux disease; CI, confidence interval

[a]Statistical significance defined as *P* <0.003

© 2022 Lorenz FJ et al. *JAMA Network Open.*
