# Supplementary material for: Analysis of Time to Diagnosis and Outcomes Among Adults With Primary Hyperparathyroidism
Source: JAMA Netw Open. 2022 Dec 27;5(12):e2248332. doi: 10.1001/jamanetworkopen.2022.48332 (PMC9857508; doi:10.1001/jamanetworkopen.2022.48332)
Supplement: Supplement 2. — Data Sharing Statement [file jamanetwopen-e2248332-s002.pdf]

Data Sharing Statement

Lorenz. Analysis of Time to Diagnosis and Outcomes Among Adults With Primary Hyperparathyroidism. *JAMA Netw Open*. Published December 27, 2022. doi:10.1001/jamanetworkopen.2022.48332

Data
Data available: Yes
Data types: Data dictionary, Other (please specify)
Additional Information: Deidentified analyses performed via the TriNetX online platform.
How to access data: The data from the current study is available from the author, F. Jeffrey Lorenz (florenz@pennstatehealth.psu.edu), upon reasonable request.
When available: With publication

Supporting Documents
Document types: None

Additional Information
Who can access the data: Anyone requesting the data
Types of analyses: For any purpose
Mechanisms of data availability: With investigator support
Any additional restrictions: None
